# Design, synthesis, *in vitro* anticancer evaluation, kinase inhibitory effects, and pharmacokinetic profile of new 1,3,4-triarylpyrazole derivatives possessing terminal sulfonamide moiety

**DOI:** 10.1080/14756366.2018.1530225

**Published:** 2018-10-26

**Authors:** Mohammed S. Abdel-Maksoud, Mohammed I. El-Gamal, Mahmoud M. Gamal El-Din, Chang Hyun Oh

**Affiliations:** a Medicinal & Pharmaceutical Chemistry Department, Pharmaceutical and Drug Industries Research Division, National Research Centre (NRC), Dokki, Giza, Egypt;; b Department of Medicinal Chemistry, College of Pharmacy, University of Sharjah, Sharjah, United Arab Emirates;; c Sharjah Institute for Medical Research, University of Sharjah, Sharjah, United Arab Emirates;; d Department of Medicinal Chemistry, Faculty of Pharmacy, University of Mansoura, Mansoura, Egypt;; e Center for Biomaterials, Korea Institute of Science and Technology (KIST), Cheongryang, Seoul, Republic of Korea;; f Department of Biomolecular Science, University of Science and Technology (UST), Daejeon, Yuseong-guRepublic of Korea

**Keywords:** Anticancer, kinase inhibitor, pharmacokinetic, pyrazole, sulfonamide

## Abstract

The present work describes the design and synthesis of a novel series of 1,3-diaryl-4-sulfonamidoarylpyrazole derivatives **1a–q** and **2a–q** and their *in vitro* biological activities. The target compounds were evaluated for antiproliferative activity against NCI-60 cell line panel. Compounds **1c, 1g, 1k–m, 1o, 2g, 2h, 2k–m, 2o**, and **2q** showed the highest mean inhibition percentages at 10 µM single-dose testing and were selected to be tested at 5-dose mode. The ICs_50_ of the most potent compounds were determined over the 60 cell lines. Compound **2l** exhibited the strongest activity against different cell lines with IC_50_ 0.33 µM against A498 renal cancer cell line. Compound **2l** was tested over a panel of 20 kinases to determine its molecular target(s), and its IC_50_ values over the most sensitive kinases were defined. *In vitro* stability and *in vivo* pharmacokinetic profile of compound **2l** was also investigated.

## Introduction

Cancer is one of the most extensively spreading diseases around the world. In 2015, one in each six global death cases occurred as a result of different cancer types^1^. According to American Cancer Society, everyday there are 4750 new cancer cases and 1670 death cases^2^. The global cancer statistics revealed that cancer is the second fatal condition after cardiovascular diseases^3^^,^^4^. Despite the rapid development in the diagnostic area, development of new cancer therapy is a quite challenging mission due to the sophisticated biological pathways contributing to cancer progression.

**Figure 1.**
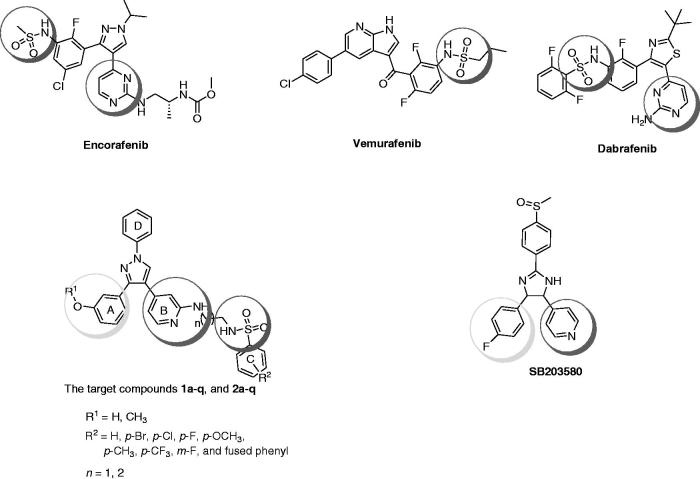
Structures of encorafenib, vemurafenib, dabrafenib, SB203580, and the target compounds **1a–q** and **2a–q**.

Many compounds possessing sulfonamide moiety have been reported as highly effective antiproliferative agents^5–7^. Vemurafenib (Zelboraf®, Figure 1) is the first kinase inhibitor drug possessing sulfonamide moiety to be approved by the FDA in 2011 for the treatment of late-stage melanoma. Vemurafenib acts through the inhibition of mutated B-RAF^8^. Dabrafenib (Tafinlar^®^, Figure 1) is another targeted therapy, which possesses sulfonamide moiety^9^. Dabrafenib was approved in 2013 for the treatment of mutant B-RAF (V600E-B-RAF)-associated proliferative disorders^10^. Encorafenib (LGX 818, Figure 1) is a drug candidate that carries both sulfonamide and pyrazole backbone which has been recently approved by the FDA to be used in combination with binimetinib for the treatment of unresectable or metastatic melanoma^11^.

Based on the abovementioned structures and our previous pyrazole ring anticancer investigations^7^^,^^12–21^, a novel series of 1,3,4-triarylpyrazole was designed and synthesized in which the structure of both rings B and D were fixed and diverse structure modifications were performed in both rings A and C (Figure 1). The connection length between ring B and sulfonamide terminal moiety ring C was selected from two main chains, ethylene and propylene. The target compounds were tested for *in vitro* antiproliferative activity against the standard NCI-60 cancer cell line panel. The structure–activity relationships (SAR) are explained in details to show the effects of sulfonamide, linker length, and different substituents on the biological activity. The most promising compounds were further tested against a panel of kinases to study their molecular mechanism of action. The *in vitro* stability and *in vivo* pharmacokinetic profile were also investigated for the most potent compound. The synthetic and biological procedures, as well as relevant discussions, are presented in details.

## Experimental

### Chemistry

#### General

All solvents and reagents were commercially available and used as such with no further purification. The target compounds and intermediates were purified by column chromatography using silica gel (0.040–0.063 mm, 230–400 mesh) and technical grade solvents. Analytical thin-layer chromatography (TLC) was adopting on silica gel 60 F_254_ plates from Merck. Purity percentages of the target compounds were confirmed to be more than 96% by LC-MS. ^1^H NMR and ^13^C NMR spectra were recorded on a Bruker Avance 400 or 300 spectrometer using tetramethylsilane as an internal standard and signals are described as s (singlet), d (doublet), t (triplet), q (quartet), p (pentet), m (multiplet), brs (broad singlet), or dd (doublet of doublets). LC-MS analysis was carried out using the following system: Waters 2998 photodiode array detector, Waters 3100 mass detector, Waters SFO system fluidics organizer, Waters 2545 binary gradient module, Waters reagent manager, Waters 2767 sample manager, Sunfire™ C18 column (4.6 × 50 mm, 5 µm particle size); Solvent gradient = 95% A at 0 min, 1% A at 5 min; solvent A: 0.035% trifluoroacetic acid (TFA) in water; solvent B: 0.035% TFA in CH_3_OH; flow rate = 3.0 mL/min; the AUC was calculated using Waters MassLynx 4.1 software. Solvents and liquid reagents were transferred using hypodermic syringes. Melting points were obtained on a Walden Precision Apparatus Electro thermal 9300 apparatus and are uncorrected.

#### Synthesis of N^1^-(4-(3-(3-methoxyphenyl)-1-phenyl-1H-pyrazol-4-yl)pyridin-2-yl)ethane1,2-diamine (8) and N^1^-(4-(3-(3-methoxyphenyl)-1-phenyl-1H-pyrazol-4-yl)pyridin-2-yl)propane-1,3-diamine (9)

They were synthesized utilizing the five-step procedure reported in the literature^19^. The detailed procedures are also mentioned in the supplementary file.

#### General procedure for synthesis of the target compounds N-(2-((4-(3-(3-methoxyphenyl)-1-phenyl-1H-pyrazol-4-yl)pyridin-2-yl)amino)ethyl)arylsulfonamides (1a-i) and N-(3-((4-(3-(3-methoxyphenyl)-1-phenyl-1H-pyrazol-4-yl)pyridin-2-yl)amino)propyl)arylsulfonamides (2a–i)

To a solution of compound **8** or **9** (0.2 mmol) in anhydrous dichloromethane (5 mL), triethylamine (50.5 mg, 0.5 mmol) was added at 0 °C. A solution appropriate arylsulfonyl chloride (0.21 mmol) in anhydrous dichloromethane (1 mL) was added thereto dropwise. The reaction mixture was stirred at room temperature for 24 h. When the reaction completed, the solvent was removed under vacuo, and the residue was partitioned between ethyl acetate (5 mL) and water (5 mL). The organic layer was separated and the aqueous layer was extracted with ethyl acetate (3 × 10 mL). The combined organic layer was washed with saturated saline (2 × 5 mL) and the organic solvent was evaporated under reduced pressure. The residue was purified by column chromatography (silica gel, hexane-ethyl acetate 4:1 v/v) to give the required product.

#### N-(2-((4-(3-(3-methoxyphenyl)-1-phenyl-1H-pyrazol-4-yl)pyridin-2-yl)amino)ethyl) benzenesulfonamide (1a)

White solid (65%); mp 104–6 °C; ^1^H NMR (300 MHz, CDCl_3_) *δ* 7.93 (s, 1H, Ar-H), 7.86–7.79 (m, 4H, Ar-H), 7.51–7.40 (m, 3H, Ar-H), 7.25–7.21 (m, 5H, Ar-H), 6.92–6.88 (m, 1H, Ar-H), 6.77 (d, *J* = 9.0 Hz, 1H, Ar-H), 6.70 (s, 1H, Ar-H), 6.42 (d, *J* = 6.0 Hz, 1H, Ar-H), 6.24 (s, 1H, Ar-H), 4.98 (brs, 1H, NH), 3.65 (s, 3H, OCH_3_), 3.32 (d, *J* = 3.0 Hz, 2H, CH_2_), 3.09 (d, *J* = 6.0 Hz, 2H, CH_2_); ^13^C NMR (75 MHz, CDCl_3_) *δ* 159.6, 158.6, 147.4, 141.9, 140.1, 140.1, 139.5, 139.4, 132.4, 131.0, 130.0, 129.9, 128.9, 128.8, 127.6, 126.9, 125.1, 122.7, 120.0, 115.8, 114.7, 112.4, 105.9 (Ar-C), 55.3 (OCH_3_), 43.9 (CH_2_), 41.6 (CH_2_).; LC-MS(*m/z*) calculated for C_29_H_27_N_5_O_3_S: 525.18, found: 526.0 (M + 1)^+^.

#### 4-Bromo-N-(2-((4-(3-(3-methoxyphenyl)-1-phenyl-1H-pyrazol-4-yl)pyridin-2-yl)amino)ethyl) benzenesulfonamide (1b)

White solid (61%); mp 136–8 °C; ^1^H NMR (300 MHz, CDCl_3_) *δ* 7.96 (s, 1H, Ar-H), 7.89 (d, *J* = 6.0 Hz, 1H, Ar-H), 7.64 (d, *J* = 9 Hz, 2H, Ar-H), 7.58–7.55 (m, 2H, Ar-H),7.31–7.28 (m, 6H, Ar-H), 6.93 (d, *J* = 6.0 Hz, 1H, Ar-H), 6.81 (d, *J* = 9.0 Hz, 1H, Ar-H), 6.72 (s,1H, Ar-H), 6.48 (d, *J* = 6.0 Hz, 1H, Ar-H), 6.24 (s, 1H, Ar-H), 4.81 (brs, 1H, NH), 3.69 (s, 3H, OCH_3_), 3.36 (brs, 2H, CH_2_), 3.12 (brs, 2H, CH_2_); ^13^C NMR (75 MHz, CDCl_3_) *δ* 159.7, 158.5, 147.2, 142.2, 140.2, 139.5, 139.3, 139.2, 132.6, 132.6, 132.1, 131.0, 130.0, 128.8, 128.5, 127.6, 127.1, 125.1, 122.7, 119.8, 115.8, 114.8, 112.6, 106.1 (Ar-C), 55.3 (OCH_3_), 44.4 (NH–CH_2_), 41.6 (CH_2_–NH); LC-MS(*m/z*) calculated for C_29_H_26_BrN_5_O_3_S: 603.09, found: 605.0 (M + 2)^+^.

#### 4-Chloro-N-(2-((4-(3-(3-methoxyphenyl)-1-phenyl-1H-pyrazol-4-yl)pyridin-2-yl)amino)ethyl) benzenesulfonamide (1c)

White solid (60%); mp 132–4 °C; ^1^H NMR (300 MHz, CDCl_3_) *δ* 7.94 (s, 1H, Ar-H), 7.85 (d, *J* = 6.0 Hz, 1H, Ar-H), 7.72–7.69 (m, 2H, Ar-H), 7.54–7.51 (m, 2H, Ar-H), 7.39–7.22 (m, 6H, Ar-H), 6.91 (d, *J* = 9.0 Hz, 1H, Ar-H), 6.78 (d, *J* = 9.0 Hz, 1H, Ar-H), 6.70 (s, 1H, Ar-H), 6.43 (d, *J* = 3.0 Hz, 1H, Ar-H), 6.24 (s, 1H, Ar-H), 4.95 (brs, 1H, NH), 3.66 (s, 3H, OCH_3_), 3.33 (d, *J* = 3.0 Hz, 2H, NH–CH2), 3.09 (d, *J* = 3.0 Hz, 2H, –CH_2_–NH); ^13^C NMR (75 MHz, CDCl_3_) *δ* 159.6, 158.5, 147.3, 142.0, 140.1, 139.5, 139.3, 138.6, 131.0, 129.9, 129.1, 128.8, 127.6, 125.1, 122.7, 119.9, 115.8, 114.7, 112.5, 106.9 (Ar-C), 55.2 (OCH_3_), 44.1 (NH-CH_2_), 41.5 (–CH_2_-NH).; LC-MS(*m/z*) calculated for C_29_H_26_ClN_5_O_3_S: 559.14, found: 560.0 (M + 1)^+^.

#### 4-Fluoro-N-(2-((4-(3-(3-methoxyphenyl)-1-phenyl-1H-pyrazol-4-yl)pyridin-2-yl)amino)ethyl) benzenesulfonamide (1d)

White solid (67%); mp155-6 °C; ^1^H NMR (400 MHz, CDCl_3_) *δ* 7.83 (s, 1H, Ar-H), 7.71–7.67 (m, 3H, Ar-H), 7.19–7.15 (m, 5H, Ar-H), 6.99 (t, *J* = 8.4 Hz, 2H, Ar-H), 6.80 (d, *J* = 8.4 Hz, 1H, Ar-H), 6.67 (d, *J* = 8.8 Hz, 1H, ar-H), 6.59 (s, 1H, Ar-H), 6.34 (d, *J* = 5.2 Hz, 1H, Ar-H), 6.13 (s, 1H, Ar-H), 4.85 (brs, NH), 3.56 (s, 3H, OCH_3_), 3.23 (s, 2H, NH–CH_2_), 2.98 (d, *J* = 4.4 Hz, 2H, –CH_2_–NH); ^13^C NMR (100 MHz, CDCl_3_) *δ* 159.7, 158.6, 147.1, 142.2, 140.1, 139.5, 139.3, 136.1, 130.9, 130.0, 129.7, 129.6, 129.5, 128.9, 128.7, 125.2, 125.0, 122.7, 119.8, 116.0, 115.0, 112.5, 105.9 (Ar-C), 55.3 (OCH_3_), 44.2 (CH_2_), 41.6 (CH_2_); LC-MS(*m/z*) calculated for C_29_H_26_FN_5_O_3_S: 543.17, found: 544.0 (M + 1)^+^.

#### 4-Methoxy-N-(2-((4-(3-(3-methoxyphenyl)-1-phenyl-1H-pyrazol-4-yl)pyridin-2-yl)amino)ethyl)benzenesulfonamide (1e)

White solid (62%); mp 144–6 °C; ^1^H NMR (400 MHz, CDCl_3_) *δ* 7.94 (s, 1H, Ar-H),7.88 (d, *J=* 4.0 Hz, 1H, Ar-H), 7.74 (d, *J* = 8.0 Hz, 2H, Ar-H), 7.33–7.23 (m, 6H, Ar-H), 6.91 (d, *J* = 8.0 Hz, 3H, Ar-H), 6.79–6.77 (m, 1H, Ar-H), 6.70–6.69 (m, 1H, Ar-H), 6.43 (dd, *J=* 8.0, *J* = 4.0 Hz, 1H, Ar-H), 6.23 (s, 1H, Ar-H), 4.85 (s, NH), 3.83 (s, 3H, OCH_3_), 3.67 (s, 3H, OCH_3_), 3.34 (t, *J* = 8.0 Hz, 2H, NH–CH_2_–), 3.08 (t, *J* = 8.0 Hz, 2H, –CH_2_–NH); ^13^C NMR (100 MHz, CDCl_3_) *δ* 162.6, 159.6, 158.6, 147.5, 141.9, 140.1, 139.5, 139.4, 131.6, 131.0, 129.9, 129.1, 128.8, 127.6, 125.1, 122.7, 120.0, 115.7, 114.7, 114.1, 112.4, 105.9 (Ar-C), 55.5 (OCH_3_), 55.3 (OCH_3_), 43.8 (CH_2_), 41.5 (CH_2_); LC-MS(*m/z*) calculated for C_30_H_29_N5O_4_S: 555.19, found: 556.0 (M + 1)^+^.

#### N-(2-((4-(3-(3-Methoxyphenyl)-1-phenyl-1H-pyrazol-4-yl)pyridin-2-yl)amino)ethyl)-4-methylbenzenesulfonamide (1f)

White solid (69%); mp 150–2 °C; ^1^H NMR (300 MHz, CDCl_3_) *δ* 7.97 (s, 1H, Ar-H),7.91 (d, *J* = 6.0 Hz, 1H), 7.71 (d, *J* = 9.0 Hz, 2H, Ar-H), 7.32–7.26 (m, 7H), 6.94 (d, *J* = 9.0 Hz, 1H, Ar-H), 6.81 (d, *J=* 6.0 Hz, 1H, Ar-H), 6.72 (s, 1H, Ar-H), 6.49 (d, *J=* 3.0 Hz, 1H, Ar-H), 6.26 (s, 1H Ar-H), 4.97 (brs, 1H, NH), 3.70 (s, 3H, OCH_3_), 3.36 (brs, 2H, NH–CH_2_–), 3.12 (t, *J* = 6.0 Hz, 2H, –CH_2_–NH), 2.43 (s, 3H, CH_3_); ^13^C NMR (75 MHz, CDCl_3_) *δ* 159.7, 158.5, 147.4, 143.1, 142.1, 140.1, 139.6, 139.3, 137.1, 131.1, 129.9, 129.5, 128.8, 127.6, 127.0, 125.1, 122.7, 120.0, 115.7, 114.7, 112., 105.5 (Ar-C), 55.3 (OCH3), 44.0 (CH_2_), 41.7 (CH_2_), 21.4 (CH_3_); LC-MS(*m/z*) calculated for C_30_H_29_N_5_O_3_S: 539.20, found: 540.0 (M + 1)^+^.

#### N-(2-((4-(3-(3-Methoxyphenyl)-1-phenyl-1H-pyrazol-4-yl)pyridin-2-yl)amino)ethyl)-4-(trifluoromethyl)benzenesulfonamide (1g)

White solid (74%); mp 132–4 °C; ^1^H NMR (300 MHz, CDCl_3_) *δ* 7.91 (d, *J* = 9.0 Hz, 4H, Ar-H), 7.71 (s, 1H, Ar-H), 7.30 (brs, 6H, Ar-H), 6.93 (s, 1H, Ar-H), 6.80 (s, 1H, Ar-H), 6.71 (s, 2H, Ar-H), 6.48 (s, 1H, Ar-H), 6.25 (s, 1H, Ar-H), 4.87 (brs, 1H, NH), 3.67 (s, 3H, OCH_3_), 3.37 (s, 2H, NH–CH_2_–), 3.14 (s, 2H, –CH_2_–NHSO_2_); ^13^C NMR (75 MHz, CDCl_3_) *δ* 159.7, 158.5, 147.1, 147.0, 143.8, 142.4, 139.5, 139.4, 139.2, 134.1, 133.7, 130.9, 130.1, 129.9, 128.9, 127.4, 126.0, 125.2, 125.0, 122.7, 119.7, 115.8, 114.7, 112.8, 106.2 (Ar-C), 55.3 (OCH_3_), 44.7 (CH_2_), 41.7 (CH_2_); LC-MS(*m/z*) calculated for C_30_H_26_F_3_N_5_O_3_S: 593.17, found: 594.0 (M + 1)^+^.

#### 3-Fluoro-N-(2-((4-(3-(3-methoxyphenyl)-1-phenyl-1H-pyrazol-4-yl)pyridin-2-yl)amino)ethyl)benzenesulfonamide (1h)

White solid (66%); mp 118–20 °C; ^1^H NMR (300 MHz, CDCl_3_) *δ* 7.94 (s, 1H, Ar-H), 7.85 (d, *J* = 6.0 Hz, 1H, Ar-H), 7.59 (s, 1H, Ar-H), 7.49 (s, 1H, Ar-H), 7.46–7.37 (m, 2H, Ar-H), 7.29–7.21 (m, 6 H), 6.90 (d, *J* = 9.0 Hz, 1H, Ar-H), 6.78 (d, *J* = 9.0 Hz, 1H, Ar-H), 6.71 (s, 1H), 6.43 (d, *J* = 6.0 Hz, 1H, Ar-H), 6.25 (s, 1H, Ar-H), 4.99 (brs, 1H, NH), 3.65 (s, 3H, OCH_3_), 3.33 (brs, 2H, NH–CH_2_–), 3.10 (t, *J* = 6.0 Hz, 2H, –CH_2_–NH–SO_2_); ^13^C NMR (75 MHz, CDCl_3_) *δ* 159.6, 158.6, 147.3, 142.2, 142.0, 140.2, 139.5, 139.4, 130.9, 130.8, 130.7, 129.9, 128.8, 127.6, 125.1, 122.7, 119.9, 119.6, 119.3, 115.7, 114.7, 114.4, 114.1, 112.5, 106.0 (Ar-C), 55.2 (OCH_3_), 44.2 (CH_2_), 41.5 (CH_2_); LC-MS(*m/z*) calculated for C_29_H_26_FN_5_O_3_S: 543.17, found: 544.0 (M + 1)^+^.

#### N-(2-((4-(3-(3-Methoxyphenyl)-1-phenyl-1H-pyrazol-4-yl)pyridin-2-yl)amino)ethyl)naphthalene-1-sulfonamide (1i)

White solid (71%); mp 178–80 °C; ^1^H NMR (400 MHz, CDCl_3_) *δ*; 8.38 (s, 1H, Ar-H), 7.91–7.85 (m, 4H, Ar-H), 7.75 (dd, *J* = 8.8 Hz, *J* = 1.6 Hz, 1H, Ar-H), 7.64–7.55 (m, 2H, Ar-H), 7.34–7.21 (m, 6H, Ar-H), 7.02 (brs, 1H, NH), 6.89 (dd, *J* = 8.4 Hz, *J* = 2.0 Hz, 1H, Ar-H), 6.76 (d, *J* = 7.6 Hz, 1H, Ar-H), 6.68 (s, 1H, Ar-H), 6.42 (d, *J* = 4.8 Hz, 1H, Ar-H), 6.17 (s, 1H, Ar-H), 4.87 (brs, 1H, NH), 3.65 (s, 3H, OCH_3_), 3.34 (d, *J* = 4.4 Hz, 2H, NH–CH_2_–), 3.14 (t, *J* = 5.2 Hz, 2H, –CH_2_–NH–SO_2)_; ^13^C NMR (100 MHz, CDCl_3_) *δ* 159.6, 158.4, 147.1, 142.1, 140.1, 139.5, 139.4, 136.8, 134.6, 132.1, 130.9, 129.8, 129.3, 129.1, 128.8, 128.6, 128.2, 127.8, 127.6, 127.4, 125.1, 122.7, 122.3, 119.9, 115.7, 114.7, 112.4, 105.9 (Ar-C), 55.2 (OCH_3_), 44.3 (CH_2_), 41.5 (CH_2_); LC-MS(*m/z*) calculated for C_33_H_29_N_5_O_3_S: 575.20, found: 576.0 (M + 1)^+^.

#### N-(3-((4-(3-(3-Methoxyphenyl)-1-phenyl-1H-pyrazol-4-yl)pyridin-2-yl)amino)propyl) benzenesulfonamide (2a)

White solid (60%); mp 119–20 °C; ^1^H NMR (300 MHz, CDCl_3_) *δ* 7.98 (d, *J* = 12.0 Hz, 2H, Ar-H), 7.83 (d, *J* = 9.0 Hz, 2H, Ar-H), 7.52–7.45 (m, 3H, Ar-H), 7.31–7.24 (m, 6H, Ar-H), 6.93 (d, *J* = 6.0 Hz, 1H, Ar-H), 6.79 (d, *J* = 9.0 Hz, 1H, Ar-H), 6.71 (s, 1H, Ar-H), 6.45 (d, *J* = 3.0 Hz, 1H, Ar-H), 6.22 (s, 1H, Ar-H), 4.73 (brs, 1H, NH), 3.68 (s, 3H, OCH_3_), 3.31 (brs, 2H, NH–CH_2_–), 2.97 (brs, 2H, –CH_2_–NH–SO_2_), 1.67–1.58 (m, 2H, –CH_2_–); ^13^C NMR (75 MHz, CDCl_3_) *δ* 159.6, 158.7, 147.2, 142.1, 140.4, 140.1, 139.5, 139.3, 132.2, 131.0, 129.9, 128.9, 128.8, 127.6, 126.9, 125.1, 122.7, 120.0, 115.7, 114.8, 112.1, 105.7 (Ar-C), 55.3 (OCH_3_), 40.1 (CH_2_), 38.3 (CH_2_), 29.9 (CH_2_); LC-MS(*m/z*) calculated for C_30_H_29_N_5_O_3_S: 539.20, found: 540.0 (M + 1)^+^.

#### 4-Bromo-N-(3-((4-(3-(3-methoxyphenyl)-1-phenyl-1H-pyrazol-4-yl)pyridin-2-yl)amino)propyl) benzenesulfonamide (2b)

White solid (62%); mp 117–19 °C; ^1^H NMR (400 MHz, CDCl_3_) *δ* 7.98 (s, 1H, Ar-H), 7.90 (d, *J* = 5.6 Hz, 1H, Ar-H), 7.69 (d, *J* = 8.8 Hz, 2H, Ar-H), 7.60 (d, *J* = 8.8 Hz, 2H, Ar-H), 7.35–7.20 (m, 6H, Ar-H), 6.95 (dd, *J* = 8.0, *J* = 2.0 Hz, 1H, Ar-H), 6.72 (t, *J* = 2.0 Hz, 1H, Ar-H), 6.49 (dd, *J* = 6.0 Hz, *J* = 1.6 Hz, 1H, Ar-H), 6.28 (s, 1H, Ar-H), 5.21 (brs, 1H, NH), 3.70 (s, 3H, OCH_3_), 3.36 (q, *J* = 6.4 Hz, 2H, NH-CH_2_-), 2.99 (d, *J* = 5.6 Hz, 2H, –CH_2_NHSO_2_), 1.68 (p, *J* = 6.4 Hz, 2H, CH_2_–CH_2_–CH_2_); ^13^C NMR (75 MHz, CDCl_3_) *δ* 159.7, 158.9, 147.3, 142.1, 140.2, 139.6, 139.4, 132.2, 131.0, 130.0, 128.8, 128.5, 127.6, 127.1, 125.1, 122.8, 120.0, 115.8, 114.8, 112.1, 105.9 (Ar-C), 55.3 (OCH_3_), 40.2 (CH_2_), 38.3(CH_2_), 29.9(CH_2_); LC-MS(*m/z*) calculated for C_30_H_28_BrN_5_O_3_S: 617.11, found: 618.00 (M + 1)^+^.

#### 4-Chloro-N-(3-((4-(3-(3-methoxyphenyl)-1-phenyl-1H-pyrazol-4-yl)pyridin-2-yl)amino)propyl) benzenesulfonamide (2c)

White solid (62%); mp 98–100 °C; ^1^H NMR (400 MHz, CDCl_3_) *δ* 7.95 (s, 1H, Ar-H), 7.93 (d, *J* = 5.2 Hz, 1H, Ar-H), 7.75 (d, *J* = 8.4 Hz, 2H, Ar-H), 7.42 (d, *J* = 8.4 Hz, 2H, Ar-H), 7.32–7.25 (m, 6H, Ar-H), 6.93 (dd, *J* = 8.0 Hz, *J* = 2.0 Hz, 1H, Ar-H), 6.79 (d, *J* = 7.6 Hz, 1H, Ar-H), 6.71 (s, 1H, Ar-H), 6.45 (d, *J* = 5.6 Hz, 1H, Ar-H), 6.25 (s, 1H, Ar-H), 4.84 (brs, 1H, NH), 3.68 (s, 3H, OCH_3_), 3.34 (d, *J* = 5.6 Hz, 2H, NH–CH_2_–), 2.90 (brs, 2H, –CH_2_NHSO_2_), 1.66 (t, *J* = 6.0 Hz, 2H, -CH_2_-); ^13^C NMR (100 MHz, CDCl_3_) *δ* 159.4, 158.4, 146.8, 142.8, 140.2, 139.3, 139.3, 138.9, 138.6, 130.9, 130.0, 129.5, 129.2, 128.8, 128.4, 127.6, 125.1, 122.7, 119.8, 115.7, 114.8, 112.0, 105.9 (Ar-C), 55.2 (OCH_3_), 40.0 (CH_2_), 38.2 (CH_2_), 29.9 (CH_2_); LC-MS(*m/z*) calculated for C_30_H_28_ClN_5_O_3_S: 573.16, found: 574.0 (M + 1)^+^.

#### 4-Fluoro-N-(3-((4-(3-(3-methoxyphenyl)-1-phenyl-1H-pyrazol-4-yl)pyridin-2-yl)amino)propyl) benzenesulfonamide (2d)

White solid (75%); mp 92–94 °C; ^1^H NMR (300 MHz, CDCl_3_) *δ* 7.96 (s, 2H, Ar-H), 7.82 (d, *J* = 3.0 Hz, 2H, Ar-H), 7.32–7.28 (m, 6H, Ar-H), 7.14 (t, *J* = 9.0 Hz, 2H, Ar-H), 6.94 (d, *J* = 6.0 Hz, 1H, Ar-H), 6.80 (d, *J* = 6.0 Hz, 1H, Ar-H), 6.72 (s, 1H, ar-H), 6.46 (d, *J* = 6.0 Hz, 1H, Ar-H), 6.24 (s, 1H, Ar-H), 4.60 (brs, 1H, NH), 3.69 (s, 3H, OCH_3_), 3.36 (s, 2H, NH–CH_2_–), 2.98 (brs, 2H, –CH_2_NHSO_2_), 1.66 (d, *J* = 6.0 Hz, 2H, –CH_2_–); ^13^C NMR (75 MHz, CDCl_3_) *δ* 159.6, 158.7, 147.2, 140.1, 139.3, 131.0, 129.9, 129.6, 129.5, 128.8, 127.6, 125.1, 122.7, 119.9, 116.2, 115.9, 115.7, 114.8, 112.2, 106.0 (Ar-C), 55.2 (OCH_3_), 40.0 (CH_2_), 38.2 (CH_2_), 30.0 (CH_2_); LC-MS(*m/z*) calculated for C_30_H_28_FN_5_O_3_S: 557.19, found: 558.0 (M + 1)^+^.

#### 4-Methoxy-N-(3-((4-(3-(3-methoxyphenyl)-1-phenyl-1H-pyrazol-4-yl)pyridin-2-yl)amino) propyl)benzenesulfonamide (2e)

White solid (71%); mp 124–6 °C; ^1^H NMR (300 MHz, CDCl_3_) *δ* 7.96 (s, 1H, Ar-H), 7.94 (d, *J* = 6.0 Hz, 1H, Ar-H), 7.76 (d, *J* = 9.0 Hz, 2H, Ar-H),7.33–7.24 (m, 6H, Ar-H), 6.93 (d, *J* = 9.0 Hz, 3H, Ar-H), 6.79 (d, *J* = 9.0 Hz, 1H, Ar-H), 6.71 (s, 1H, Ar-H), 6.54 (brs, 1H, NH), 6.45 (d, *J* = 3.0 Hz, 1H, Ar-H), 6.22 (s, 1H, Ar-H), 4.61 (brs, 1H, NH), 3.86 (s, 3H, OCH_3_), 3.68 (s, 3H, OCH_3_), 3.32 (t, *J* = 6.0 Hz, 2H, NH–CH_2_–), 2.96 (t, *J* = 6.0 Hz, 2H, –CH_2_NHSO_2_), 1.65 (p, *J* = 6.0 Hz, 2H, –CH_2_–); ^13^C NMR (75 MHz, CDCl_3_) *δ* 159.7, 158.8, 147.5, 142.0, 140.1, 139.6, 139.4, 132.0, 131.1, 130.0, 128.8, 127.6, 125.1, 122.8, 120.1, 115.7, 114.8, 114.1, 112.2, 105.7(Ar-C), 55.6 (OCH_3_), 55.3 (OCH_3_), 40.2 (CH_2_), 38.4(CH_2_), 30.0 (CH_2_); LC-MS(*m/z*) calculated for C_31_H_31_N_5_O_3_S: 569.21, found: 570.0 (M + 1)^+^.

#### N-(3-((4-(3-(3-Methoxyphenyl)-1-phenyl-1H-pyrazol-4-yl)pyridin-2-yl)amino)propyl)-4-methylbenzenesulfonamide (2f)

Buff solid (72%); mp 94–6 °C; ^1^H NMR (300 MHz, CDCl_3_) *δ* 7.95 (s, 1H, Ar-H), 7.91 (d, *J* = 6.0 Hz, 1H, Ar-H), 7.71 (d, *J* = 9.0 Hz, 2H, Ar-H),7.30–7.24 (m, 7H, Ar-H), 6.92 (d, *J* = 6.0 Hz, 1H, Ar-H), 6.79 (d, *J* = 6.0 Hz, 1H, Ar-H), 6.71 (s, 1H, Ar-H), 6.44 (d, *J* = 6.0 Hz, 1H, Ar-H), 6.23 (s, 1H, Ar-H), 4.78 (brs, 1H, NH), 3.67 (s, 3H, OCH_3_), 3.26 (brs, 2H, NH–CH_2_–), 2.94 (d, *J* = 3.0 Hz, 2H, –CH_2_NHSO_2_), 2.60 (s, 3H, CH_3_), 1.62 (d, *J* = 6.0 Hz, 2H, -CH_2_-); ^13^C NMR (75 MHz, CDCl_3_) *δ* 160.2, 159.3, 147.9, 143.5, 142.5, 140.6, 140.1, 139.9, 137.8, 131.5, 130.4, 130.1, 129.3, 128.1, 127.5, 125.6, 123.2, 120.6, 116.2, 115.3, 112.5, 106.1 (Ar-C), 55.8 (OCH_3_), 40.7 (CH_2_), 38.9 (CH_2_), 30.2 (CH_2_), 22.0 (CH_3_); LC-MS(*m/z*) calculated for C_31_H_31_N_5_O_4_S: 553.21, found: 554.0 (M + 1)^+^.

#### N-(3-((4-(3-(3-Methoxyphenyl)-1-phenyl-1H-pyrazol-4-yl)pyridin-2-yl)amino)propyl)-4-(trifluoromethyl)benzenesulfonamide (2g)

White solid (32%); mp 150–2 °C; ^1^H NMR (300 MHz, CDCl_3_) *δ* 7.84–7.81 (m, 4H, Ar-H), 7.61 (d, *J* = 6.0 Hz, 2H, Ar-H), 7.49 (brs, 1H, NH), 7.21–7.16 (m, 6H, Ar-H), 6.82 (d, *J* = 6.0 Hz, 1H, Ar-H), 6.68 (d, *J* = 5.4, 1H, Ar-H), 6.60 (s, 1H, Ar-H), 6.33 (d, *J* = 3.9 Hz, Ar-H), 6.14 (s, 1H, Ar-H), 4.52 (brs, 1H, NH), 3.56 (s, 3H, OCH_3_), 3.26 (brs, 2H, NH–CH_2_–), 2.89 (brs, 2H, CH_2_NHSO_2_), 1.95 (brs, 2H, -CH_2_-); ^13^C NMR (75 MHz, CDCl_3_) *δ* 159.7, 158.8, 147.1, 142.2, 140.1, 139.5, 139.3, 131.0, 129.9, 128.8, 127.6, 127.4, 126.1, 126.0, 125.1, 122.7, 119.9, 115.7, 114.8, 112.2, 106.1 (Ar-C), 55.2 (OCH_3_), 40.1 (CH_2_), 38.3 (CH_2_), 30.1 (CH_2_); LC-MS(*m/z*) calculated for C_31_H_28_ F_3_N_5_O_3_S: 607.19, found: 608.0 (M + 1)^+^.

#### 3-Fluoro-N-(3-((4-(3-(3-methoxyphenyl)-1-phenyl-1H-pyrazol-4-yl)pyridin-2-yl)amino)propyl)benzenesulfonamide (2h)

White solid (36%); mp 120–2 °C; ^1^H NMR (300 MHz, CDCl_3_) *δ* 7.94 (s, 1H, Ar-H), 7.91 (d, *J* = 6.0 Hz, 1H, Ar-H), 7.30–7.22 (m, 10H, Ar-H), 6.93 (dd, *J* = 2.0 Hz, *J* = 6.0 Hz, 1H, Ar-H), 6.89 (d, *J* = 6.0 Hz, 1H, Ar-H), 6.70 (s, 1H, Ar-H), 6.44 (d, *J* = 6.0 Hz, 1H, Ar-H), 6.24 (s, 1H, Ar-H), 4.74 (s, 1H, NH) 3.67 (s, 3H, OCH_3_), 3.31 (brs, 2H, NH–CH_2_–), 2.98 (t, *J* = 6.0 Hz, 2H, CH_2_NHSO_2_), 1.65 (brs, 2H, –CH_2_–); ^13^C NMR (75 MHz, CDCl_3_) *δ* 159.6, 158.8, 147.2, 142.5, 140.1, 139.5, 139.3, 131.0, 130.8, 130.7, 129.9, 128.8, 127.6, 125.1, 122.7, 122.7, 120.0, 119.5, 119.2, 115.7, 114.8, 114.4, 114.1, 112.1, 105.9 (Ar-C), 55.2 (OCH_3_), 40.1 (CH_2_), 38.2(CH_2_), 29.9 (CH_2_); LC-MS(*m/z*) calculated for C_30_H_28_ FN_5_O_3_S: 557.19, found: 558.0 (M + 1)^+^.

#### N-(3-((4-(3-(3-Methoxyphenyl)-1-phenyl-1H-pyrazol-4-yl)pyridin-2-yl)amino)propyl) naphthalene-1-sulfonamide (2i)

White solid (66%); mp 134–6 °C; ^1^H NMR (300 MHz, CDCl_3_) *δ* 8.40 (s, 1H, Ar-H), 7.97–7.78 (m, 5H, Ar-H), 7.60 (d, *J* = 6.0 Hz, 2H, Ar-H), 730–7.22 (m, 6H, Ar-H), 6.91 (d, *J* = 9.0 Hz, 1H, Ar-H), 6.77 (d, *J* = 6.0 Hz, 1H, Ar-H), 6.70 (s, 1H, Ar-H), 6.45 (d, *J* = 6.0 Hz, 1H, Ar-H), 6.20 (s, 1H, Ar-H), 4.61 (s, 1H, NH) 3.65 (s, 3H, OCH_3_), 3.31 (brs, 2H, NH–CH_2_–), 3.00 (s, 2H, CH_2_NHSO_2_), 1.64 (brs, 2H, –CH_2_–); ^13^C NMR (75 MHz, CDCl_3_) *δ* 159.6, 158.8, 147.5, 142.0, 140.1, 139.6, 139.4, 137.2, 134.6, 132.1, 131.0, 129.9, 129.3, 129.1, 128.8, 128.5, 128.1, 127.8, 127.6, 127.4, 125.1, 122.7, 122.4, 120.0, 115.7, 114.8, 112.1, 105.8 (Ar-C), 55.2 (OCH_3_), 40.1 (CH_2_), 38.2 (CH_2_), 29.9 (CH_2_); LC-MS(*m/z*) calculated for C_30_H_28_ FN_5_O_3_S: 589.21, found: 590.0 (M + 1)^+^.

#### General procedure for synthesis of N-(2-((4-(3-(3-hydroxyphenyl)-1-phenyl-1H-pyrazol-4-yl)pyridin-2-yl)amino)ethyl)benzenesulfonamide (1j), N-(2-((4-(3-(3-hydroxyphenyl)-1-phenyl-1H-pyrazol-4-yl)pyridin-2-yl)amino)ethyl)benzenesulfonamide (1k-q), N-(3-((4-(3-(3-hydroxyphenyl)-1-phenyl-1H-pyrazol-4-yl)pyridin-2-yl)amino)propyl)benzenesulfonamide (2j) and N-(3-((4-(3-(3-hydroxyphenyl)-1-phenyl-1H-pyrazol-4-yl)pyridin-2-yl)amino)propyl) (substituted)benzenesulfonamide (2k-q)

To a mixture of compound (**1a–i**) or (**2a–i**) (0.1 mmol) in methylene chloride (5 mL), BBr_3_ (0.13 g, 1.0 mmol) was added dropwise at –78 °C under nitrogen, and the reaction mixture was stirred at 0 °C for 24 h. The mixture was quenched with saturated aqueous NaHCO_3_. Ethyl acetate (10 mL) was added and the organic layer was separated. The aqueous layer was extracted with ethyl acetate (3 × 10 mL). The combined organic layer extracts were washed with brine and dried over anhydrous Na_2_SO_4_. The organic solvent was evaporated under reduced pressure, and the residue was purified by column chromatography.

#### N-(2-((4-(3-(3-Hydroxyphenyl)-1-phenyl-1H-pyrazol-4-yl)pyridin-2-yl)amino)ethyl) benzenesulfonamide (1j)

Light brown solid (36%); mp 100–2 °C; ^1^H NMR (400 MHz,CD_3_OD) *δ* 8.05 (s, 1H, Ar-H), 7.83 (d, *J* = 7.2 Hz, 2H, Ar-H), 7.76 (d, *J* = 5.6 Hz, 1H, Ar-H), 7.54–7.50 (m, 3H, Ar-H), 7.37–7.34 (m, 3H, Ar-H), 7.29–7.27 (m, 2H, Ar-H), 7.18 (t, *J* = 8.0 Hz, 1H, Ar-H), 6.84–6.81 (m, 1H, Ar-H), 6.67–6.64 (m, 2H, Ar-H), 6.46 (dd, *J* = 5.2 Hz, *J* = 1.2 Hz, 1H, Ar-H), 6.38 (s, 1H, Ar-H), 3.26 (t, *J* = 6.0 Hz, 2H, NH–CH_2_–), 2.99 (t, *J* = 6.0 Hz, 2H, –CH_2_NHSO_2_), ^13^C NMR (100 MHz, CD_3_OD) *δ* 158.6, 157.6, 149.5, 141.9, 140.3, 139.3, 138.9, 132.1, 130.7, 129.7, 128.7, 128.5, 127.7, 126.5, 121.2, 119.7, 116.8, 115.9, 112.3, 105.7 (Ar-C), 42.2 (CH_2_), 40.9 (CH_2_); LC-MS(*m/z*) calculated for C_28_H_25_N_5_O_3_S: 511.17, found: 512.0 (M + 1)^+^.

#### 4-Bromo-N-(2-((4-(3-(3-hydroxyphenyl)-1-phenyl-1H-pyrazol-4-yl)pyridin-2-yl)amino)ethyl) benzenesulfonamide (1k)

Buff solid (30%); mp 178–80 °C; ^1^H NMR (400 MHz,CDCl_3_) *δ* 7.88 (s, 1H, Ar-H), 7.56 (d, *J* = 6.0 Hz, 3H, Ar-H), 7.42 (d, *J* = 8.1 Hz, 2H, Ar-H), 7.17–7.08 (m, 6H, Ar-H), 6.80 (d, *J* = 7.6 Hz, 1H, Ar-H), 6.64 (s, 1H, Ar-H), 6.58 (d, *J* = 7.1 Hz, 1H, Ar-H), 6.37 (d, *J* = 4.7 Hz, 1H, Ar-H), 6.17 (s, 1H, Ar-H), 5.08 (brs, 1H, NH), 3.13 (brs, 2H, NH–CH_2_–), 2.93 (brs, 2H, –CH_2_NHSO_2_); ^13^C NMR (100 MHz, CD_3_OD) *δ* 158.0, 157.3, 146.6, 146.4, 142.4, 140.6, 139.1, 138.7, 132.3, 132.1, 130.7, 129.0, 128.5, 128.3, 127.4, 125.2, 124.9, 119.6, 117.4, 117.2, 116.2, 112.2, 105.4 (Ar-C), 42.9 (CH_2_), 41.5 (CH_2_); LC-MS(*m/z*) calculated for C_28_H_24_BrN_5_O_3_S: 589.17, found: 590.0 (M + 1)^+^.

#### 4-Chloro-N-(2-((4-(3-(3-hydroxyphenyl)-1-phenyl-1H-pyrazol-4-yl)pyridin-2-yl)amino)ethyl) benzenesulfonamide (1l)

Light brown solid (41%); mp 100–2 °C; ^1^H NMR (400 MHz, CD_3_OD) *δ* 8.05 (s, 1H, Ar-H), 7.78–7.74 (m, 3H, Ar-H), 7.47 (d, *J* = 8.8 Hz, 2H, Ar-H), 7.35–7.32 (m 3H, Ar-H), 7.28–7.27 (m, 2H, Ar-H), 7.18 (t, *J* = 8.0 Hz, 1H, Ar-H), 6.84–6.81 (m, 1H, Ar-H), 6.67 (s, 1H, Ar-H), 6.66 (t, *J* = 2.0 Hz, 1H, Ar-H), 6.47 (dd, *J* = 5.2 Hz, *J* = 1.2 Hz, 1H, Ar-H), 6.34 (s, 1H, Ar-H), 3.25 (t, *J* = 6.0 Hz, 2H, NH–CH_2_–), 3.01 (t, *J* = 6.0 Hz, 2H, –CH_2_NHSO_2_), ^13^C NMR (100 MHz, CD_3_OD) *δ* 158.5, 157.6, 146.5, 1141.9, 14.0.9, 139.3, 139.1, 138.9, 138.2, 130.7, 129.8, 129.2, 129.0, 128.9, 128.5, 128.2, 127.7, 126.2, 121.2, 119.7, 116.9, 115.9, 111.3, 105.6 (Ar-C), 42.2 (CH_2_), 40.8(CH_2_); LC-MS(*m/z*) calculated for C_28_H_24_ClN_5_O_3_S: 545.13, found: 546.0 (M + 1)^+^.

#### 4-Fluoro-N-(2-((4-(3-(3-hydroxyphenyl)-1-phenyl-1H-pyrazol-4-yl)pyridin-2-yl)amino)ethyl) benzenesulfonamide (1m)

Light yellow solid (40.5%); mp 106–8 °C; ^1^H NMR (400 MHz, CDCl_3_) *δ* 7.90 (s, 1H, Ar-H), 7.78–7.75 (m, 2H, Ar-H), 7.60 (d, *J* = 8.0 Hz, 1H, Ar-H), 7.20 (s, 5H, Ar-H), 7.12 (t, *J* = 8.0 Hz, 1H, Ar-H), 7.03 (t, *J* = 8.0 Hz, 2H, Ar-H), 6.83 (d, *J* = 8.0 Hz, 1H, Ar-H), 6.67 (s, 1H, Ar-H), 6.61 (d, *J* = 8.0 Hz, 1H, Ar-H), 6.40 (d, *J* = 8.0 Hz, 1H, Ar-H), 6.22 (s, 1H, Ar-H) 3.18 (s, 2H, NH–CH_2_–), 2.97 (s, 2H, –CH_2_NHSO_2_), ^13^C NMR (100 MHz, CDCl_3_) *δ* 166.1, 163.6, 158.1, 157.3, 146.6, 142.4, 140.6, 139.2, 135.7, 130.7, 130.2, 129.7, 129.6, 128.8, 127.8, 125.1, 121.7, 119.6, 116.7, 116.3, 116.1, 112.3, 105.5 (Ar-C), 42.9 (CH_2_), 41.6 (CH_2_); LC-MS(*m/z*) calculated for C_28_H_24_FN_5_O_3_S: 529.59, found: 530.0 (M + 1)^+^.

#### N-(2-((4-(3-(3-Hydroxyphenyl)-1-phenyl-1H-pyrazol-4-yl)pyridin-2-yl)amino)ethyl)-4-methylbenzenesulfonamide (1n)

Yellow solid (38%); mp 114–6 °C; ^1^H NMR (400 MHz, CDCl_3_) *δ* 7.91 (s, 1H, Ar-H), 7.69 (d, *J* = 8.0 Hz, 3H, Ar-H), 7.29–7.21 (m, 6H, Ar-H), 7.15 (t, *J* = 8.0 Hz, 1H, Ar-H), 6.87 (d, *J* = 8.0 Hz, 1H, Ar-H), 6.72 (s, 1H, Ar-H), 6.61 (d, *J* = 8.0 Hz, 1H, Ar-H), 6.44 (s, 1H, Ar-H), 6.22 (s, 1H, Ar-H), 5.32 (brs, 1H, NH), 3.20 (brs, 2H, NH–CH_2_–), 3.00–2.98 (m, 2H, –CH_2_NHSO_2_), 2.78 (s, 3H, CH_3_); ^13^C NMR (100 MHz, CDCl_3_) *δ* 158.2, 157.1, 146.7, 143.4, 142.4, 140.4, 139.3, 139.2, 136.7, 130.8, 130.2, 129.7, 128.8, 127.6, 127.0, 125.0, 121.8, 119.7, 116.8, 112.3, 105.6 (Ar-C), 42.9 (CH_2_), 41.6 (CH_2_), 21.0(CH_3_); LC-MS(*m/z*) calculated for C_29_H_29_N_5_O_3_S: 525.59, found: 526.0 (M + 1)^+^.

#### N-(2-((4-(3-(3-Hydroxyphenyl)-1-phenyl-1H-pyrazol-4-yl)pyridin-2-yl)amino)ethyl)-4-(trifluoromethyl)benzenesulfonamide (1o)

White solid (40%); mp 138–40 °C; ^1^H NMR (400 MHz, CDCl_3_) *δ* 7.86 (d, *J* = 9.0 Hz, 3H, Ar-H), 7.62–7.57 (m, 3H, Ar-H), 7.17 (s, 5H, Ar-H), 7.10 (t, *J* = 7.6 Hz, 1H, Ar-H), 6.80 (d, *J* = 7.1 Hz, 1H, Ar-H), 6.64 (s, 1H, Ar-H), 6.58 (d, *J* = 7.0 Hz, 1H, Ar-H), 6.38 (s, 1H, Ar-H), 5.32 (brs, 1H, NH), 3.20 (brs, 2H, NH–CH_2_–), 3.00–2.98 (m, 2H, –CH_2_NHSO_2_), 2.78 (s, 3H, CH_3_); LC-MS(*m/z*) calculated for C_29_H_24_F_3_N_5_O_3_S: 579.59, found: 580.0 (M + 1)^+^.

#### 3-Fluoro-N-(2-((4-(3-(3-hydroxyphenyl)-1-phenyl-1H-pyrazol-4-yl)pyridin-2-yl)amino) ethyl)benzenesulfonamide (1p)

White solid (32%); mp 146–8 °C; ^1^H NMR (400 MHz, CD_3_OD) *δ* 8.05 (s, 1H, Ar-H), 7.87 (d, *J* = 5.2 Hz, 1H, Ar-H), 7.64 (d, *J* = 7.6 Hz, 1H, Ar-H), 7.56–7.51 (m, 2H, Ar-H), 7.37–7.28 (m, 6H, Ar-H), 7.19 (t, *J* = 8.0 Hz, 1H, Ar-H), 6.82 (d, *J* = 8.0 Hz, 1H, Ar-H), 6.68–6.65 (m, 2H, Ar-H), 6.47 (d, *J* = 5.6 Hz, 1H, Ar-H), 6.37 (s, 1H, Ar-H), 3.27 (t, *J* = 5.2 Hz, 2H, NH–CH_2_–), 3.02 (t, *J* = 6.0 Hz, 2H, -CH_2_NHSO_2_); ^13^C NMR (75 MHz, CD_3_OD) *δ* 163.6, 161.1, 158.6, 157.6, 146.6, 142.6, 141.9, 139.3, 138.9, 130.8, 130.0, 130.7, 129.7, 128.5, 127.7, 125.2, 122.5, 121.1, 119.7, 119.1, 118.8, 116.8, 113.7, 113.5, 111.3, 105.7 (Ar-C), 42.2 (CH_2_), 40.8 (CH_2_); LC-MS(*m/z*) calculated for C_28_H_24_ FN_5_O_3_S: 529.16, found: 530.0 (M + 1)^+^.

#### N-(2-((4-(3-(3-Hydroxyphenyl)-1-phenyl-1H-pyrazol-4-yl)pyridin-2-yl)amino)ethyl)naphthalene-1-sulfonamide (1q)

White solid (33%); mp 146–8 °C; ^1^H NMR (400 MHz, CDCl_3_) *δ* 8.37 (s, 1H, Ar-H), 7.87–7.73 (m, 5H, Ar-H), 7.61–7.51 (m, 3H, Ar-H), 7.22 (d, *J* = 4.0 Hz, 5H, Ar-H), 7.11 (t, *J* = 8.0 Hz, 1H, Ar-H), 6.84 (dd, *J* = 8.0 Hz, *J* = 4.0 Hz, 1H, Ar-H), 6.69 (t, *J* = 4.0 Hz, 1H, Ar-H), 6.58 (d, *J* = 8.0 Hz, 1H, Ar-H), 6.38 (dd, *J* = 8.0 Hz, *J* = 4.0 Hz, 1H, Ar-H), 6.15 (s, 1H, Ar-H), 5.03 (brs, 1H, NH), 3.19 (brs, 2H, NH–CH_2_–), 3.02 (t, *J* = 8.0 Hz, 2H, –CH_2_NHSO_2_); ^13^C NMR (75 MHz, CDCl_3_) *δ* 158.0, 157.2, 146.5, 142.4, 140.5, 139.2, 136.4, 134.7, 132.0, 130.7, 130.2, 129.4, 129.2, 128.8, 128.3, 127.8, 127.7, 127.4, 125.0, 122.1, 121.7, 119.6, 117.3, 116.8, 112.3, 105.5 (Ar-C), 43.0 (CH_2_), 41.6 (CH_2_); LC-MS(*m/z*) calculated for C_32_H_27_N_5_O_3_S: 561.66, found: 562.0 (M + 1)^+^.

#### N-(3-((4-(3-(3-Hydroxyphenyl)-1-phenyl-1H-pyrazol-4-yl)pyridin-2-yl)amino)propyl) benzenesulfonamide (2j)

White solid (37%); mp 154–6 °C; ^1^H NMR (400 MHz, CDCl_3_) *δ* 7.92 (s, 1H, Ar-H), 7.81–7.74 (m, 3H, Ar-H), 7.51 (d, *J* = 7.2 Hz, 1H, Ar-H), 7.44 (t, *J* = 7.6 Hz, 2H, Ar-H), 7.26 (s, 5H, Ar-H), 7.14 (t, *J* = 7.9 Hz, 1H, Ar-H), 6.87 (d, *J* = 7.88 Hz, 1H, Ar-H), 6.74 (s, 1H, Ar-H), 6.60 (d, *J* = 7.4 Hz, 1H, Ar-H), 6.51 (d, *J* = 5.3 Hz, 1H, Ar-H), 6.11(s, 1H, Ar-H), 4.83 (brs, 1H, NH), 3.06 (brs, 2H, NH–CH_2_–), 2.88 (d, *J* = 5.8 Hz, 2H, –CH_2_NHSO_2_), 1.53 (p, *J* = 5.8 Hz, 2H, CH_2_CH_2_CH_2_); LC-MS(*m/z*) calculated for C_29_H_27_N_5_O_3_S: 525.63, found: 526.0 (M + 1)^+^.

#### 4-Bromo-N-(3-((4-(3-(3-hydroxyphenyl)-1-phenyl-1H-pyrazol-4-yl)pyridin-2-yl)amino)propyl)benzenesulfonamide (2k)

Light yellow solid (42%); mp 184–6 °C; ^1^H NMR (400 MHz, CD_3_OD) *δ* 8.06 (s, 1H, Ar-H), 7.79 (d, *J* = 5.2 Hz, 1H, Ar-H), 7.80–7.67 (m, 4H, Ar-H), 7.37–7.34 (m, 3H, Ar-H), 7.29–7.27 (m, 2H, Ar-H), 7.19 (t, *J* = 8.4 Hz, 1H, Ar-H), 6.84–6.81 (m, 1H, Ar-H), 6.68 (s, 1H, Ar-H), 6.66 (s, 1H, Ar-H), 6.51 (dd, *J* = 5.6 Hz, *J* = 1.2 Hz, 1H, Ar-H), 6.34 (s, 1H, Ar-H), 3.14 (t, *J* = 8.0 Hz, 2H, NH–CH_2_–), 2.93 (t, *J* = 8.0 Hz, 2H, –CH_2_NHSO_2_); 1.61 (d, *J* = 6.0 Hz, 2H, CH_2_CH_2_CH_2_); ^13^C NMR (100 MHz, CD_3_OD) *δ* 158.9, 157.6, 146.6, 141.9, 140.9, 139.7, 139.3, 138.9, 132.0, 130.8, 129.8, 128.5, 128.3, 127.7, 126.6, 125.2, 121.2, 119.8, 116.8, 115.9, 110.9, 105.4 (Ar-C), 40.2 (CH_2_), 38.1 (CH_2_), 28.8 (CH_2_); LC-MS(*m/z*) calculated for C_29_H_26_BrN_5_O_3_S: 604.10, found: 605.0 (M + 1)^+^.

#### 4-Chloro-N-(3-((4-(3-(3-hydroxyphenyl)-1-phenyl-1H-pyrazol-4-yl)pyridin-2-yl)amino)propyl) benzenesulfonamide (2l)

White solid (52%); mp 166–8 °C; ^1^H NMR (400 MHz, CD_3_OD) *δ* 8.06 (s, 1H, Ar-H), 7.87–7.75 (m, 3H, Ar-H), 7.60–7.46 (m, 2H, Ar-H), 7.36 (t, *J* = 5.8 Hz, 3H, Ar-H), 7.32–7.26 (m, 2H, Ar-H), 7.19 (t, *J* = 7.9 Hz, 1H, Ar-H), 6.83 (ddd, *J* = 8.3 Hz, 2.3 Hz, 0.8 Hz, 1H, Ar-H), 6.67 (t, *J* = 5.0 Hz, 2H, Ar-H), 6.51 (dd, *J* = 5.5 Hz, 1.4 Hz, 1H, Ar-H), 6.34 (s, 1H, Ar-H), 3.14 (t, *J* = 6.6 Hz, 2H, NH–CH_2_-), 2.93 (t, *J* = 6.8 Hz, 2H, –CH_2_NHSO_2_), 1.61 (p, *J* = 6.7 Hz, 2H, CH_2_CH_2_CH_2_); ^13^C NMR (100 MHz, CD_3_OD) *δ* 158.9, 157.6, 146.7, 141.9, 140.9, 139.3, 139.2, 138.9, 138.3, 130.8, 129.8, 129.0, 128.5, 128.2, 127.7, 125.2, 121.2, 119.8, 116.8, 115.9, 110.9, 105.4 (Ar-C), 40.2 (CH_2_), 38.1 (CH_2_), 28.8 (CH_2_); LC-MS(*m/z*) calculated for C_29_H_26_ClN_5_O_3_S: 560.7, found: 561.0 (M + 1)^+^.

#### 4-Fluoro-N-(3-((4-(3-(3-hydroxyphenyl)-1-phenyl-1H-pyrazol-4-yl)pyridin-2-yl)amino)propyl)benzenesulfonamide (2m)

White solid (45%); mp 144–6 °C; ^1^H NMR (400 MHz, CD_3_OD) *δ* 8.06 (s, 1H, Ar-H), 7.98–7.86 (m, 2H, Ar-H), 7.80 (d, *J* = 5.5 Hz, 1H, Ar-H), 7.37 (d, *J* = 6.6 Hz, 3H, Ar-H), 7.33–7.24 (m, 4H, Ar-H), 7.20 (t, *J* = 7.8 Hz, 1H, Ar-H), 6.83 (d, *J* = 8.2 Hz, 1H, Ar-H), 6.67 (d, *J* = 8.4 Hz, 2H, Ar-H), 6.51 (d, *J* = 5.5 Hz, 1H, Ar-H), 6.35 (s, 1H, Ar-H), 3.15 (t, *J* = 6.6 Hz, 2H, NH–CH_2_–), 2.92 (t, *J* = 6.7 Hz, 2H, –CH_2_NHSO_2_), 1.74–1.53 (m, 2H, CH_2_CH_2_CH_2_); ^13^C NMR (100 MHz, CD_3_OD) *δ* 158.9, 157.6, 146.7, 141.9, 139.3, 138.8, 136.7, 130.8, 129.7, 129.5, 129.4, 128.5, 127.7, 125.2, 121.2, 119.8, 116.8, 115.9, 115.8, 115.6, 110.9, 105.4 (Ar-C), 40.2 (CH_2_), 38.2 (CH_2_), 28.8 (CH_2_); LC-MS(*m/z*) calculated for C_29_H_26_FN_5_O_3_S: 543.17, found: 544.0 (M + 1)^+^.

#### N-(3-((4-(3-(3-Hydroxyphenyl)-1-phenyl-1H-pyrazol-4-yl)pyridin-2-yl)amino)propyl)-4-methylbenzenesulfonamide (2n)

White solid (33%); mp 158–60 °C; ^1^H NMR (400 MHz, CD_3_OD) *δ* 8.06 (s, 1H, Ar-H), 7.79 (d, *J* = 5.5 Hz, 1H, Ar-H), 7.72 (d, *J* = 8.3 Hz, 2H, Ar-H), 7.41–7.25 (m, 7H, Ar-H), 7.19 (t, *J* = 7.9 Hz, 1H, Ar-H), 6.83 (ddd, *J* = 8.3 Hz, 2.3 Hz, 1.0 Hz, 1H, Ar-H), 6.71–6.61 (m, 2H, Ar-H), 6.51 (dd, *J* = 5.5, 1.5 Hz, 1H, Ar-H), 6.34 (s, 1H, Ar-H), 3.13 (t, *J* = 6.7 Hz, 2H, NH–CH_2_–), 2.89 (t, *J* = 6.8 Hz, 2H,–CH_2_NHSO_2_), 2.41 (s, 3H, CH_3_), 1.60 (p, *J* = 6.7 Hz, 2H, CH_2_CH_2_CH_2_); ^13^C NMR (100 MHz, CD_3_OD) *δ* 158.9, 157.6, 146.7, 143.1, 141.9, 140.9, 139.3, 138.9, 137.4, 130.8, 129.8, 129.3, 128.5, 127.7, 126.6, 125.2, 121.2, 119.8, 116.8, 115.9, 110.9, 105.4 (Ar-C), 40.2 (CH_2_), 38.2 (CH_2_), 28.9 (CH_2_), 20.1 (CH_3_); LC-MS(*m/z*) calculated for C_30_H_29_N_5_O_3_S: 539.20, found: 540.0 (M + 1)^+^.

#### N-(3-((4-(3-(3-Hydroxyphenyl)-1-phenyl-1H-pyrazol-4-yl)pyridin-2-yl)amino)propyl)-4-(trifluoromethyl)benzenesulfonamide (2o)

White solid (39%); mp 150–2 °C; ^1^H NMR (400 MHz, CD_3_OD) *δ* 8.09–7.99 (m, 3H, Ar-H), 7.87 (d, *J* = 8.3 Hz, 2H, Ar-H), 7.80 (d, *J* = 5.5 Hz, 1H, Ar-H), 7.41–7.34 (m, 3H, Ar-H), 7.34–7.25 (m, 2H, Ar-H), 7.20 (t, *J* = 7.9 Hz, 1H, Ar-H), 6.83 (ddd, *J* = 8.3 Hz, 2.4 Hz, 0.9 Hz, 1H, Ar-H), 6.71–6.63 (m, 2H, Ar-H), 6.52 (dd, *J* = 5.6 Hz, 1.5 Hz, 1H, Ar-H), 6.36 (d, *J* = 6.0 Hz, 1H, Ar-H), 3.16 (t, *J* = 6.7 Hz, 2H, NH–CH_2_–), 2.96 (t, *J* = 6.8 Hz, 2H, –CH_2_NHSO_2_), 1.64 (p, *J* = 6.7 Hz, 2H, CH_2_CH_2_CH_2_); ^13^C NMR (100 MHz, CD_3_OD) *δ* 158.8, 157.6, 146.5, 142.0, 140.9, 139.3, 138.8, 130.7, 129.7, 128.5, 127.7, 127.3, 125.9, 125.2, 121.2, 119.7, 116.8, 115.9, 110.9, 105.5, (Ar-H), 40.2 (CH_2_), 38.1 (CH_2_), 28.9 (CH_2_); LC-MS(*m/z*) calculated for C_30_H_26_F_3_N_5_O_3_S: 593.20 found: 594.0 (M + 1)^+^.

#### 3-Fluoro-N-(3-((4-(3-(3-hydroxyphenyl)-1-phenyl-1H-pyrazol-4-yl)pyridin-2-yl)amino) propyl) benzenesulfonamide (2p)

White solid (41%); mp 120–2 °C; ^1^H NMR (400 MHz, CD_3_OD) *δ* 8.05 (s, 1H, Ar-H), 7.79 (d, *J* = 5.5 Hz, 1H, Ar-H), 7.67 (d, *J* = 7.9 Hz, 1H, Ar-H), 7.63–7.51 (m, 2H, Ar-H), 7.41–7.25 (m, 6H, Ar-H), 7.19 (t, *J* = 7.9 Hz, 1H, Ar-H), 6.87–6.79 (m, 1H, Ar-H), 6.67 (t, *J* = 4.2 Hz, 2H, Ar-H), 6.50 (dd, *J* = 5.5 Hz, 1.3 Hz, 1H, Ar-H), 6.36 (s, 1H, Ar-H), 3.15 (t, *J* = 6.7 Hz, 2H, NH–CH_2_–), 2.93 (t, *J* = 6.8 Hz, 2H, –CH_2_NHSO_2_) , 1.63 (p, *J* = 6.7 Hz, 2H, CH_2_CH_2_CH_2_), ^13^C NMR (100 MHz, CD_3_OD) *δ* 163.7, 161.2, 158.8, 157.6, 146.5, 142.7, 141.9, 140.9, 139.3, 138.9, 130.9, 130.8, 130.8, 129.8, 128.5, 127.7, 125.2, 122.5, 122.5, 121.2, 119.8, 119.1, 118.9, 116.8, 115.9, 113.7, 113.5, 110.9, 105.5 (Ar-C), 40.2 (CH_2_), 38.2 (CH_2_), 28.9 (CH_2_); LC-MS(*m/z*) calculated for C_29_H_26_FN_5_O_3_S: 543.17, found: 544.0 (M + 1)^+^.

#### N-(3-((4-(3-(3-Hydroxyphenyl)-1-phenyl-1H-pyrazol-4-yl)pyridin-2-yl)amino)propyl) naphthalene-1-sulfonamide (2q)

White solid (40%); mp 168–70 °C; ^1^H NMR (400 MHz, CD_3_OD) *δ* 8.40 (s, 1H, Ar-H), 8.01 (s, 1H, Ar-H), 7.97 (d, *J* = 8.4 Hz, 2H, Ar-H), 7.92 (d, *J* = 8.0 Hz, 1H, Ar-H), 7.81 (dd, *J* = 8.8 Hz, 1.6 Hz, 1H, Ar-H), 7.73 (d, *J* = 5.2 Hz, 1H, Ar-H), 7.65–7.57 (m, 2H, Ar-H), 7.36–7.25 (m, 5H, Ar-H), 7.13 (t, *J* = 8.0 Hz, 1H, Ar-H), 6.79 (dd, *J* = 8.4 Hz, 2.0 Hz, 1H, Ar-H), 6.62 (s, 1H, Ar-H), 6.59 (d, *J* = 6.4 Hz, 1H, Ar-H), 6.46 (d, *J* = 4.8 Hz, 1H, Ar-H), 6.27 (s, 1H, Ar-H), 3.11 (t, *J* = 6.8 Hz, 2H), 2.94 (t, *J* = 6.8 Hz, 2H, Ar-H), 1.60 (t, *J* = 6.8 Hz, 2H, Ar-H); ^13^C NMR (100 MHz, CD_3_OD) *δ* 158.7, 157.6, 146.4, 141.9, 140.9, 139.3, 138.9, 137.2, 134.7, 132.1, 130.7, 129.7, 129.1, 128.8, 128.5, 128.3, 127.7, 127.6, 127.5, 127.2, 125.2, 122.0, 121.2, 119.7, 116.8, 115.9, 110.9, 105.3 (Ar-C), 40.2 (CH_2_), 38.2 (CH_2_), 28.8 (CH_2_); LC-MS(*m/z*) calculated for C_33_H_29_N_5_O_3_S: 575.20 found: 576.0 (M + 1)^+^.

### Antiproliferative screening of the target compounds against NCI-55 cancer cell line panel

Screening against the cancer cell lines was carried out at the National Cancer Institute (NCI, Bethesda, Maryland, USA) applying the standard protocol of the NCI^22^.

### *In vitro* kinase screening

To investigate the biological target of compound, Reaction Biology Corp. Kinase HotSpotSM service was used adopting standard assay protocol^23^.

### Plasma stability

About 10 µM of compound **2l** was mixed with human plasma and rat plasma and the mixture was shaked at 37 °C for 30 min and 120 min. After each time interval, acetonitrile was added and the mixture was centrifugated (14000 rpm, 4 °C). The supernatant was injected into the LC-MS to detect the remaining amount of compound **2l**. 

### hERG binding assay

hERG binding assay was performed using hERG Fluorescence Polarization Assay (Invitrogen: PV5365) assay kit and Synergy Neo (Biotek) and standard procedures were applied.

### In vivo pharmacokinetic assay

Adult male rats (*N* = 3/group) were administered with compound **2l** dissolved in distilled water (tween 80) at a single dose of 10 mg/kg by oral administration and 10 mg/mL by injection. Serum samples were collected each 30 min. The blood concentration of the test compounds was determined by LC-MS/MS (Agilent 1290 infinity II series equipped with on-line degasser, binary pump, thermostatted well-plate autosampler and column compartment, Waters Atlantis® HSS T3 (2.1 × 100 mm, 1.9 µm) column, and mobile phase linear gradient from 95% A (0.1% formic acid in water) /5% B (0.1% formic acid in acetonitrile) to 5% A/95% B, 6500+ QTRAP LC-MS/MS/MS system, Turbo Spray Ion Drive as ion source, and Carbamazepine as internal standard. Pharmacokinetic parameters were obtained by non-compartmental analysis of the plasma concentration–time profiles using KineticaTM 4.4.1 (Thermo Fisher Scientific, Inc., Woburn, MA, USA).

## Result and discussion

### Chemistry

**Scheme 1.**
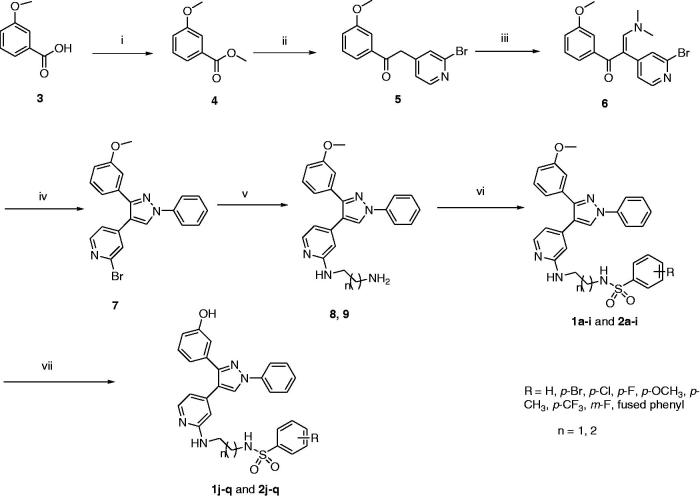
Synthetic pathway for final target compounds **1a–q** and **2a–q**. Reagents and conditions: (i) H_2_SO_4_, CH_3_OH, reflux, 8 h; (ii) 2-bromo-4-methylpyridine, LiHMDS, THF, –25 °C to rt, overnight; (iii) DMF-DMA, reflux, 18 h; (iv) phenylhydrazine, C_2_H_5_OH, rt, overnight; (v) 1,2-ethylenediamine or 1,3-propylenediamine, reflux, 8 h.; (vi) appropriate sulfonyl chloride, Et_3_N, CH_2_Cl_2_, 0 °C, overnight; (vii) BBr_3_, CH_2_Cl_2_, –78 °C; 0 °C, overnight.

**Scheme 2.**
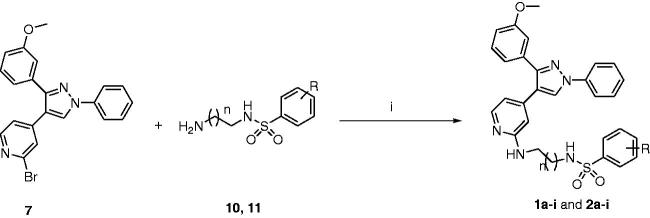
Alternative pathway for synthesis of compounds **1a–i** and **2a–i**. Reagents and conditions: (i) pyridine, 110 °C, overnight.

Synthesis of the final compounds was achieved using the pathway illustrated in Scheme 1. Esterification of 3-methoxybenzoic acid (**3**) using methanol and Conc. sulphuric acid produced methyl 3-methoxybenzoate (**4**). Reaction of **4** with 2-bromo-4-methylpyridine in the presence of LiHMDS led to formation of 2-(2-bromopyridin-4-yl)-1-(3-methoxyphenyl)ethan-1-one (**5**). Compound **5** was refluxed with DMF-DMA to give (*Z*)-2-(2-bromopyridin-4-yl)-3-(dimethylamino)-1-(3-methoxyphenyl)prop-2-en-1-one (**6**), which further reacts with phenylhydrazine produced 2-bromo-4-(3-(3-methoxyphenyl)-1-phenyl-1*H*-pyrazol-4-yl)pyridine (**7**). Compound **7** reacted with ethylenediamine or 1,3-propylenediamine to produce *N^1^*-(4-(3-(3-methoxyphenyl)-1-phenyl-1*H*-pyrazol-4-yl)pyridin-2-yl)ethane-1,2-diamine (**8**) and *N^1^*-(4-(3-(3-methoxyphenyl)-1-phenyl-1*H*-pyrazol-4-yl)pyridin-2-yl)propane-1,3-diamine (**9**). Reaction of compound **8** or **9** with the appropriate arylsulfonyl chloride in the presence of Et_3_N afforded the first group of final compounds **1a–i** and **2a–i** which bears the *m*-methoxyphenyl group at position 3 of the pyrazole ring. Demethylation of compounds **1a–i** and **2a–i** using boron tribromide produced corresponding hydroxyl derivatives **1j–q** and **2j–q**. An alternative pathway was investigated to synthesize the final compounds starting from compound **7**. Arylation of *N*-(2-aminoethyl)benzenesulfonamide (**10**) or *N*-(3-aminopropyl)benzenesulfonamide (**11**) with compound **7** in the presence of pyridine at 110 °C led to formation of compounds **1a–i** and **2a–i** (Scheme 2).

### Biology

#### Antiproliferative activity of the target compounds

##### Single-dose testing

The final target compounds were submitted to the National Cancer Institute (NCI), Maryland, USA^23^. Thirty-one compounds were selected to be tested over 60 cell lines at single dose 10 µM. The mean per cent inhibition of the tested compounds are presented in Tables 1 and 2.

**Table 1.** Structures of compounds **1a–i** and **2a–i** and their mean inhibition percentages in single-dose (10 μM) 60-cancer cell line screening.


Compound	n	Ar	Mean % inhibition


**Table 2.** Structures of compounds **1j–q** and **2j–q** and their mean inhibition percentages in single-dose (10 μM) 60-cancer cell line screening.


Compound	n	Ar	Mean % inhibition


The results in Table 1 represent the activity of methoxy compounds **1a–i** and **2a–i**. In general, the methoxy compounds have moderate activity over the cell line panel with the highest activity for compound bearing *p-*trifluoromethyl group and ethylene bridge **1g** with per cent inhibition 92.80% followed by compound **2h** with *m-*fluorobenzensulfonamide moiety and propylene linker and compound **2g** having *p-*trifluoromethyl group and propylene spacer and mean inhibition percentages 76.81% and 76.53%, respectively. Compounds with ethylene bridge and electron-withdrawing group and moderate size such as the chloro derivative **1c** possess mean per cent inhibition higher compared to one with electron-donating group (**1e** and **1f**) and large electron-withdrawing group such as bromo derivative **1b**. Regarding compounds with propylene bridge, compound without any substituents **2a** was more active than corresponding one with both elctron withdrawing groups and electron donating one with exception of *p-*trifluoromethyl and *m-*fluoro (compounds **2g** and **2h**).

On the other side, all demethylated compounds **1j–p** and **2j–p** showed more than 50% per cent inhibition (Table 2). Compounds containing electron-withdrawing group at *para* position such as bromo (**1k** and **2k**), chloro (**1l** and **2l**), and fluoro (**1 m** and **2m**) showed the highest per cent inhibition. Compounds with propylene bridge are more potent compared to compounds having ethylene bridge. The propylene bridge might be suitable for optimum fitting at the receptor site. The most potent compounds in this series were **2 l** with mean per cent inhibition 97.80%, and **2k** with mean per cent inhibition 90.64%. Target compounds with ethylene bridge and electron-withdrawing groups (**1k–m**) were equipotent to **2m** with propylene bridge and *p*-fluoro electron-withdrawing group. The *meta* substitution at sulfonamide moiety was less active than *para* substitution, but more active than methoxy series (**1a–i** and **2a–1**).

**Figure 2.**
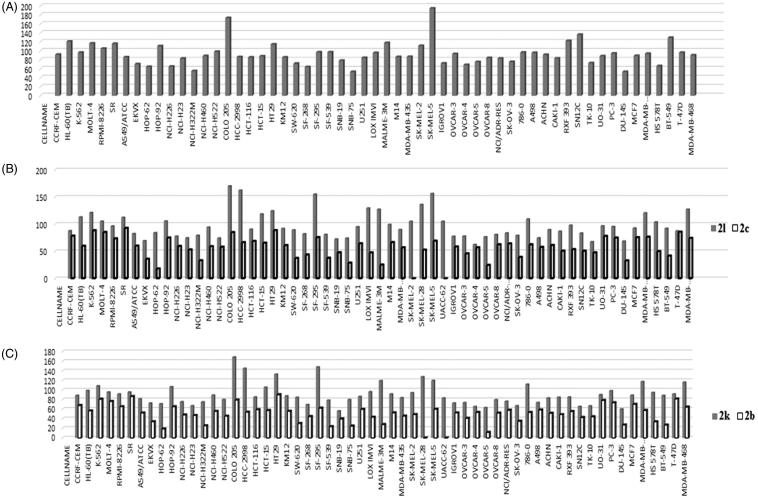
(A) Per cent inhibition of compound **2g** over all cancer cell lines of the NCI panel; (B) per cent inhibition of compound **2c** and **2l** over all cancer cell lines of the NCI panel; (C) mean per cent inhibition of compound **2b** and **2k** over all cancer cell lines of the NCI panel. The compounds were tested at 10 µM concentration.

The detailed inhibitory effects of the most potent hydroxyl compounds **2k** and **2l** and their corresponding methoxy analogues **2b** and **2c**, and the most potent methoxy compound **2g** against NCI-60 cell line panel are depicted in Figure 2. Compound **2g** exhibited more than 100% inhibition over 13 cell lines and showed lethal effect on two cell lines belonging to colon cancer (Colo 205 and HT29) and melanoma (SK-MEL-5). Regarding compounds **2 b** and **2k**, the cellular activity of compound **2k** is much greater than **2b.** The maximum inhibition percentage of compound **2b** was 83.30% over T-47D cell line, while compound **2k** showed per cent inhibition over 100% against 13 cell lines and maximum inhibitions against Colo 205 (168%), HCC-116 (140%) [colon cancer cell lines], and SF-295 (147%) [CNS cancer cell line]. Compound **2c** showed maximum activity 92.74% against SR leukaemia cell line and 88.66% against HT29 colon cancer cell line. Finally, compound **2l** exhibited more than 100% against 24 cell lines and maximum activity against Colo 205, HCC-2998 [colon cancer cell lines], SF-295 [CNS cancer cell line], LOX IMVI, SK-MEL-28, and SK-MEL-5 [melanoma cell lines] with per cent inhibition 170.28%, 162.39%, 155.19%, 129.50%, 136.19%, and 156.35, respectively.

##### Five-dose results

Compounds **1c, 1g, 1k–m, 1o, 2g, 2 h, 2k–m, 2o**, and **2q** with the highest activity in single-dose tests were selected to be tested in a five-dose testing mode to determine their IC_50_, TGI, and LD_50_. The mean IC_50_ of the selected compounds over different cancer subtypes are shown in Table 3. Compounds **1k, 2k, 2l, 2o,** and **2q** were more potent against leukaemia cell lines than sorafenib with mean IC_50_ ranging from 1.44 µM to 2.11 µM. Regarding non-small cell lung cancer, compound **2k** was the most potent compound with IC_50_ 1.96 µM. Compounds **1k, 1m**, **2k, 2l** and **2o** were more potent against colon cancer and CNS cancer cell line with IC_50_ 1.48 µM for **2k** against colon cancer and IC_50_ 1.65 µM against CNS cell lines for **2l**. All tested compounds were more active than sorafenib with IC_50_ 1.62 µM for compound **2k**. Compound **2l** was the most potent compound against ovarian cancer with mean IC_50_ of 2.09 µM. Compound **2k** was the most potent compound against renal cancer cell lines, prostate cancer cell lines, and breast cancer cell lines with mean IC_50_ 1.89, 2.03, and 1.11 µM, respectively.

**Table 3.** Mean ICs_50_ of the most potent compounds and sorafenib over different cancer subtypes.

		Subpanel cancer cell lines^a^								
		I	II	III	IV	V	VI	VII	VIII	IX
**Compound No.**	**1c**	2.89	6.02	4.08	6.33	**4.58**	5.23	4.79	4.99	3.79
	**1g**	3.35	6.36	4.91	5.28	**4.94**	6.10	5.32	13.03	4.05
	**1k**	**2.11**	2.69	**2.29**	**2.18**	**2.03**	2.87	2.48	2.54	2.34
	**1l**	2.44	3.16	2.46	2.85	**2.38**	3.29	3.18	3.03	2.48
	**1m**	2.29	2.65	**2.34**	**2.51**	**2.06**	2.68	2.88	**2.88**	2.16
	**1o**	2.34	3.03	2.38	2.67	**2.37**	3.12	2.45	3.45	2.11
	**2g**	3.35	6.36	4.91	5.28	**4.94**	6.10	5.32	13.03	4.05
	**2h**	3.18	5.01	4.69	4.49	**4.14**	3.88	4.25	4.65	3.49
	**2k**	**1.55**	**1.96**	**1.48**	**1.70**	**1.62**	**2.11**	**1.89**	**2.03**	**1.11**
	**2l**	**1.44**	2.32	**1.51**	**1.65**	**1.68**	**2.09**	**2.06**	**2.28**	**1.58**
	**2m**	2.25	3.91	2.68	3.28	**2.89**	3.79	3.81	3.62	2.69
	**2o**	**1.90**	2.12	**1.64**	**1.71**	**1.72**	2.32	**2.03**	**2.79**	**1.87**
	**2q**	**1.96**	3.05	2.62	**2.33**	**2.27**	5.28	2.99	3.44	**1.95**
**Sorafenib**		2.23	2.11	2.35	2.51	8.57	2.77	2.89	3.16	2.15

Mean IC_50_ values were calculated by dividing the summation of IC_50_ values of the compound over cell lines of the same cancer type by the number of cell lines in the subpanel.

The bold values indicate more potent compared to the reference standard compound.

a I: Leukaemia; II: non-small cell lung cancer; III: colon cancer; IV: CNS cancer; V: melanoma; VI: ovarian cancer; VII: renal cancer; VIII: prostate cancer; IX: breast cancer.

The activity of the most potent compounds **1c**, **1g**, **1k–m**, **1o**, **2g**, **2h**, **2k–m**, **2o**, and **2q** against the most sensitive cell lines is represented in Table 4. Compounds **2k, 2l, 2o,** and **2q** were the most potent compounds against K-562 leukaemia cell line with IC_50_ 0.44, 0.43, 0.61, and 0.92 µM, respectively. Compound **2o** was the most potent compound against HOP-92 non-small cell lung cancer cell line with IC_50_ 1.41 µM followed by compound **2l** with IC_50_ 1.52 µM and compound **2k** with IC_50_ 1.83μM. Compounds **2l, 2o,** and **2k** were most potent against HT-29 cell line. Compound **2k** was the most potent compound against U251 CNS cancer cell line and OVCAR-4 ovarian cancer cell line with IC_50_ 1.21 µM and 1.66 µM, respectively. On the other hand, compound **2l** was the most potent compound against SK-Mel-5 [melanoma], A498 [renal], PC-3 [prostate], and MDA-MB-468 [breast] with an IC_50_ values of 1.23, 0.33, 1.74, and 1.16 µM, respectively. In general, compounds having 3-hydroxyphenyl and propylene bridge were more potent than the corresponding derivatives with 3-methoxyphenyl and ethylene bridge. Also, compounds with electron-withdrawing group showed high potency than compounds with electron-donating group. Both chloro and bromo substituents were more potent than fluoro compounds.

**Table 4.** IC_50_ values of the most potent compounds over the most sensitive cell lines from each cancer subpanel.

		Cancer cell lines^a^								
		K-562	HOP-92^b^	HT-29^c^	U251^d^	SK-MEL-5^e^	OVCAR-4^f^	A498^g^	PC-3^h^	MDA-MB-468^i^
**Compound No.**	**1c**	3.73	2.46	3.37	5.89	1.95	4.15	4.56	2.44	3.90
	**1g**	2.87	2.12	2.32	3.54	1.72	2.94	3.40	2.86	3.31
	**1k**	1.98	2.00	2.17	2.21	1.64	2.32	3.33	2.07	2.70
	**1l**	1.98	2.34	2.24	3.21	2.02	4.37	2.63	2.45	2.78
	**1m**	1.98	2.03	1.99	2.62	1.73	3.05	2.32	2.29	1.88
	**1o**	2.48	1.97	2.04	2.65	1.78	2.85	1.71	2.47	2.05
	**2g**	3.06	3.68	3.21	5.63	2.18	4.36	3.80	3.36	3.11
	**2h**	3.31	2.42	2.87	4.83	1.86	3.28	3.52	2.97	3.49
	**2k**	**0.44**	1.83	**0.63**	1.21	1.27	1.66	**0.38**	2.11	1.54
	**2l**	**0.43**	1.52	**0.47**	1.35	1.23	1.67	**0.33**	1.74	1.16
	**2m**	2.40	2.66	2.26	2.97	3.06	3.82	3.52	3.11	2.55
	**2o**	**0.617**	1.41	**0.50**	1.78	1.57	3.06	1.09	2.87	1.85
	**2q**	**0.92**	1.56	1.23	1.28	1.53	1.92	1.1	1.93	1.8
**Sorafenib**		3.16	1.85	2.51	2.51	1.25	3.16	2.51	2.51	1.99

^a^Leukaemia cell line; ^b^non-small cell lung cancer cell line; ^c^colon cancer cell line; ^d^CNS cancer cell line; ^e^melanoma cell line; ^f^ovarian cancer cell line; ^g^renal cancer cell line; ^h^prostate cancer cell line; ^i^breast cancer cell line.

The bold values indicate more potent compared to the reference standard compound.

In addition to IC_50_, total growth inhibition (TGI) concentration and lethal dose 50 (LD_50_) for the most potent compounds were determined (Table 5). The total growth inhibition in case of K-562 leukaemia cell line was ranging from 2.5 µM for compound **2l** to 19 µM for compound **1g**. For non-small cell lung cancer cell line HOP-92, compound **2o** exerted the lowest TGI (3.6 µM) and LD_50_ (9.7 µM). Compounds **2l** and **2k** exhibited TGI 4.7 and 7.5 µM, respectively. Compounds **2q** and **2l** were the most efficacious among this new series against HT-29 colon cancer cell line with TGI 1.7 µM for each of them and LD_50_ 4.5 and 4.7 µM, respectively. Compounds **2o** and **2l** showed the highest efficacy against U251 CNS cancer cell line with TGI 3.2 and 3.4 µM, respectively, and LD_50_ 8.1 and 8.8 µM, respectively. All the tested compounds except **2g** and **2m** exhibited significant one-digit micromolar TGI and LD_50_ values against SK-MEL-5 melanoma cell line. Both compounds **2k** and **2 l** showed lethal dose 50 5.3 µM. Compound **2k** had TGI of 2.6 µM, while compound **2l** had TGI equal to 2.5 µM. Compound **2l** was the most potent against OVCAR-4, A498, PC-3 and MDA-MB-468 with TGI 5.9, 2.2, 6.1, and 4.6 µM and LD_50_ 6.9 µM for A498 cell line. Compound **2k** was the second most potent compound against the same cell lines with TGI 8.6, 2.8, 7.8, and 5.6 µM and LD_50_ 11.9 µM against A498 cell line. 

**Table 5.** TGI and LD_50_ values of the most potent compounds over the most sensitive cell lines from each cancer subpanel.

			Cancer cell lines^a^								
			K-562^a^	HOP-92^b^	HT-29^c^	U251^d^	SK-MEL-5^e^	OVCAR-4^f^	A498^g^	PC-3^h^	MDA-MB-468^i^
**Compound No.**	**1c**	TGI	>100	9.4	11.3	>100	3.8	>100	>100	>100	>100
		LD_50_	>100	>100	>100	>100	7.7	>100	>100	>100	>100
	**1g**	TGI	19	6.8	5.7	13.9	3.1	>100	6.5	26.7	>100
		LD_50_	>100	>100	37.7	63.4	5.6	>100	>100	>100	>100
	**1k**	TGI	5.4	5.0	5.0	5.6	3.0	13.7	5.4	6.4	7.7
		LD_50_	>100	34.6	17.3	25.6	5.6	>100	34.3	>100	>100
	**1l**	TGI	5.4	8.3	5.2	11.4	4.0	>100	9.5	12.0	7.66
		LD_50_	>100	70.5	61.8	45.9	7.9	>100	57.1	>100	>100
	**1m**	TGI	>100	8.4	47.9	6.8	3.2	>100	9.2	10.2	5.3
		LD_50_	>100	68.8	54.3	50.9	6.0	>100	45.7	>100	>100
	**1o**	TGI	6.5	6.2	5.4	7.2	3.3	16.9	14.5	10.9	5.02
		LD_50_	>100	52.0	35.1	44.1	6.3	81.2	41.4	93.4	>100
	**2g**	TGI	>100	>100	10	>100	5.0	>100	>100	>100	>100
		LD_50_	>100	>100	>100	>100	23.1	>100	>100	>100	>100
	**2h**	TGI	>100	>100	18.8	>100	3.7	>100	>100	>100	30.4
		LD_50_	>100	>100	>100	>100	7.6	>100	>100	>100	>100
	**2k**	TGI	2.8	7.55	2.3	4.4	2.6	8.6	2.8	7.8	5.6
		LD_50_	>100	>100	7.1	19.9	5.3	>100	11.9	>100	>100
	**2l**	TGI	2.5	4.7	1.7	3.4	2.5	5.9	2.2	6.1	4.6
		LD_50_	>100	93.2	4.7	8.8	5.3	>100	6.9	>100	>100
	**2m**	TGI	6.3	7.6	6.4	10.3	10.1	4.8	9.8	8.5	6.7
		LD_50_	>100	84.2	47.8	69.5	33.0	>100	52.3	80.7	>100
	**2o**	TGI	5.3	3.6	3.1	3.2	2.9	5.1	2.6	4.5	5.5
		LD_50_	>100	9.7	8.1	8.1	5.6	>100	6.3	18.9	>100
	**2q**	TGI	4.4	7.2	1.7	13.2	3.4	>100	6.6	44.9	6.0
		LD_50_	>100	>100	4.5	41.3	7.4	>100	>100	>100	>100

^a^Leukaemia cell line; ^b^non-small cell lung cancer cell line; ^c^colon cancer cell line; ^d^CNS cancer cell line; ^e^melanoma cell line; ^f^ovarian cancer cell line; ^g^renal cancer cell line; ^h^prostate cancer cell line; ^i^breast cancer cell line.

#### Kinase profiling

In order to determine the molecular target(s) of the newly synthesized compounds, a panel of 20 kinases of different families was used. The inhibitory effects of compounds **1l, 2c,** and **2l**, which showed the strongest potencies against the NCI-60 cancer cell line panel, on different kinases are depicted in Table 6. It can be concluded that compound **2l** had a strong activity against JNK1, JNK2, JNK3, P38a/MAPK14, and BRAF (V600E) with mean per cent inhibitions 99.02%, 98.47%, 89.50%, 86.54%, and 93.67%, respectively. Also, compound **2l** showed a moderate activity against GSK3b and BRAF with mean per cent inhibitions 75.26% and 72.56%, respectively. On the other hand, compounds **1l** and **2c** showed moderate activity against JNK1, JNK2, and BRAF(V600E). The stronger kinase inhibitory effects of the hydroxyl compound **2l** compared to the corresponding methoxy derivative **2c** can be rationalized that the presence of hydrogen bond donor and the lower bulkiness of OH group may contribute to stronger affinity with the enzymes. Furthermore, the propylene linker of **2l** seems to be more appropriate for activity than ethylene.

**Table 6.** Inhibitory effect of compounds **1l**, **2c**, and **2 l** on different kinases activity at a single dose of 10 µM.

Kinase	Inhibition %		
	1l	2c	2l
ABL1 (T315I)	6.39 ± 0.35%	4.53 ± 0.45%	–10.98 ± 0.40%
BRAF	21.28 ± 0.21%	20.87 ± 0.30%	72.56 ± 0.27%
BRAF (V599E)	48.09 ± 0.45%	44.20 ± 0.40%	93.67 ± 0.47%
CDK2/cyclin E	8.36 ± 0.95%	9.80 ± 1.01%	22.92 ± 1.14%
EGFR	10.33 ± 3.40%	9.20 ± 4.45%	11.74 ± 4.78%
ERK1	ND	ND	13.07 ± 0.93%
ERK2/MAPK1	ND	ND	22.21 ± 0.41%
FMS	ND	ND	24.24 ± 1.14%
GSK3b	15.88 ± 0.21%	49.31 ± 0.30%	75.45 ± 0.28%
IKKb/IKBKB	7.80 ± 0.10%	7.79 ± 0.15%	−2.94 ± 0.17%
JNK1	53.79 ± 0.04%	69.19 ± 0.02%	99.05 ± 0.03%
JNK2	44.68 ± 0.01%	59.68 ± 0.01%	98.49 ± 0.02%
JNK3	35.70 ± 0.04%	24.23 ± 0.03%	89.50 ± 0.03%
KDR/VEGFR2	ND	ND	10.05 ± 0.10%
MEK1	ND	ND	0.56 ± 1.08%
MEK2	ND	ND	11.73 ± 1.60%
MKK6	ND	ND	16.65 ± 0.11%
mTOR/FRAP1	ND	ND	0.72 ± 0.32%
P38a/MAPK14	68.43 ± 0.59%	65.73 ± 0.66%	86.54 ± 0.61%
ROS/ROS1	ND	3.40 ± 0.39%	24.03 ± 0.51%

ND: not determined.

ICs_50_ of compound **2l** against its target enzymes are tabulated in Table 7. Compound **2l** exhibited an IC_50_ 0.35 µM over JNK1 and 0.36 µM over JNK2. JNK kinases are hyperactivated in different types of cancer such as leukaemia, lung cancer, skin cancer, glioblastoma and other brain tumours, and breast cancer. JNK kinases induce tumorigenesis through induction of cell proliferation and survival, and their inhibition is a potential avenue for cancer therapy^24^. So inhibition of JNK1 and JNK2 kinases could be, at least partially, a possible mechanism of antiproliferative activity of compound **2l**.

**Table 7.** IC_50_ of compound **2l** against BRAF, BRAF(V600E), JNK1, JNK2, P38a/MAPK14 and RAF1.

Compound	BRAF	BRAF(V600E)	JNK1	JNK2	MAPK14	RAF1
**2l**	4.87 ± 0.07	1.16 ± 0.04	0.35 ± 0.04	0.36 ± 0.07	1.13 ± 0.09	3.52 ± 0.01

#### Plasma stability

The plasma stability test for compound **2l** revealed that compound **2l** has high stability profile in both human and rat plasma. After 30 min, 100% of compound **2 l** remained unchanged which decreased to 95.3% after 2 h, while that of procaine was 1.2% after 30 min and 0.2% after 2 h and the percentage for diltiazem was 91.5% after 30 min and 89.3% after 2 h (Table 8).

**Table 8.** Plasma stability of compound **2l**.

Compound	Human		Rat	
	30 min	120 min	30 min	120 min
**2l**	100%	95.3%	100%	100%
Procaine	1.2%	0.2%	89.9%	42.7%
Diltiazem	91.5%	89.3%	86.3%	45.4%

hERG binding assay.

One of the most important features to be investigated during drug development is the ability of the compound to bind to the human ether-a-go-go related gene (hERG). The inhibition of hERG can lead to sudden death and should be avoided during drug discovery^25^. Compound **2l** was tested for its ability to inhibit hERG and the IC_50_ was 8.28 µM which is fifteen times greater than its IC_50_ against the most sensitive cell line. So the compound has high safety and selectivity index, and low risk of sudden death induction.

#### *In vivo* pharmacokinetic

**Figure 3.**
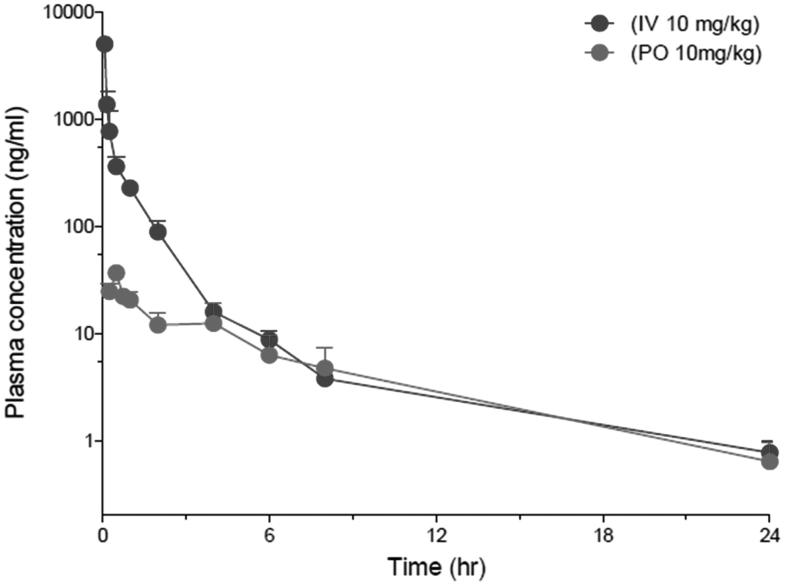
Plasma concentration-time curves of **2l** in rats following iv (10 mg/kg) or oral (10 mg/kg) administration.

Compound **2 l** was the most potent compound against both *in vitro* cell line assay and enzyme activity. The human plasma stability is excellent compared to standard reference compounds. The *in vivo* pharmacokinetic profile following oral or intravenous administration, and oral bioavilability of **2 l** are represented in Table 9 and Figure 3.

**Table 9.** PK Parameters of **2l** in SD Rats (*N* = 3).

PK parameter	**2l** (IV, 10 mg /kg)			**2l** (PO, 10 mg /kg)		
	Mean	SD	*N*	Mean	SD	N
Dose (mg/kg)	10		3	10		3
T_max_ (hr)	NA			0.5	0.0	3
C_max_ (ng/ml)	NA			36.6	4.9	3
AUC_last_ (ng.hr/ml)	1471.6	156.2	3	136.0	46.7	3
AUC_inf_ (ng.hr/ml)	1478.2	157.3	3	142.1	44.8	3
CL (L/hr/kg)	6.8	0.8	3	NA		
*t*_1/2_ (hr)	5.8	1.0	3	6.1	2.2	3
F (%)	NA			9.2	3.2	3

## Conclusion

In the current work, design and synthesis of a new N-(2-((4-(3-(3-methoxy and/or hydroxyl)phenyl)-1-phenyl-1H-pyrazol-4-yl)pyridin-2-yl)amino)ethyl and/or propyl) substituted benzene sulfonamides **1a–q** and **2a–q** were accomplished. The antiprolifertave activity of the new synthesized compounds revealed that compounds having propyl bridge between the pyridine ring and sulfonamide moiety **2a–q** were more potent compared to compounds having ethylene bridge **1a–q**. In addition, compounds possessing *m*-hydroxyl group at ring A were more potent than their methoxy analogues with an exception of compounds **2g** and **2h** which carry out terminal *p-*triflouromethyl sulfonamide and *m-*fluoro sulfonamide moieties which showed excellent activity compared to other methoxy compounds. Compounds with electron-withdrawing groups such as chloro **2l** and bromo **2k** presented the highest per cent inhibition among the newly synthesized compounds and lowest IC_50_ 0.33 µM against A498 renal cancer cell line for compound **2l**. Compound **2l** significantly inhibited the activity of JNK1, JNK2, JNK3, V600E BRAF, and P38 alpha with IC_50_ 0.35 and 0.36 µM against JNK1 and JNK2. Moreover, compound **2l** exhibited high plasma stability in both human and rat. Compound **2l** had 9.2% oral bioavailability with t_1/2_ ranging from 5.8 h for intravenously administrated to 6.1 h for orally administrated doses.

## Supplementary Material

IENZ_1530225_Supplementary Material
