# Supplementary material for: Design, synthesis, in vitro anticancer evaluation, kinase inhibitory effects, and pharmacokinetic profile of new 1,3,4-triarylpyrazole derivatives possessing terminal sulfonamide moiety
Source: J Enzyme Inhib Med Chem. 2018 Oct 26;34(1):97–109. doi: 10.1080/14756366.2018.1530225 (PMC6211260; doi:10.1080/14756366.2018.1530225)

**Design, synthesis, *in vitro* anticancer evaluation, kinase inhibitory effects, and pharmacokinetic profile of new 1,3,4-triarylpyrazole derivatives possessing terminal sulfonamide moiety**

Mohammed S. Abdel-Maksoud^a^, Mohammed I. El-Gamal^b,c,d^, Mahmoud M. Gamal El-Din^a^, and Chang Hyun Oh^e,f*^

^a^ Medicinal & Pharmaceutical Chemistry Department, Pharmaceutical and Drug Industries Research Division, National Research Centre (NRC), Dokki, Giza, 12622, Egypt.,^b^ Department of Medicinal Chemistry, College of Pharmacy, University of Sharjah, Sharjah 27272, United Arab Emirates., ^c^ Sharjah Institute for Medical Research, University of Sharjah, Sharjah 27272, United Arab Emirates., ^d^ Department of Medicinal Chemistry, Faculty of Pharmacy, University of Mansoura, Mansoura 35516, Egypt., ^e^ Center for Biomaterials, Korea Institute of Science and Technology (KIST), Cheongryang, Seoul 130-650, Republic of Korea.,^f^ Department of Biomolecular Science, University of Science and Technology (UST), Daejeon, Yuseong-gu, 34113, Republic of Korea.

*Corresponding author. E-mail address: choh[@kist.re.kr](mailto:khyoo@kist.re.kr); Address: Center for Biomaterials, Korea Institute of Science and Technology, PO Box 131, Cheongryang, Seoul 130-650, Republic of Korea [C.-H. Oh].

Table of contents

| Title page | 1s |
| --- | --- |
| Table of contents | 2s |
| Experimental | 3s-5s |
| NCI-60 cell lines data | 6s-57s |
| Representative NMR charts | 58s-120s |

**Experimental**

***Synthesis of methyl benzoate (4)***

A solution of 3-methoxybenzoic (**3**) (304 mg, 2.0 mmol) in methanol (5 ml) were heated under reflux until the acid was completely dissolved in methanol then few drops of concentrated sulphuric acid was added to the mixture and refluxed for 8 hr. The resulting mixture was cooled to room temperature, diluted with water and a saturated solution of sodium bicarbonate was added to the mixture to neutralize the benzoic acid, extracted with ethyl acetate, dried and evaporated to get the required ester (300 mg, 90.3%) as yellow liquid; ^1^H NMR (400 MHz, CDCl_3_) δ 8.15 (dd, *J* = 9.0 Hz, 2H, Ar-H), 7.49 (t, *J* = 9.0 Hz, 1H, Ar-H), 7.37 (t, *J* = 9.0 Hz, 2H, Ar-H), 3.85 (s, 3H, OCH_3_ ester), 3.76 (s, 3H, OCH_3_); ^13^C NMR (100 MHz, CDCl_3_) δ 166.8, 132.8, 130.1, 129.5, 128.2 (Ar-C), 55.2 (OCH_3_), 51.8 (OCH_3_ ester).

***Synthesis of 2-(2-bromopyridin-4-yl)-1-(3-methoxyphenyl) ethan-1-one (5)***

To a solution of methyl benzoate (**4**) (775 mg, 5.0 mmol) and 2-bromo-4-picoline (0.5 mL, 5.6 mmol) in anhydrous THF (5 mL) in a cooled bath at -25 ºC, LiHMDS (3.7 mL, 1.0M solution in THF, 19.9 mmol) was slowly added to maintain the temperature at -25 °C. The resulting mixture was stirred overnight at room temperature. The mixture was quenched with saturated aqueous NH_4_Cl. Ethyl acetate was added and the organic layer was separated. The aqueous layer was extracted with ethyl acetate (3 x10 mL). The combined organic layer extracts were washed with brine and dried over anhydrous Na_2_SO_4_. The organic solvent was evaporated under reduced pressure and the residue was purified by flash column chromatography (silica gel, hexane ethyl acetate 12:1 v/v then switching to hexane-ethyl acetate 10:1 v/v) to yield 2-(2-Bromopyridin-4-yl)-1-(3-methoxyphenyl) ethan-1-one (**5**) (1.0 g, 69.9 %) as light yellow solid; m.p.85-88ºC; ^1^H NMR (400 MHz, CDCl_3_) δ 8.29 (d, *J* = 5.2 Hz, 1H, Ar-H), 7.52 (d, *J* = 7.2 Hz, 1H, Ar-H), 7.56-7.48 (m, 1H, Ar-H), 7.40 (t, *J* = 8.0 Hz, 2H, Ar-H), 7.15 (m, 2H, Ar-H), 4.25 (s, 2H, CH_2_), 3.84 (s, 3H, OCH_3_); ^13^C NMR (100 MHz, CDCl_3_) δ 160.0 (C=O), 150.0, 146.5, 137.3, 129.9, 129.2, 124.2, 121.0, 120.2, 112.8 (Ar-C), 55.5 (OCH_3_), 44.0 (CH_2_). LC-MS (m/z) calculated for C_14_H_12_BrNO_2_: 306.16 found 307.20 (M+1) ^+^.

***Synthesis of 2-bromo-4-(3-(3-methoxyphenyl)-1-phenyl-1H-pyrazol-4-yl) pyridine (7)***

A solution of (1.16 g, 3.8 mmol) of compound **5** in DMF-DMA (5.14 ml, 38.2 mmol) was refluxed for 18 h. The solution was cooled down and concentrated under reduced pressure. The residue, which contains compound **6** was dissolved in 5ml of anhydrous ethanol. Phenyl hydrazine (0.394 ml, 4 mmol) was added to the ethanolic solution and the mixture was stirred overnight at room temperature. Water (5 mL) was added to the reaction mixture and the organics were extracted with ethyl acetate (3 x 15 mL). The combined organic layer extracts were washed with brine and dried over anhydrous Na_2_SO_4_. After evaporation of the organic solvent, the residue was purified by column chromatography (silica gel, hexane-ethyl acetate 100:1 v/v) to yield the title compound 2-bromo-4-(3-(3-methoxyphenyl)-1-phenyl-1H-pyrazol-4-yl)pyridine (**7**) (729 mg, 48 %) yellow solid ; mp 96-98°C; IR (KBr, Cm^-1^): 3078, 2964, 1593, 1262; ^1^H NMR (400 MHz, CDCl_3_) δ 8.16 (d, *J* = 5.2 Hz, 1H, Ar-H), 7.98 (s, 1H, ar-H), 7.38-7.37 (m, 1H, Ar-H), 7.31-7.23 (m, 5H, Ar-H), 7.00 (dd, *J* = 5.2, *J* = 1.2 Hz, 1H, Ar-H), 6.94-6.91 (m, 1H, Ar-H), 6.77-6.75 (m, 1H, Ar-H), 6.68-6.66 (m, 1H, Ar-H), 3.66 (s, 3H, OCH_3_); ^13^C NMR (100 MHz, CDCl_3_) δ 159.9, 150.0, 143.6, 142.6, 140.7, 139.3, 130.3, 128.9, 127.9, 125.7, 125.1, 122.5, 120.9, 118.3, 115.5, 115.2 (Ar-C), 55.3 (OCH_3_); LC-MS(m/z) calculated for C_21_H_16_BrN_3_O (m/z):406.05 found: 407.0 (M + 1)^+^.

***Synthesis of N^1^-(4-(3-(3-Methoxyphenyl)-1-phenyl-1H-pyrazol-4-yl)pyridin-2-yl)ethane1,2-diamine (8) and N^1^-(4-(3-(3-Methoxyphenyl)-1-phenyl-1H-pyrazol-4-yl)pyridin-2-yl)propane-1,3-diamine (9)***

A mixture of compound **7** (17.09 g, 42.2 mmol) and Copper Iodide (0.95g, 5mmol) in 50 ml of ethylene diamine or 1,3-diaminopropane was heated at 100 degree for 24h. The reaction mixture was treated with water (150 mL) and ethyl acetate (150 ml). The organic layer was collected and washed with additional water (100 mL) then dried over Na_2_SO_4_ and evaporated to get the required product as grayish white solid, which was dried and used in the next step without further purification.


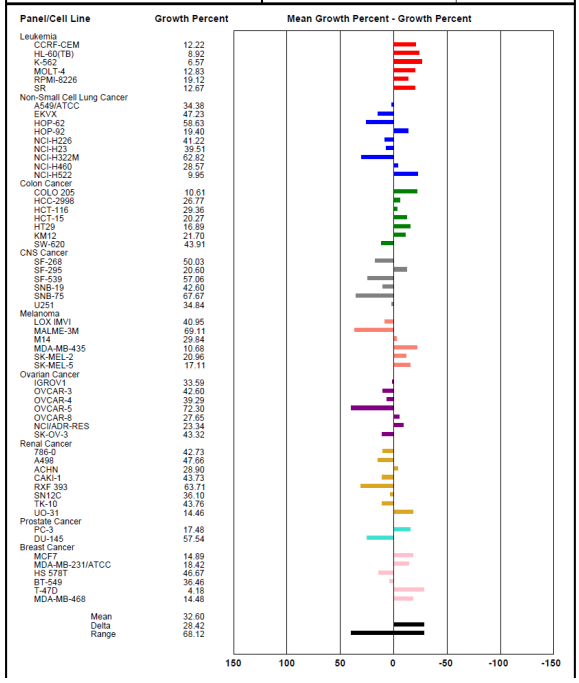


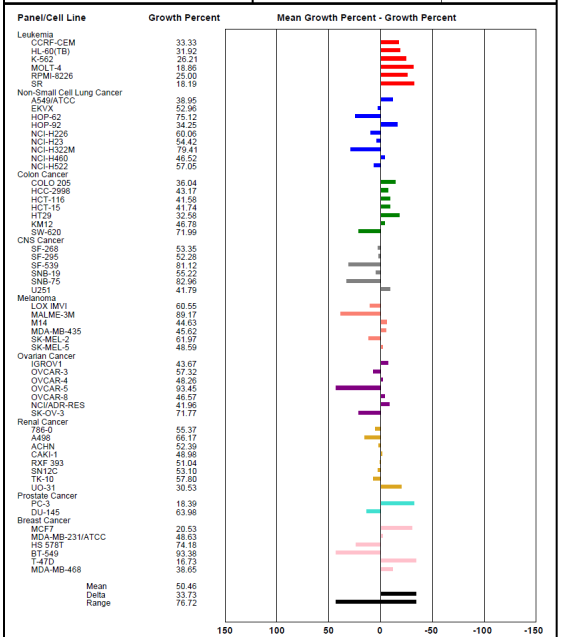


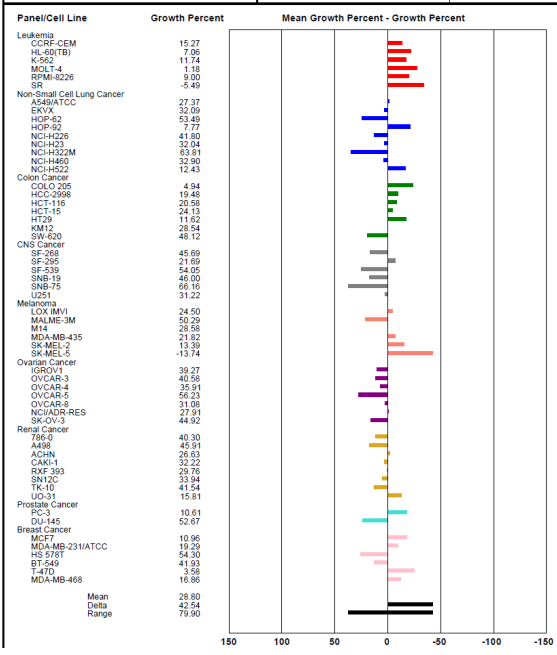


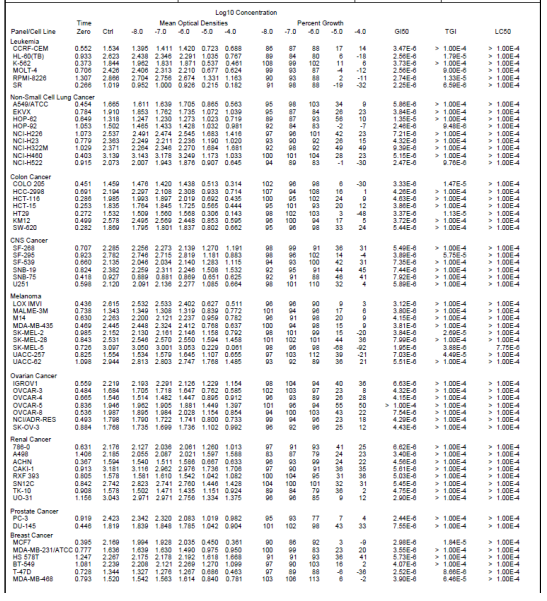


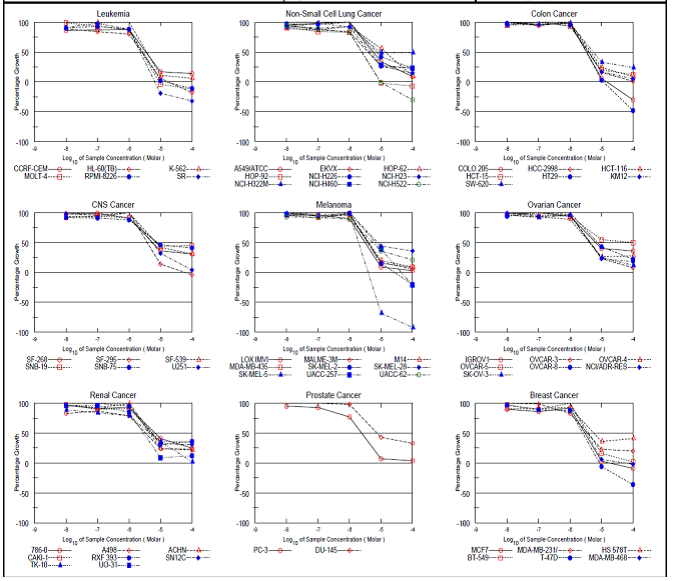


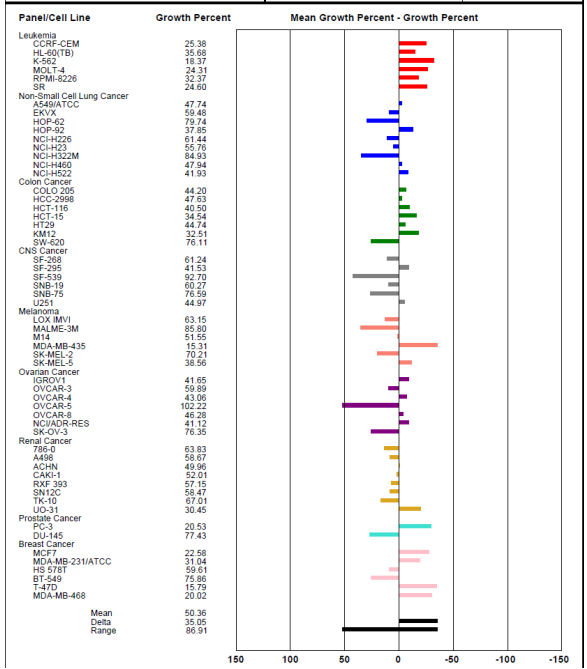


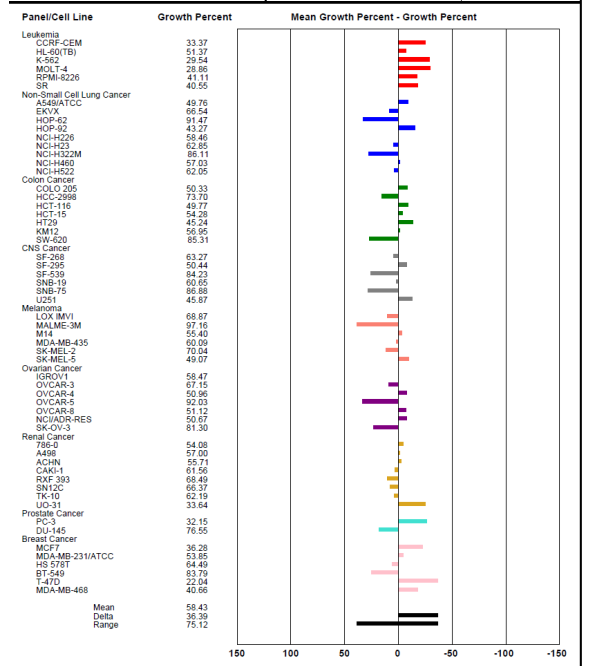


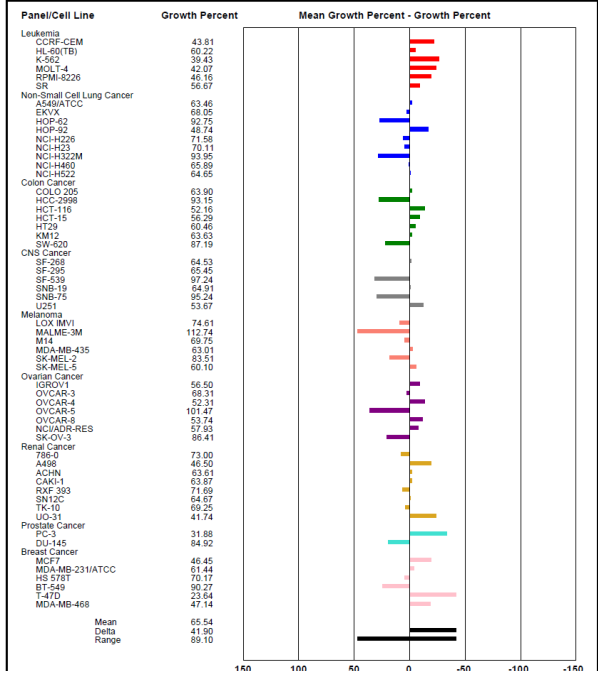


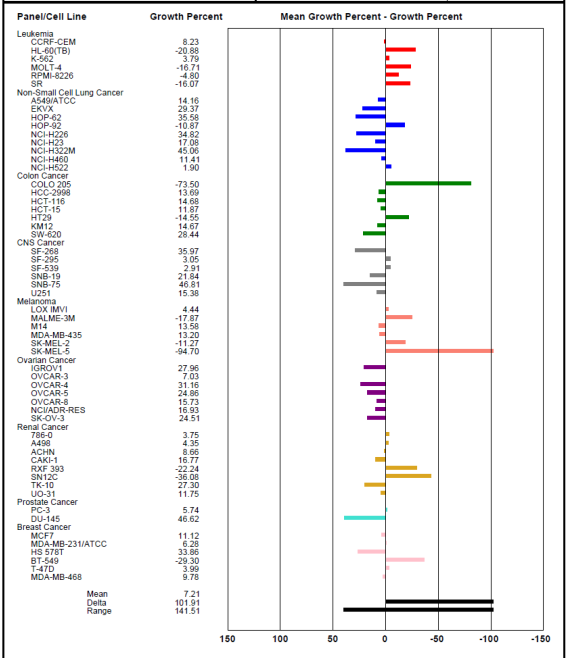


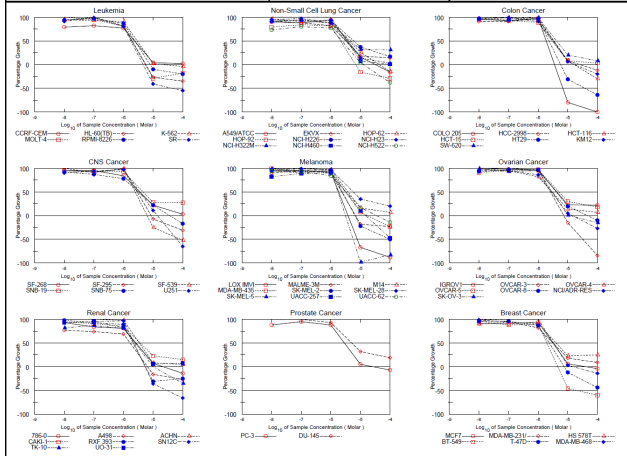


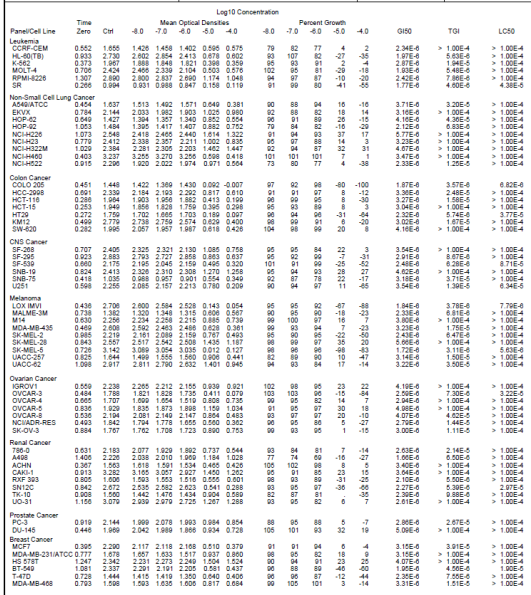


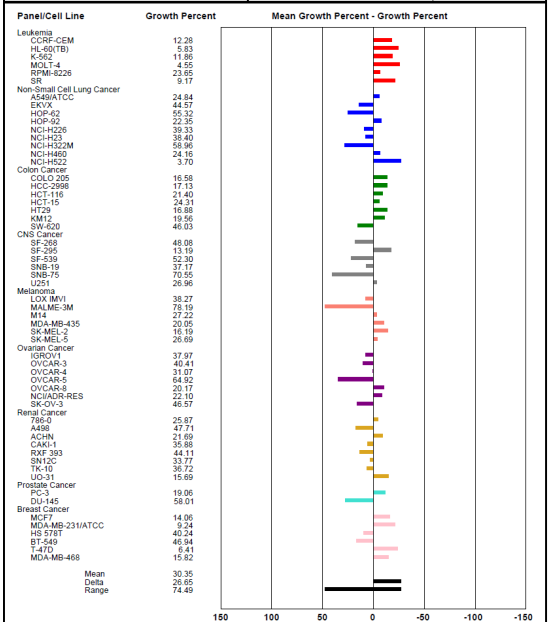


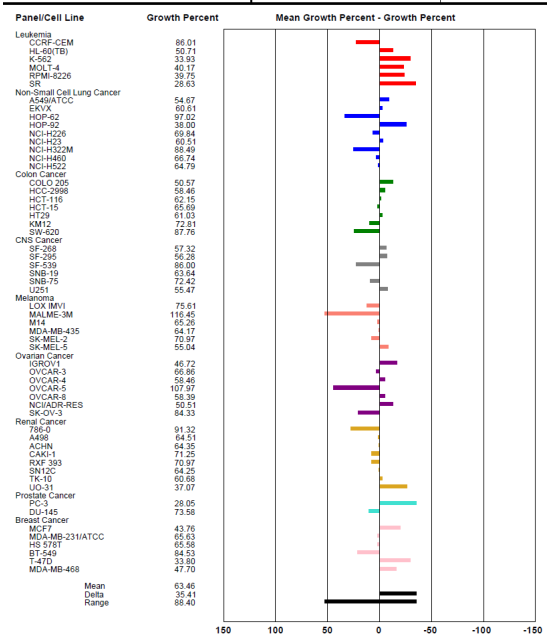


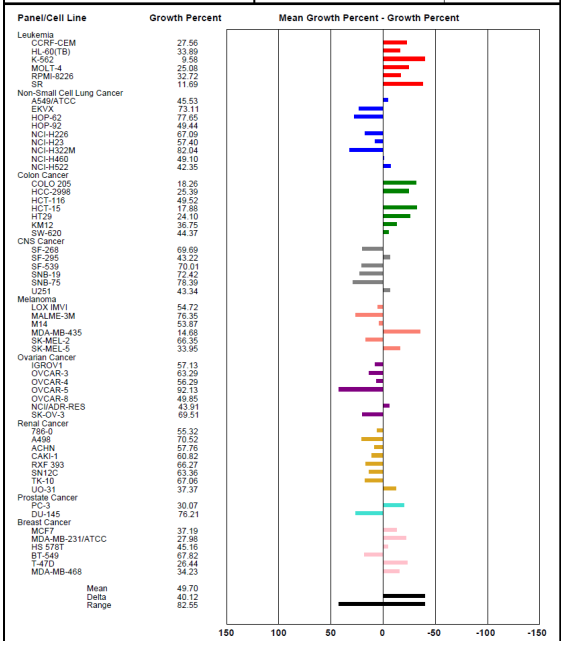


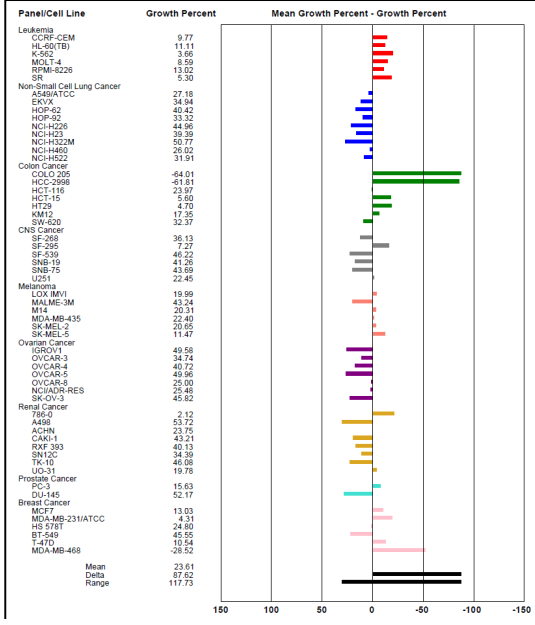


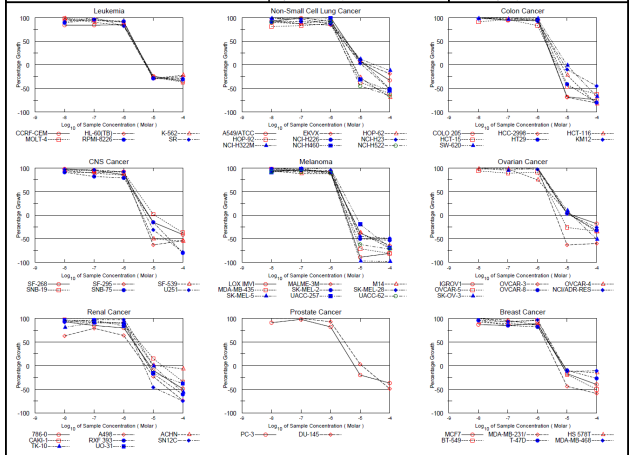


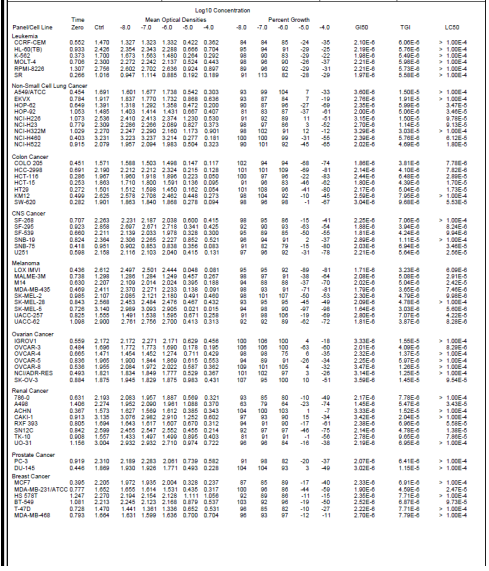


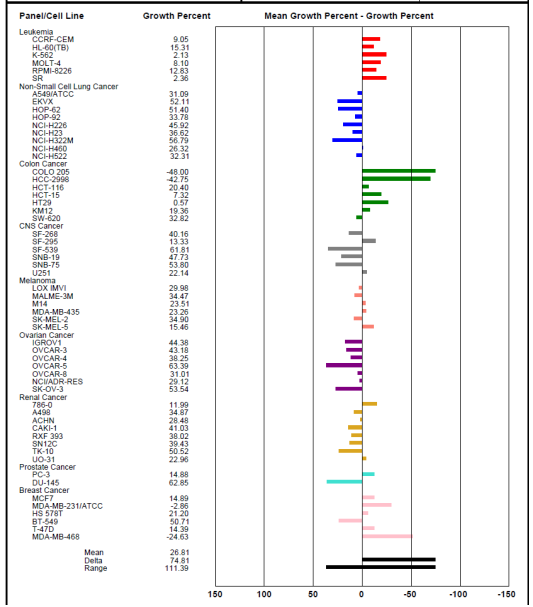


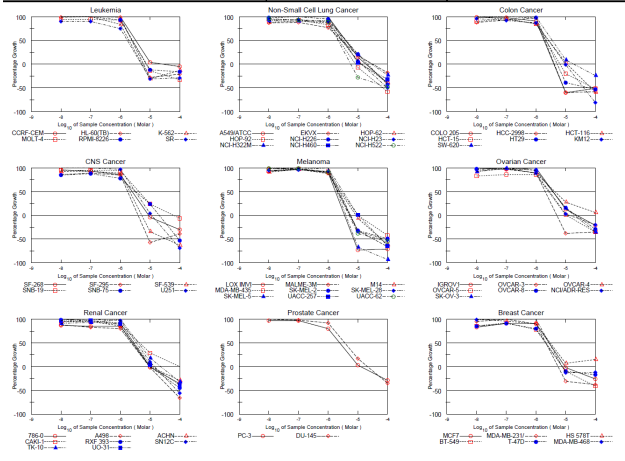


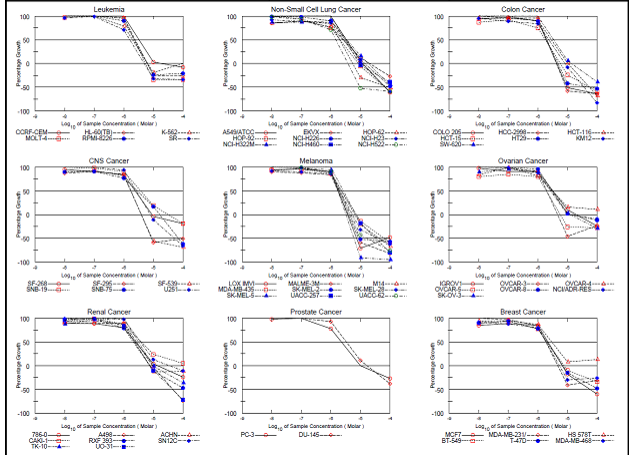


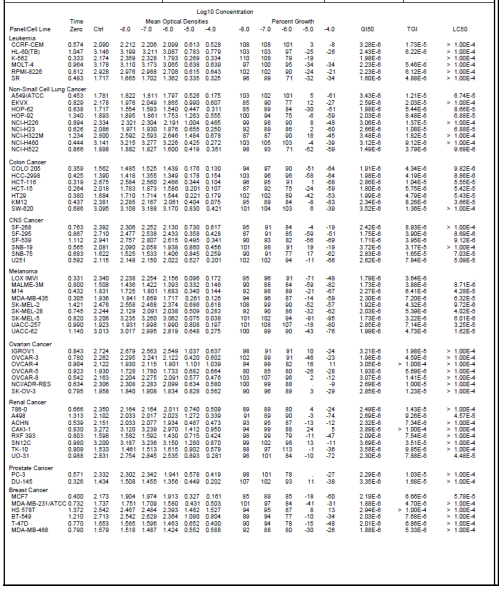


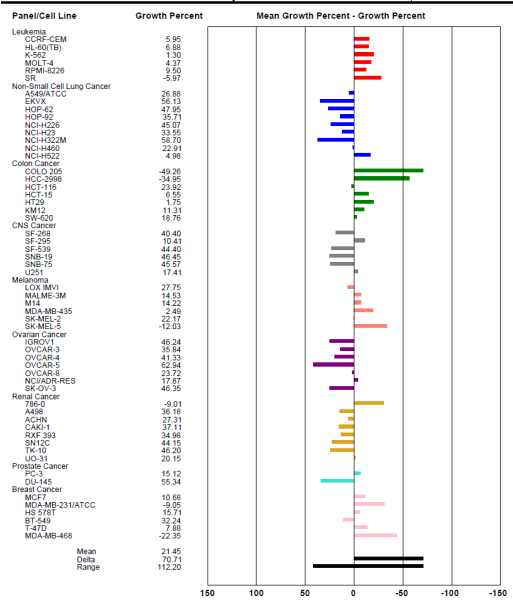


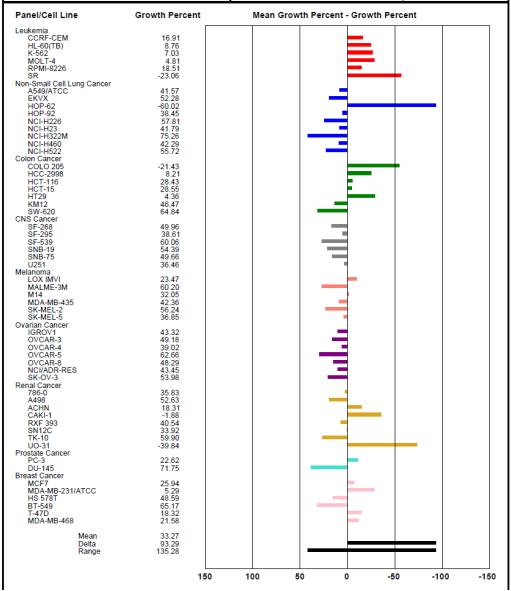


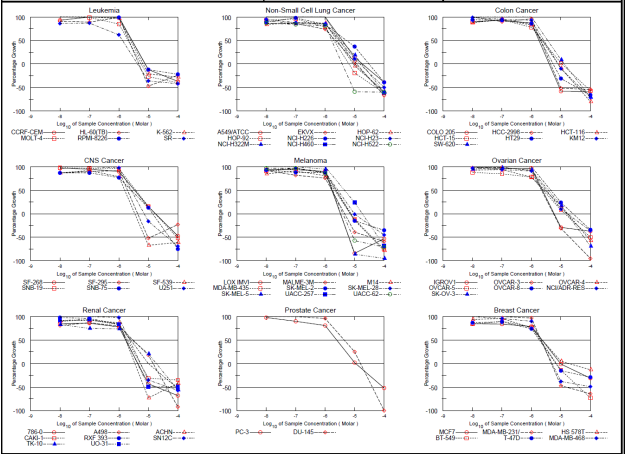


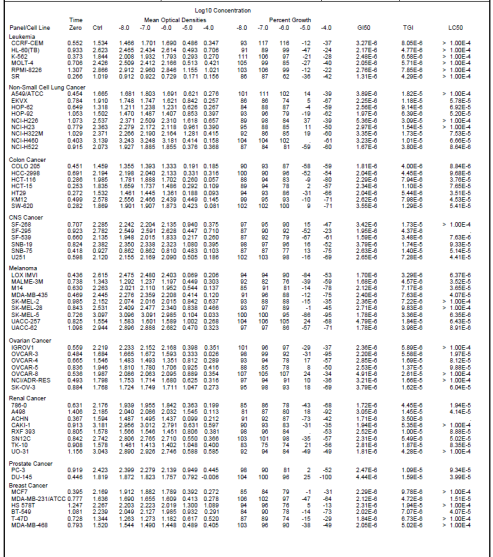


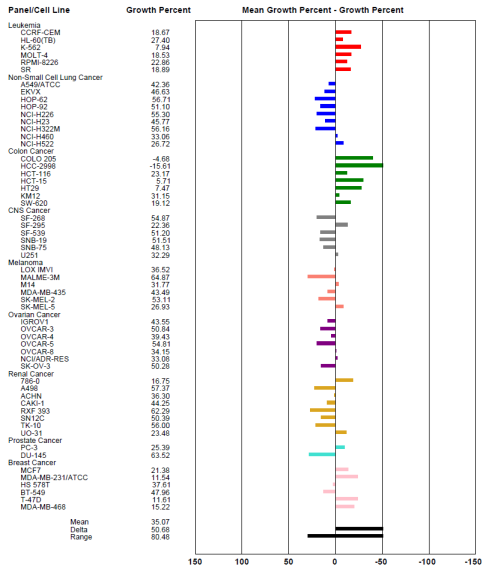


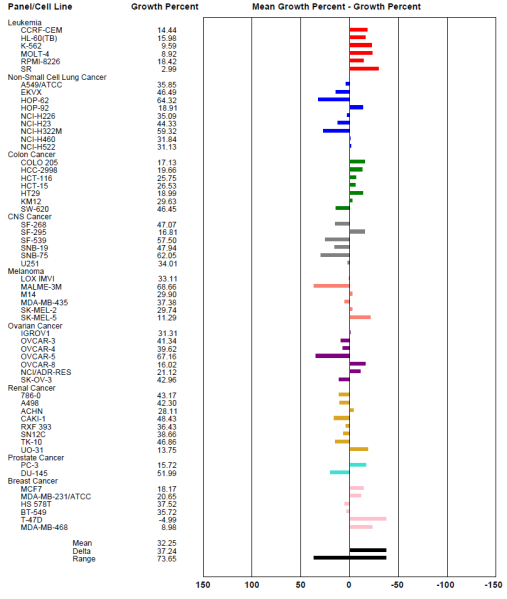


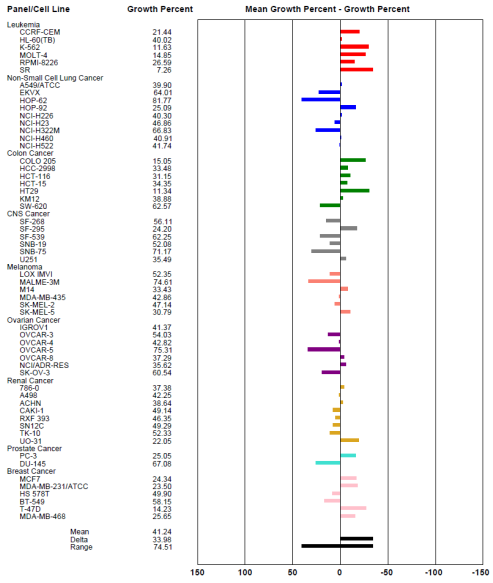


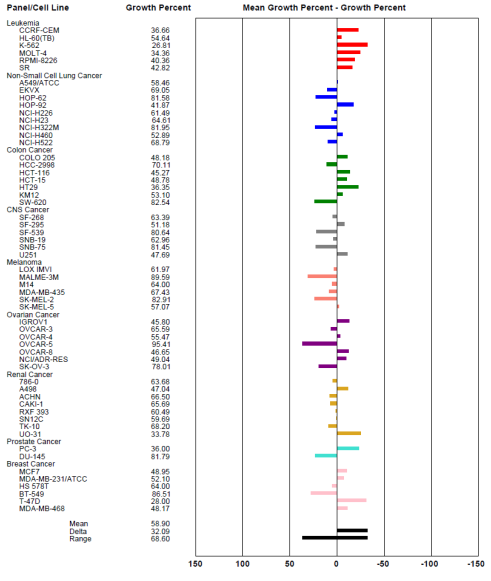


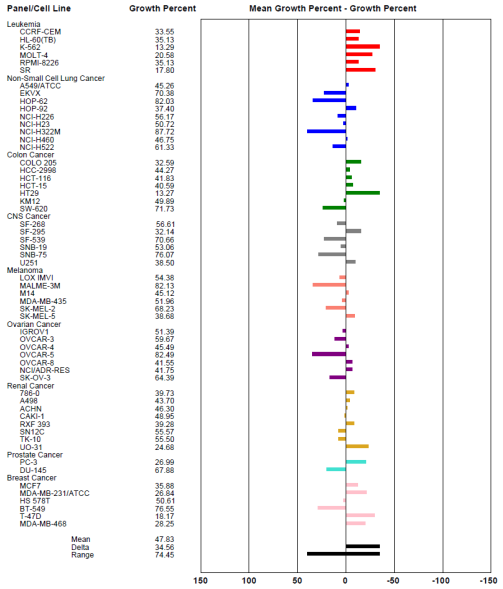


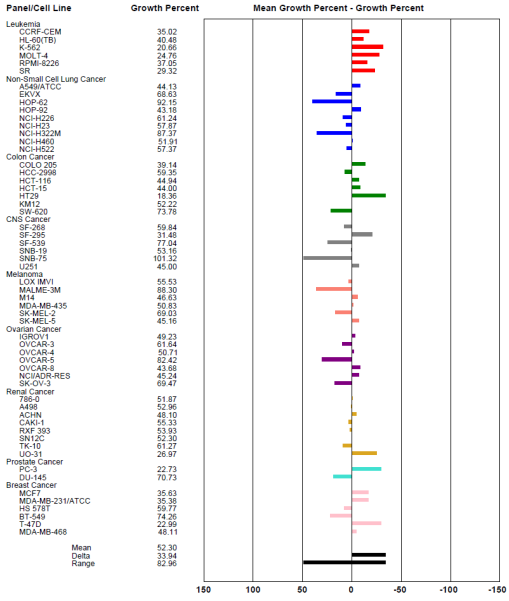


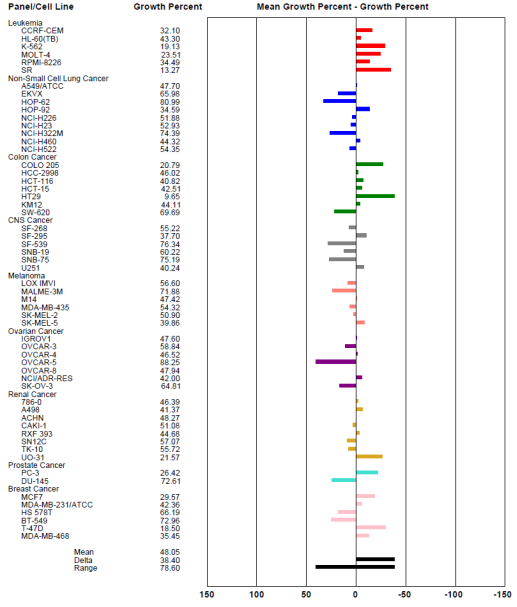


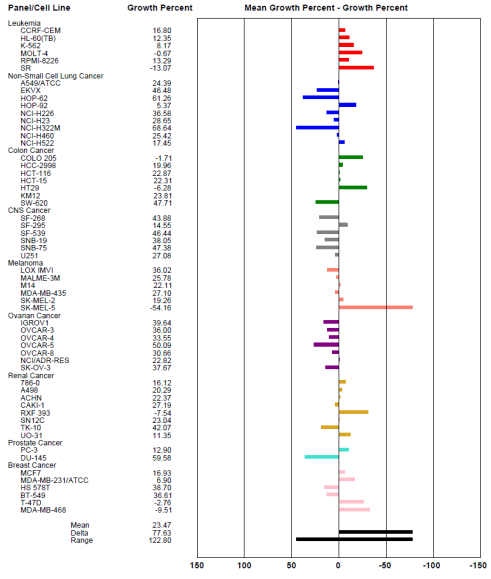


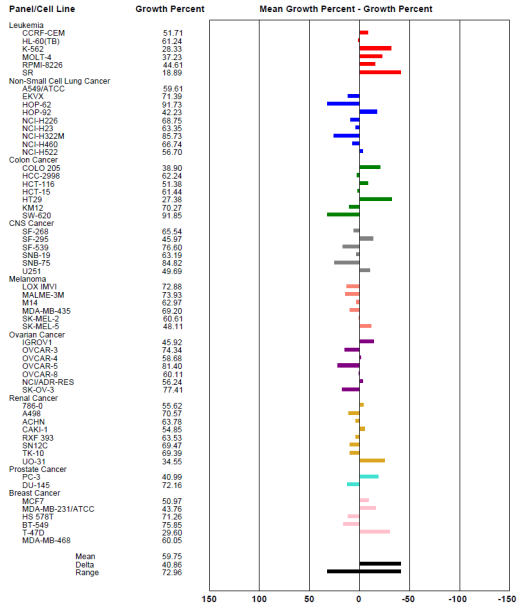


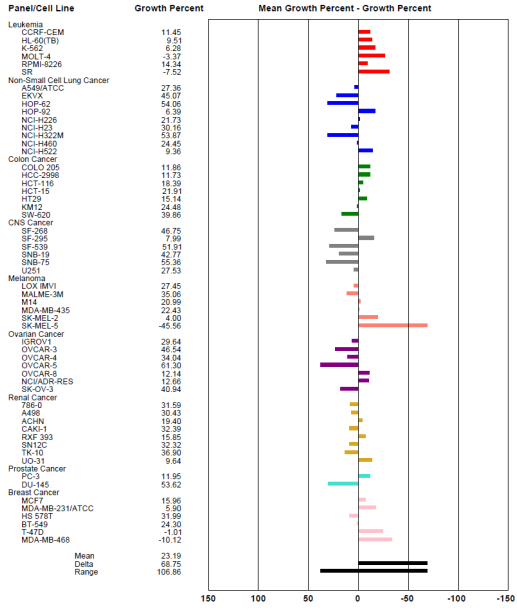


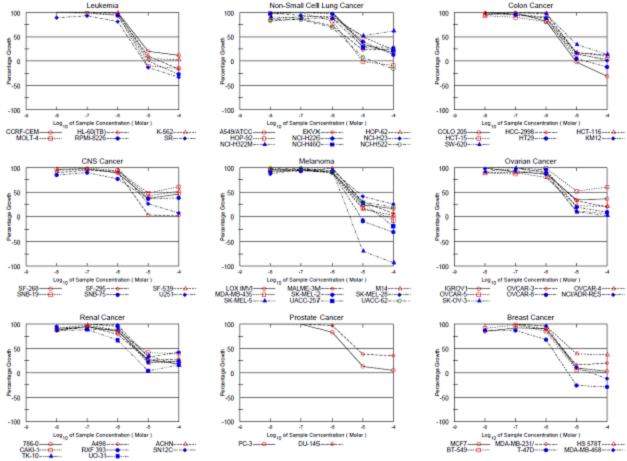


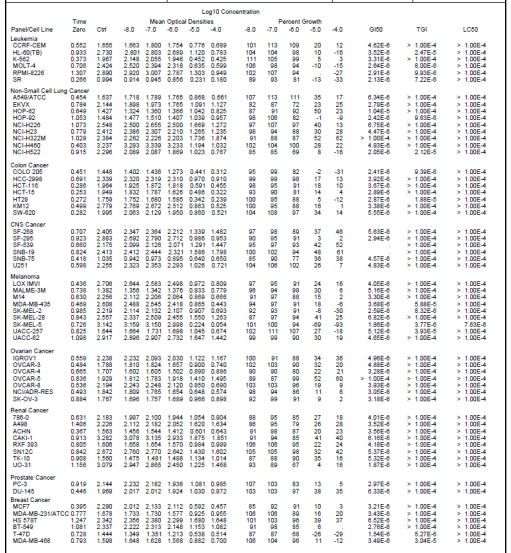


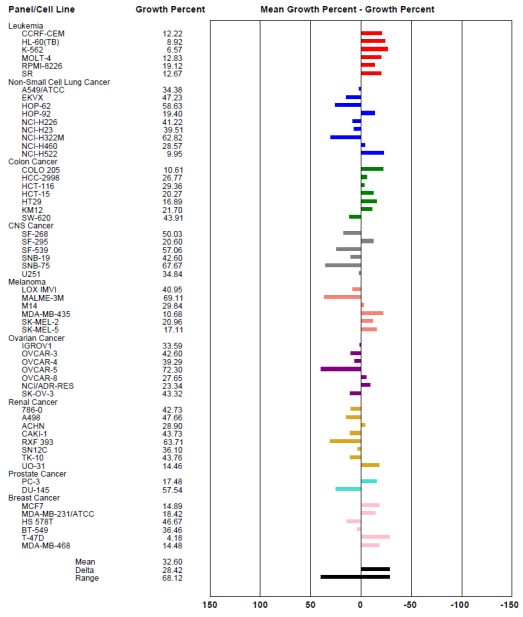


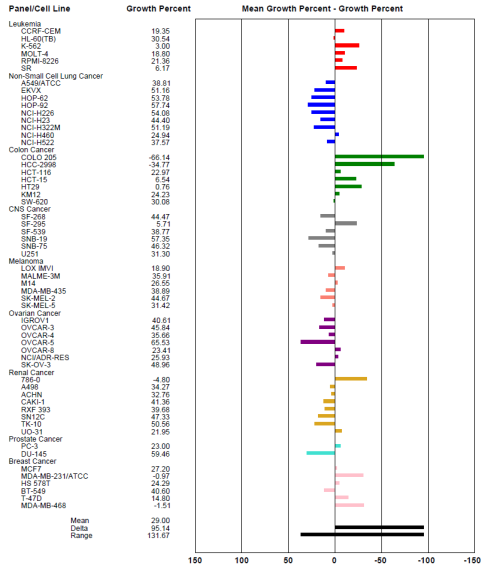


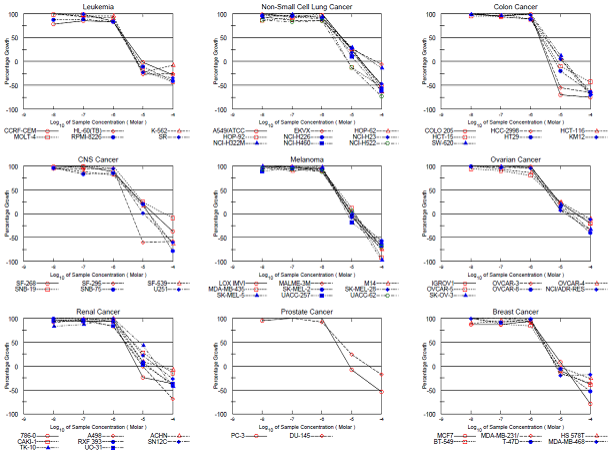


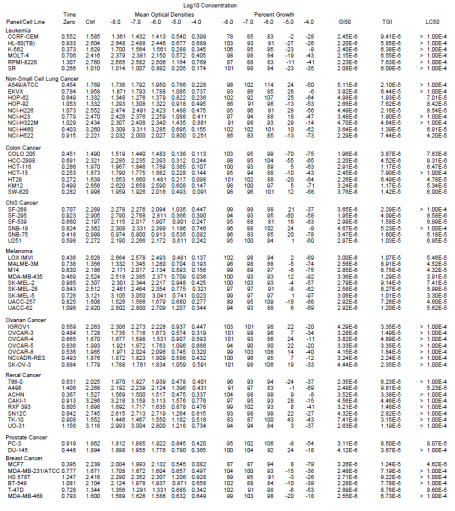


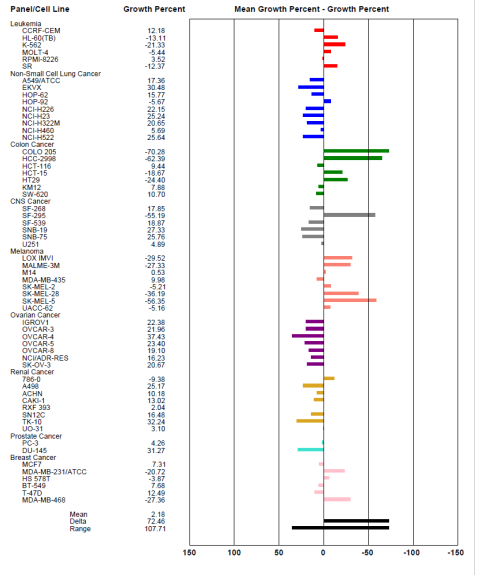


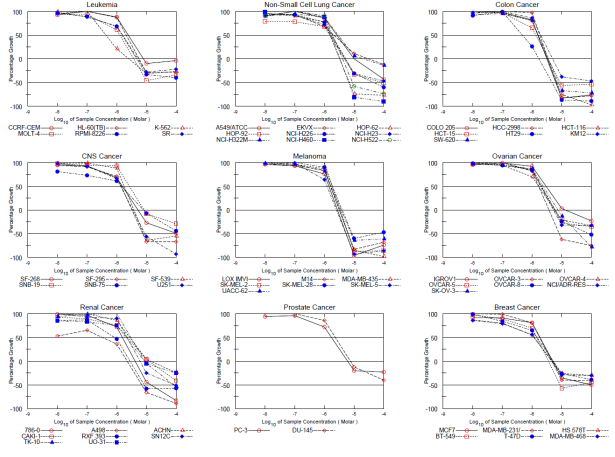


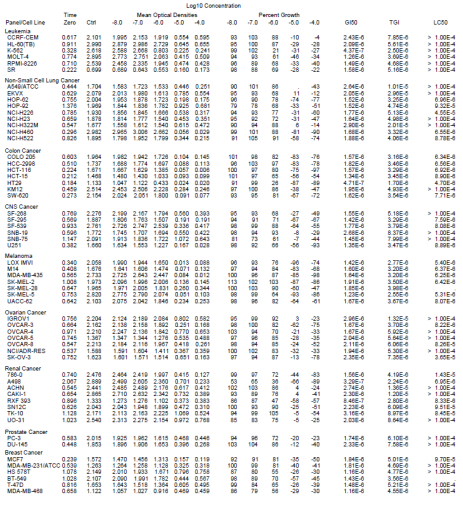


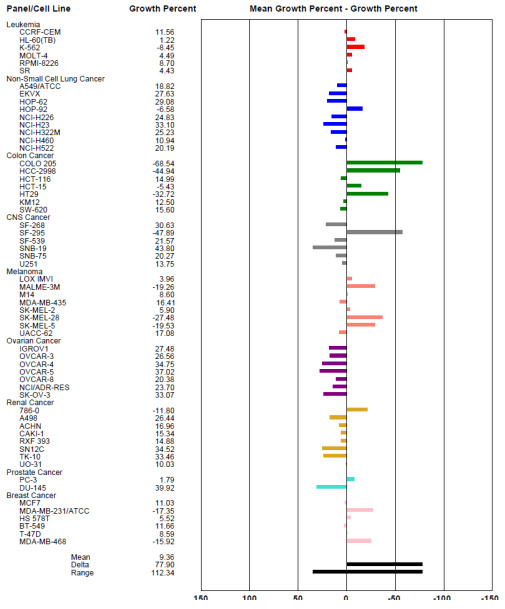


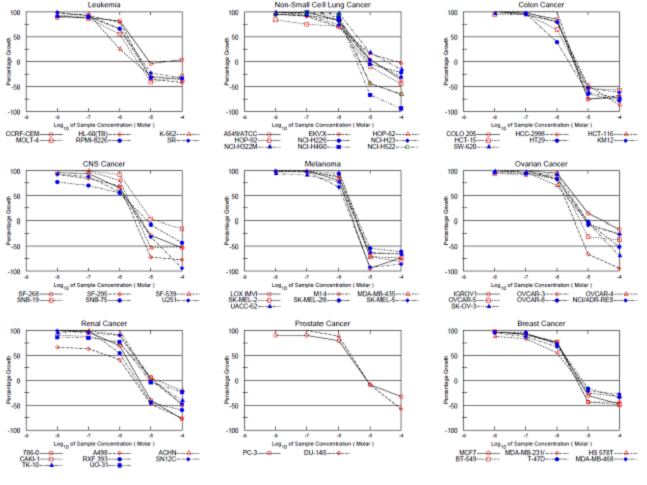


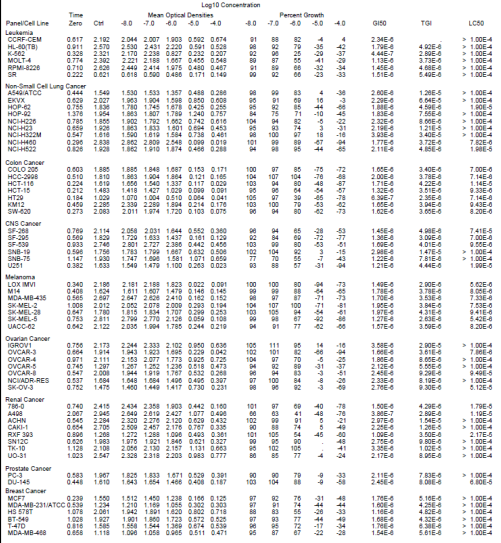


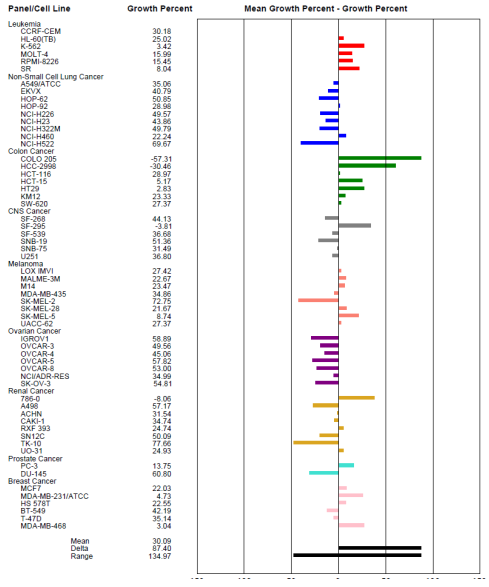


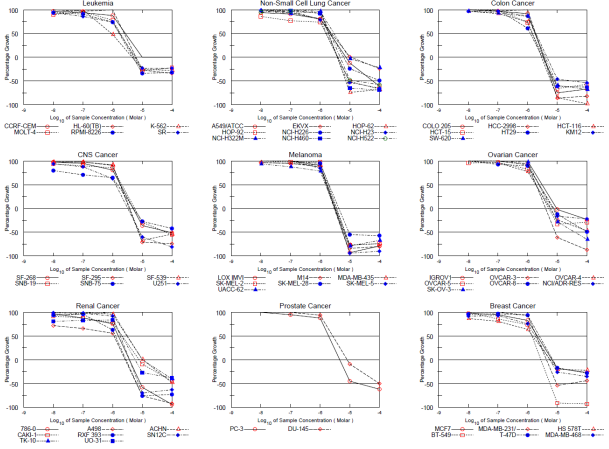


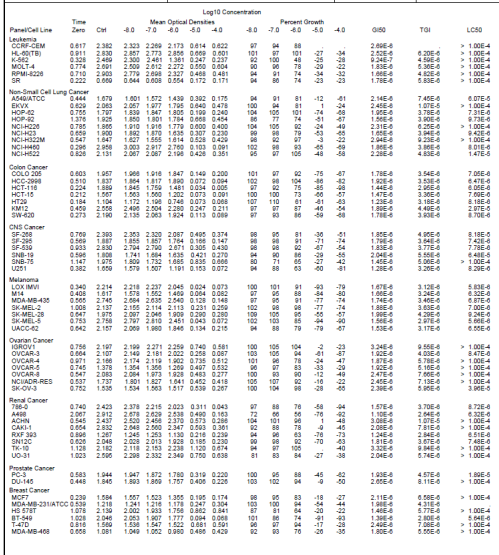


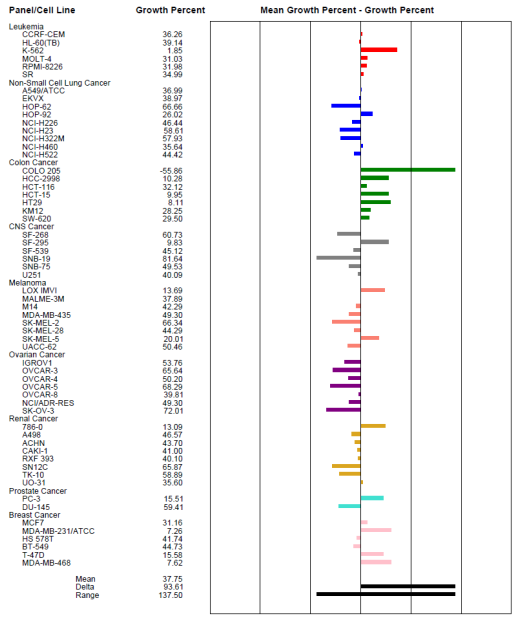


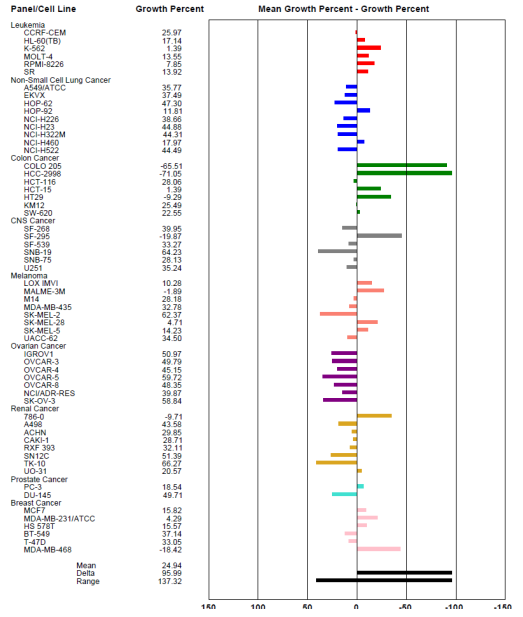


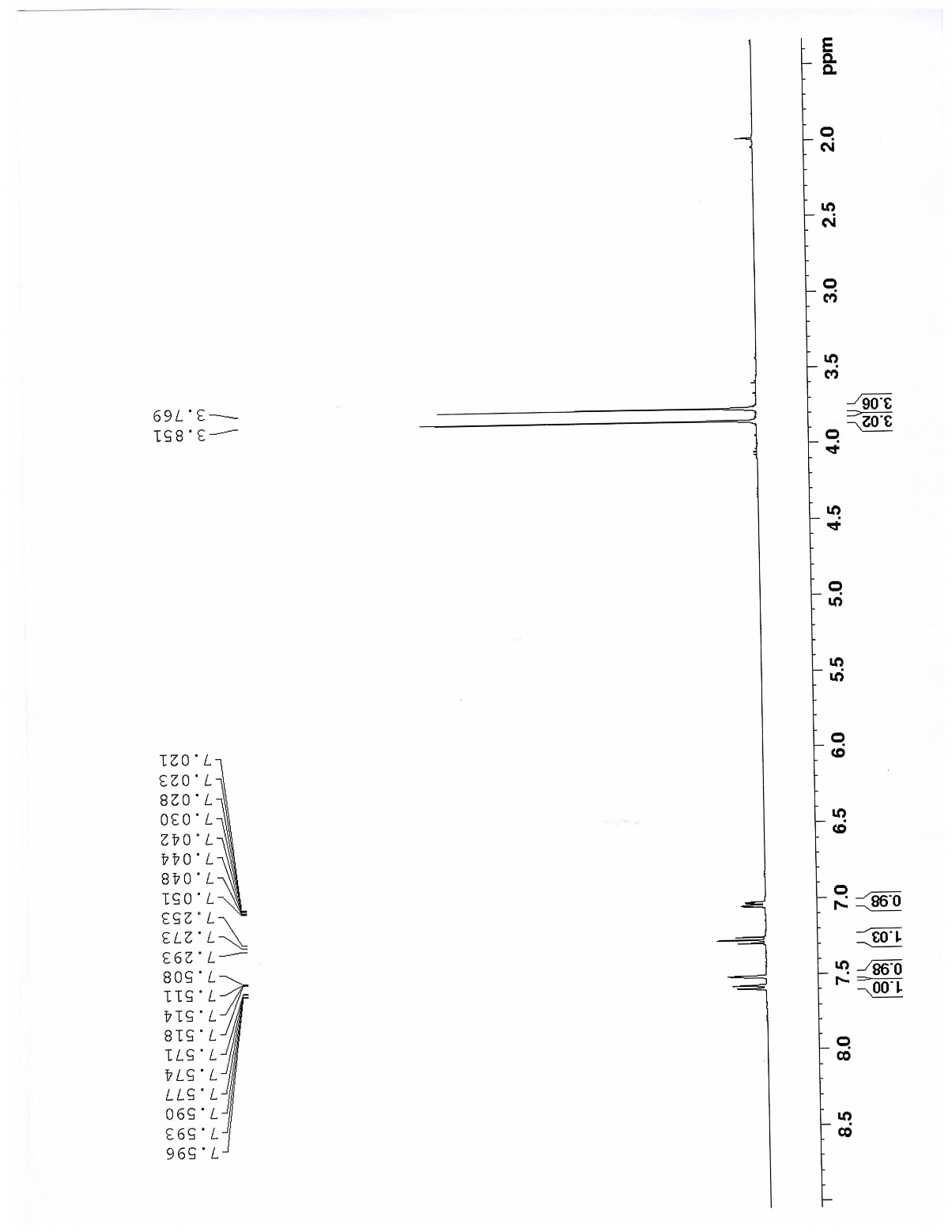


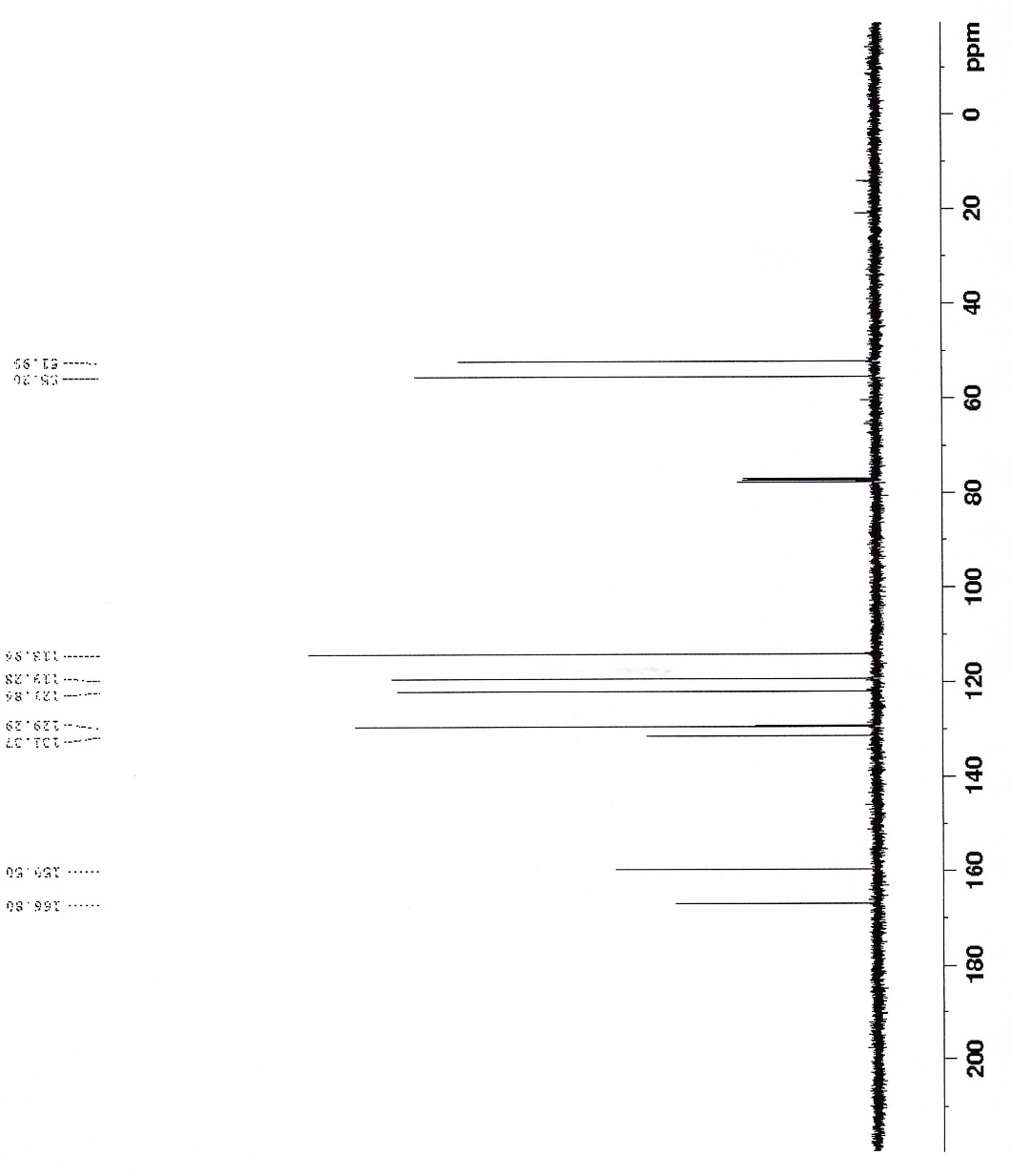


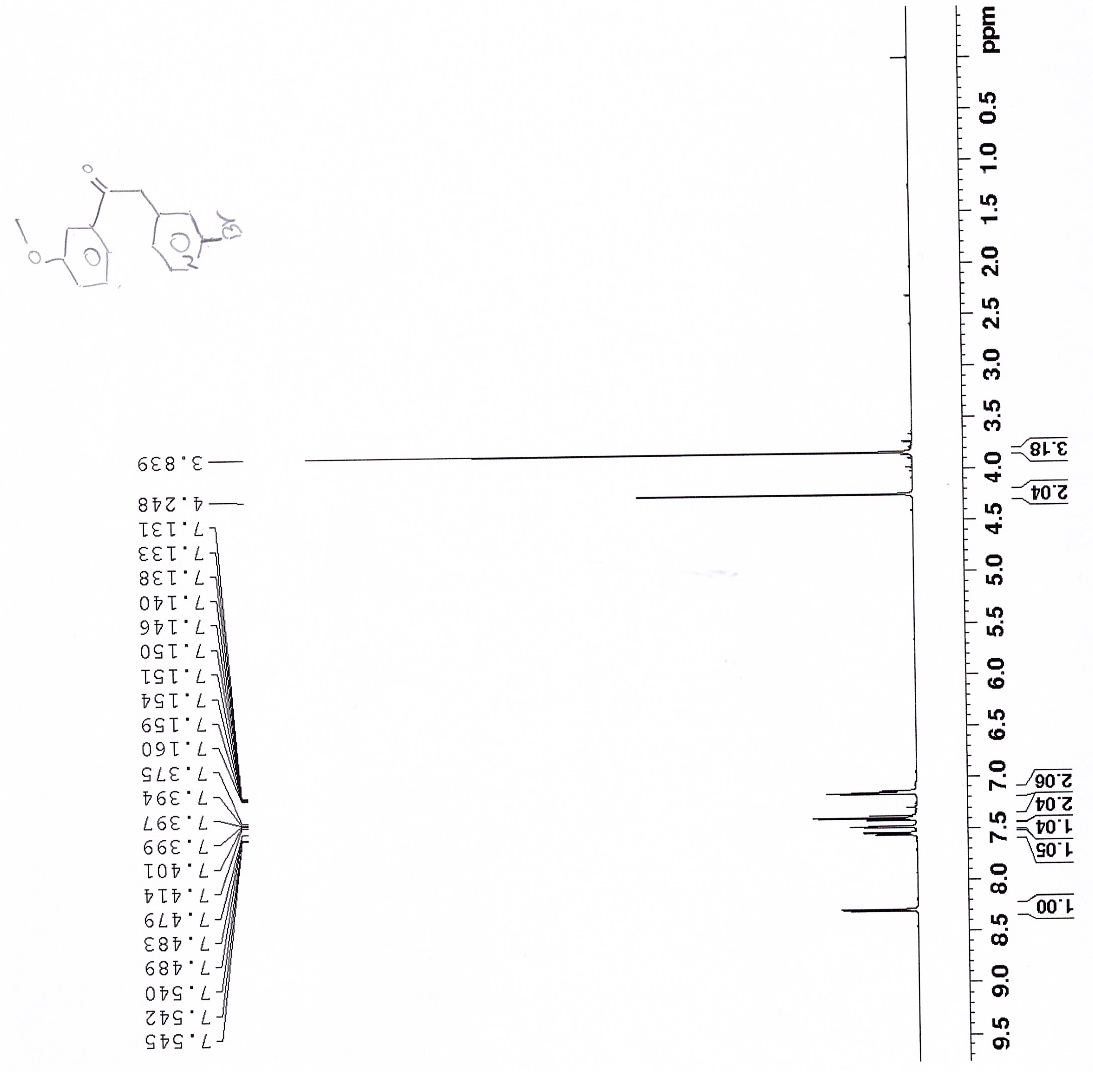


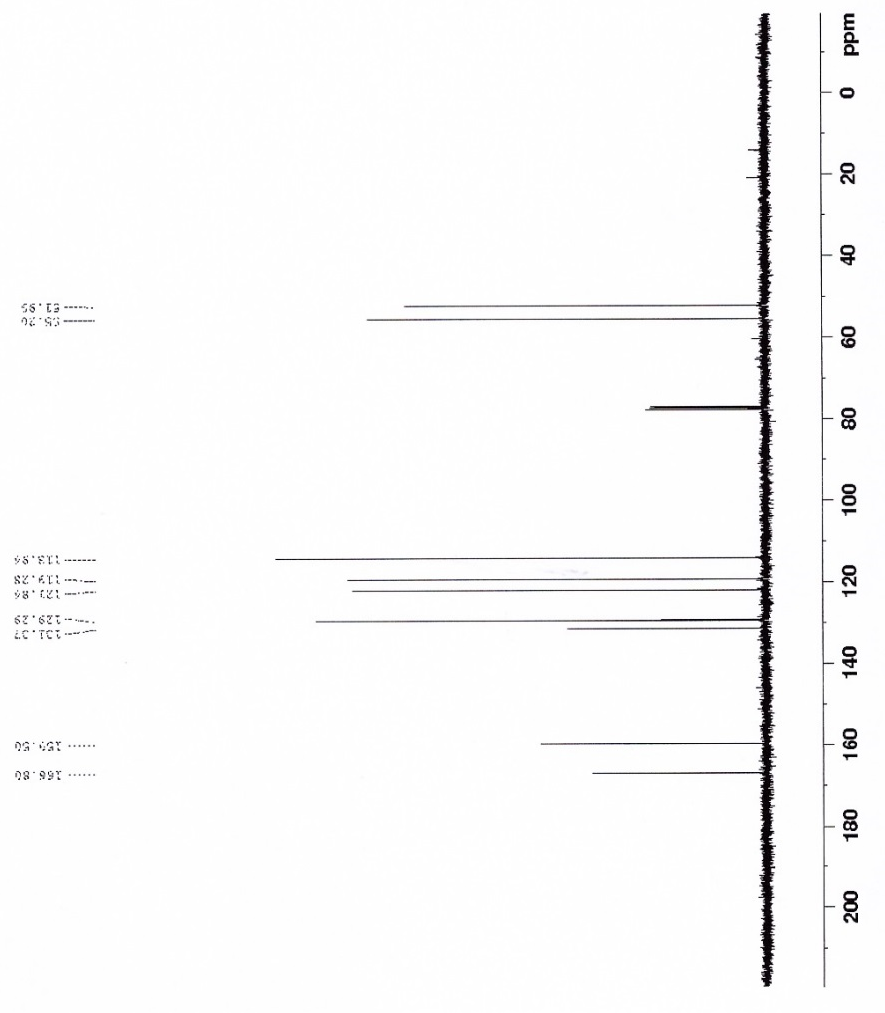


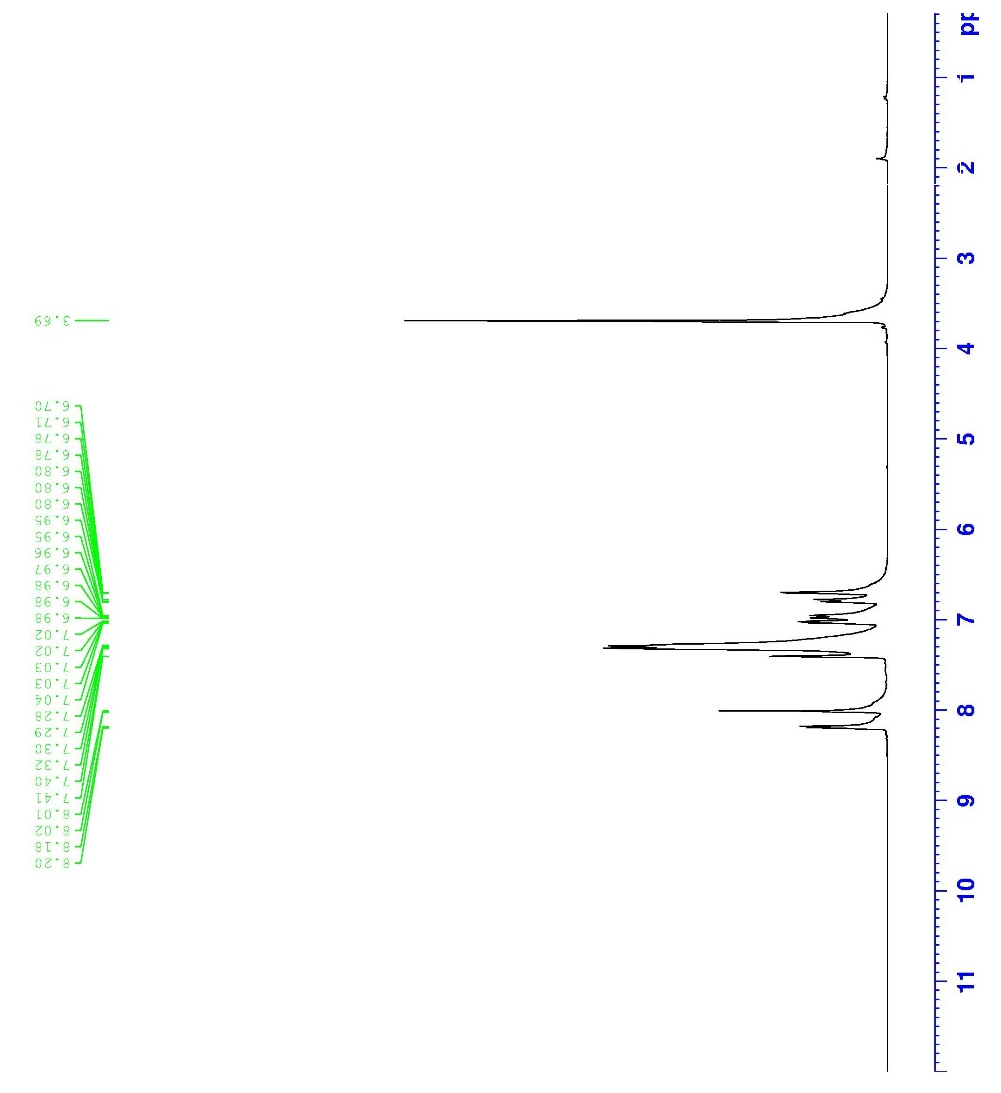


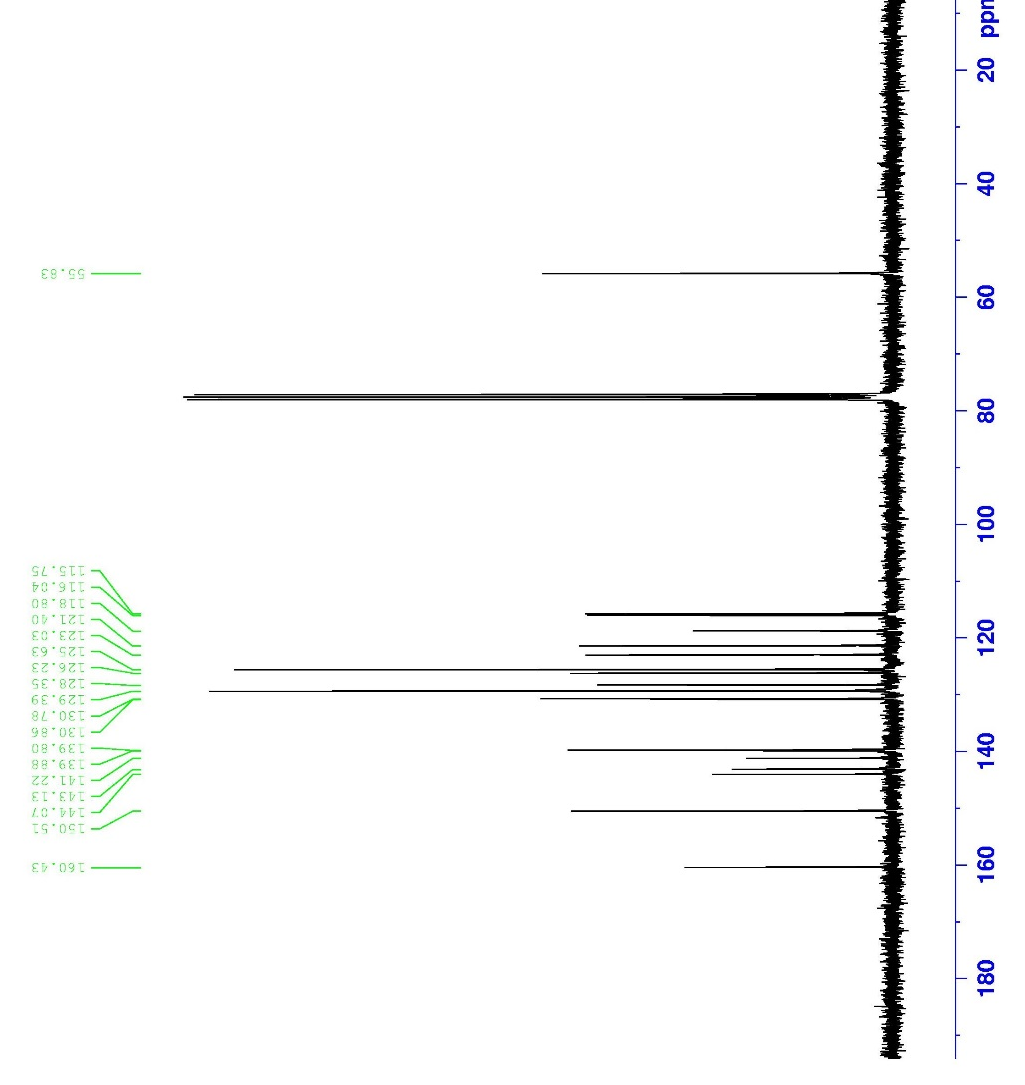


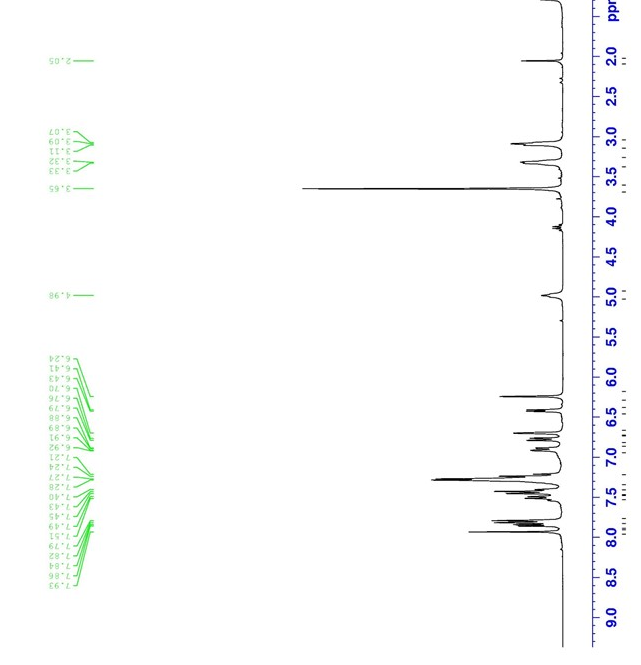


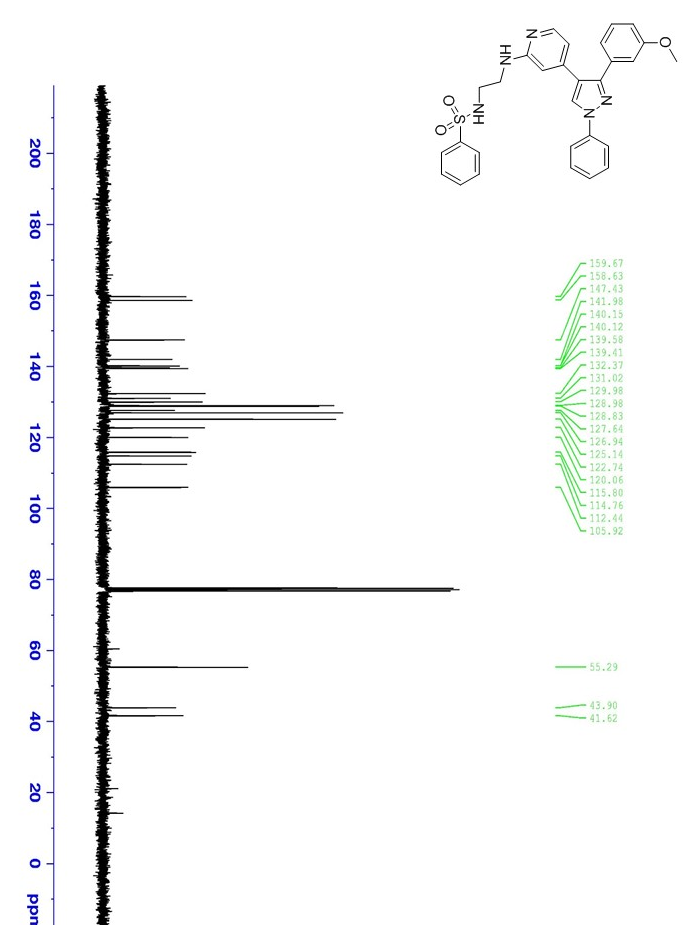


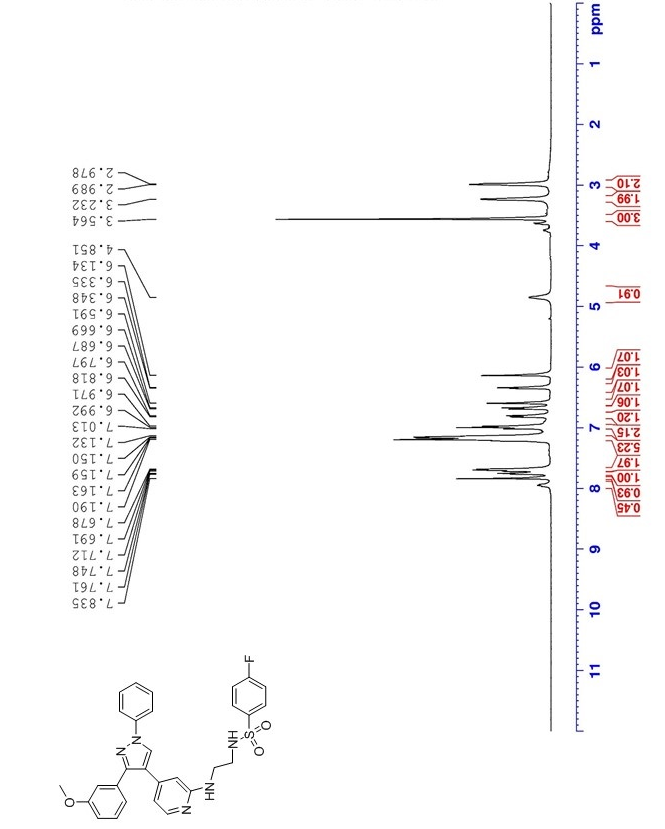


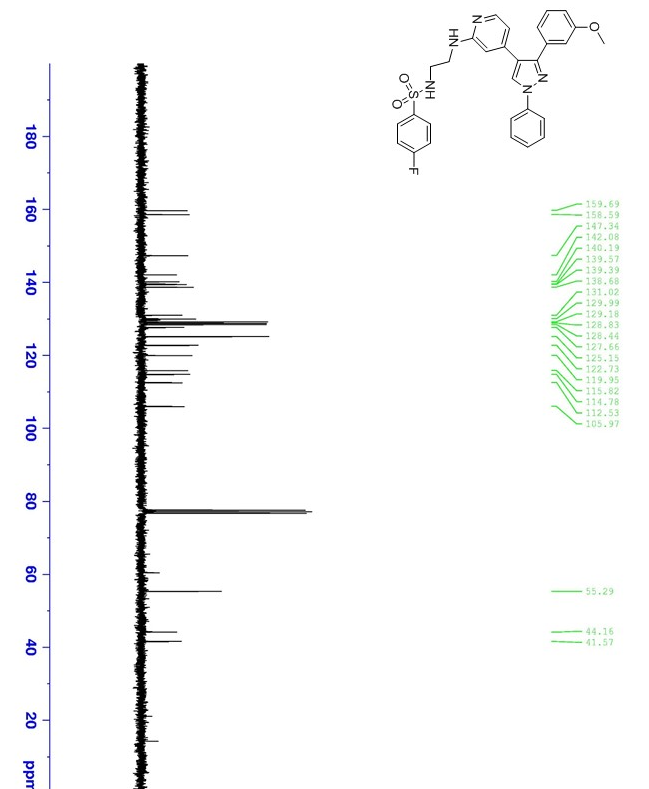


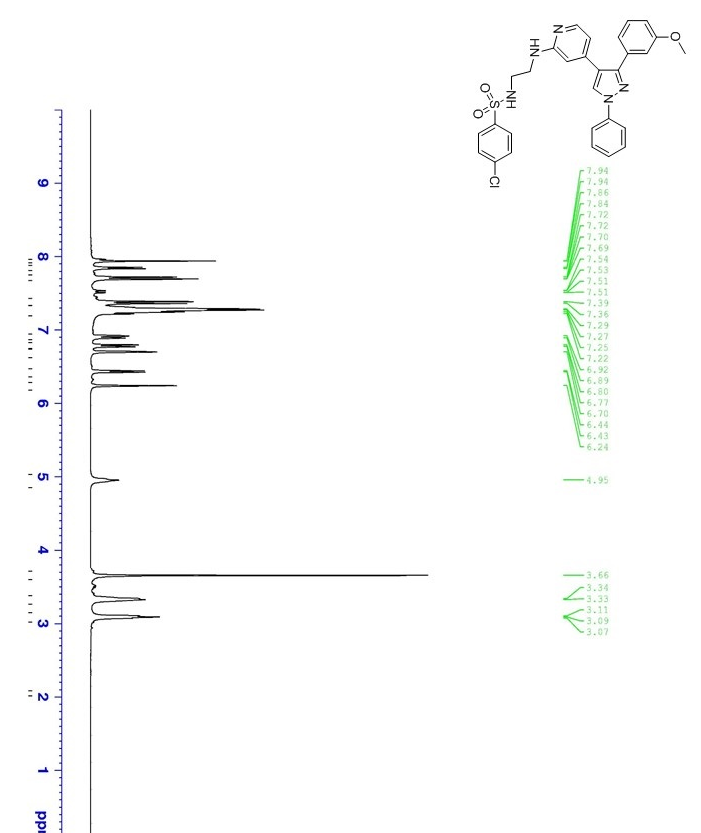


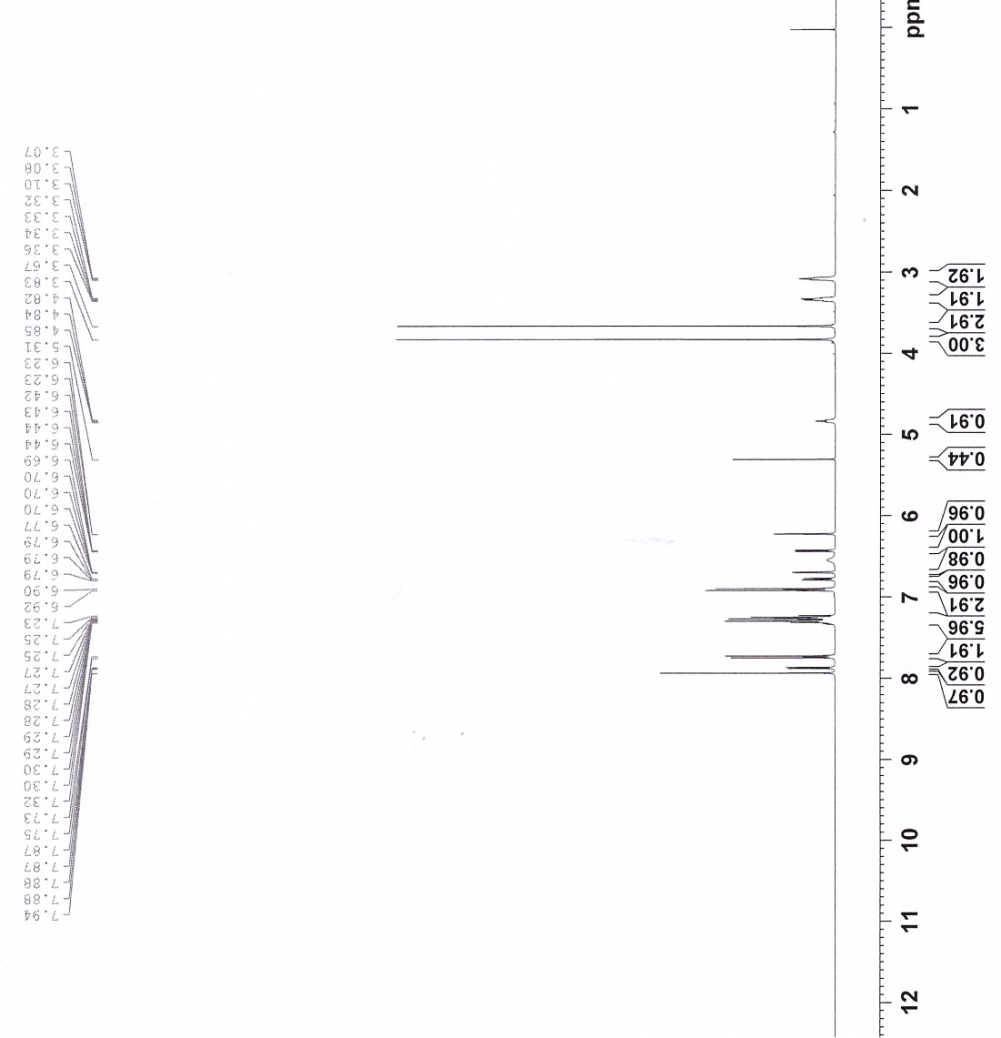


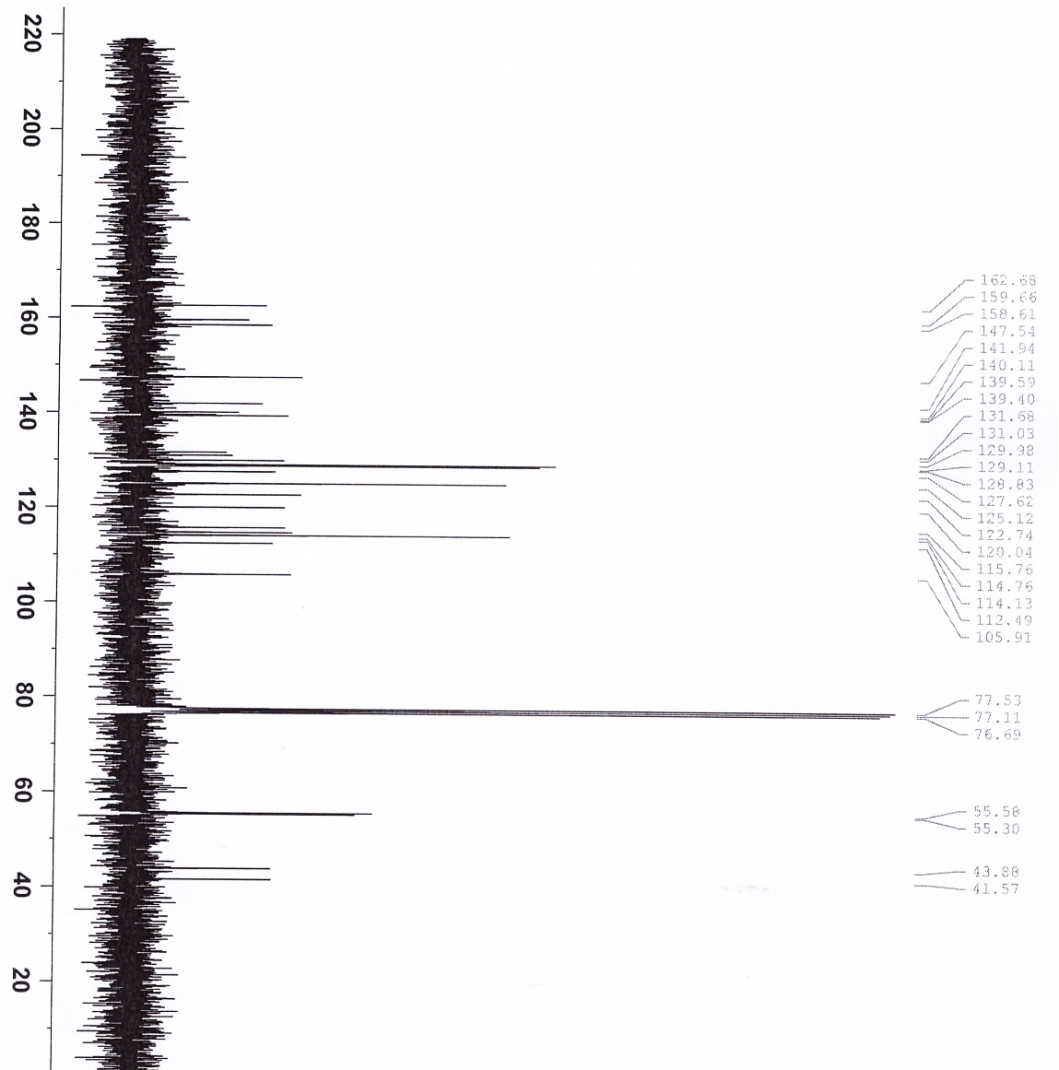


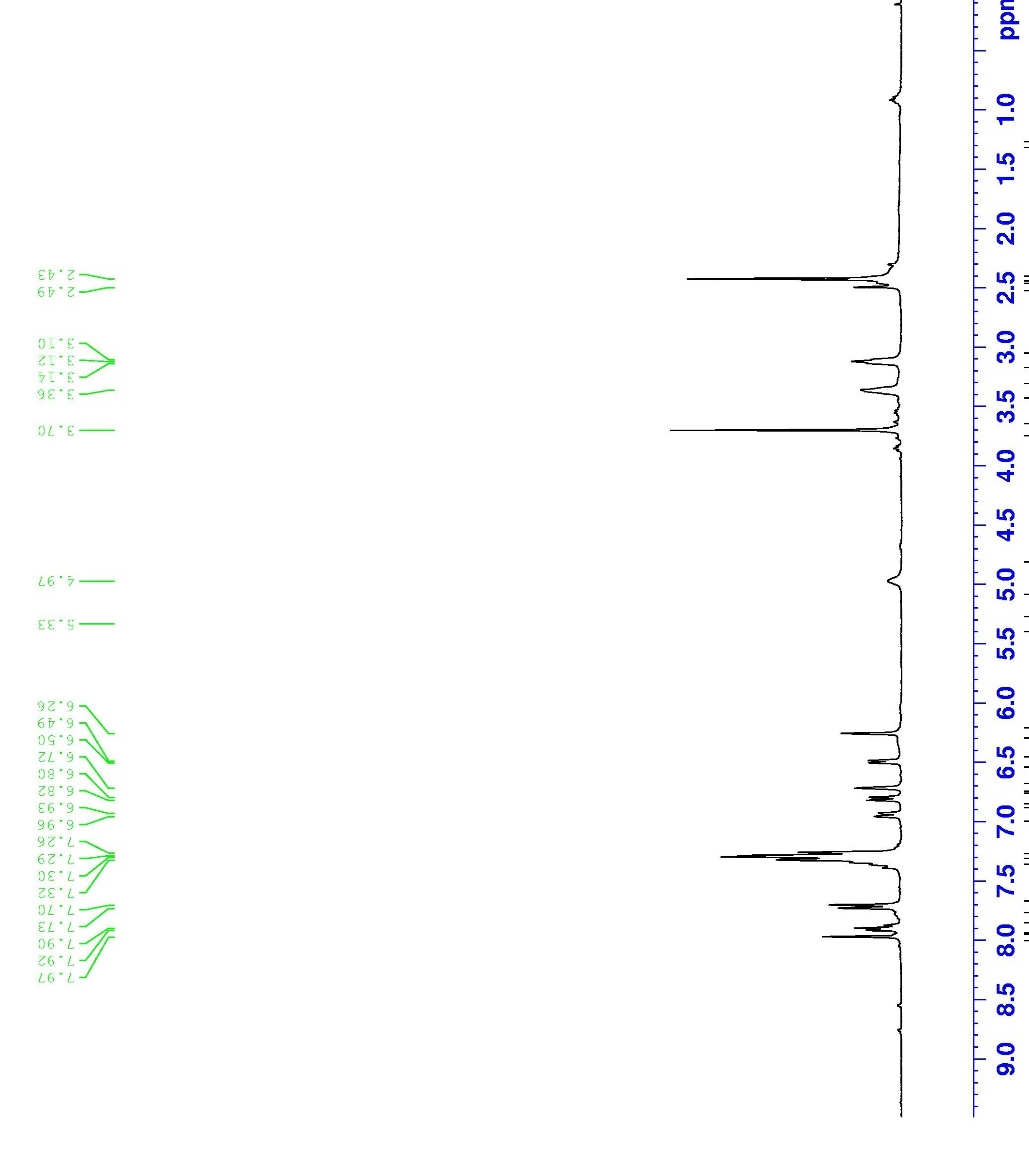


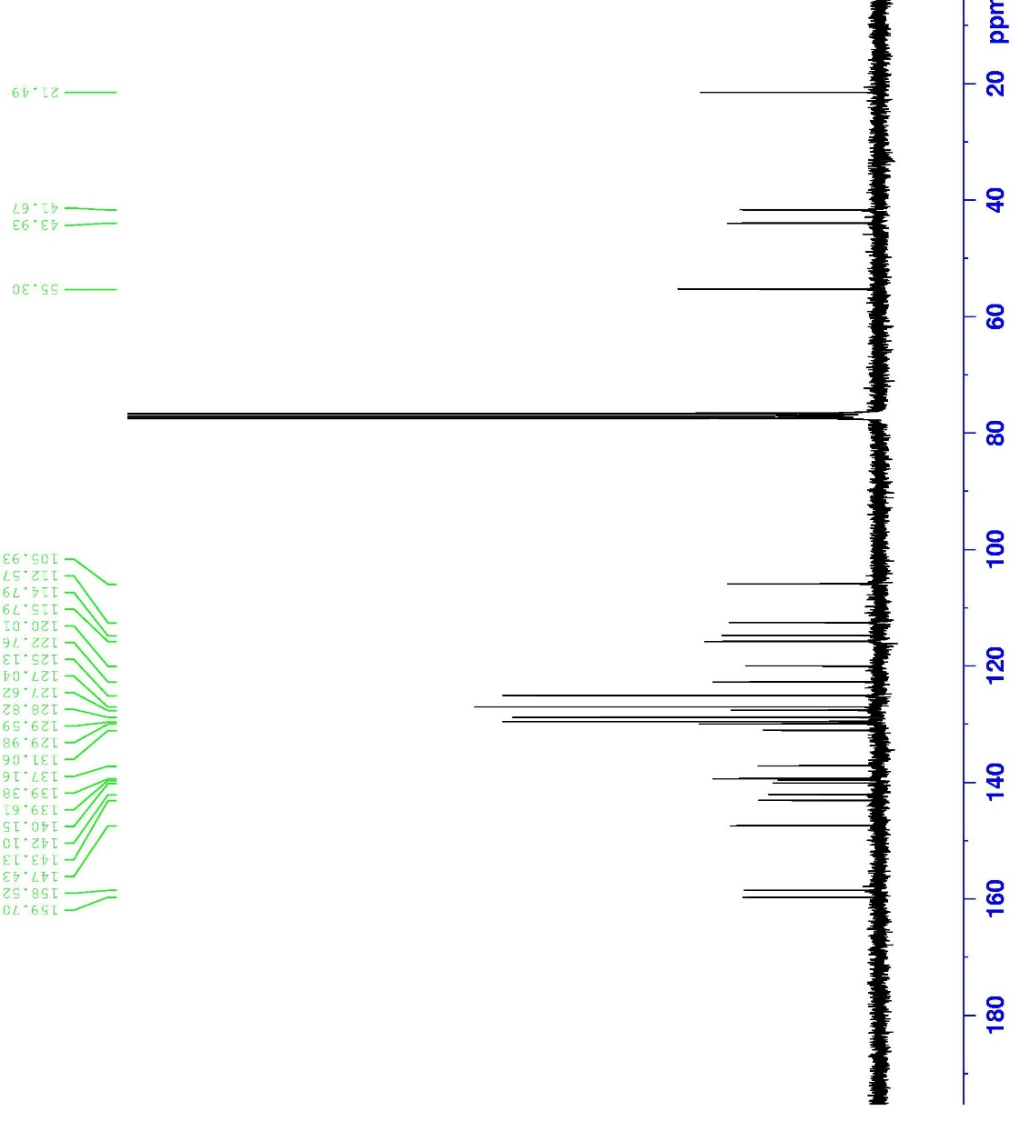


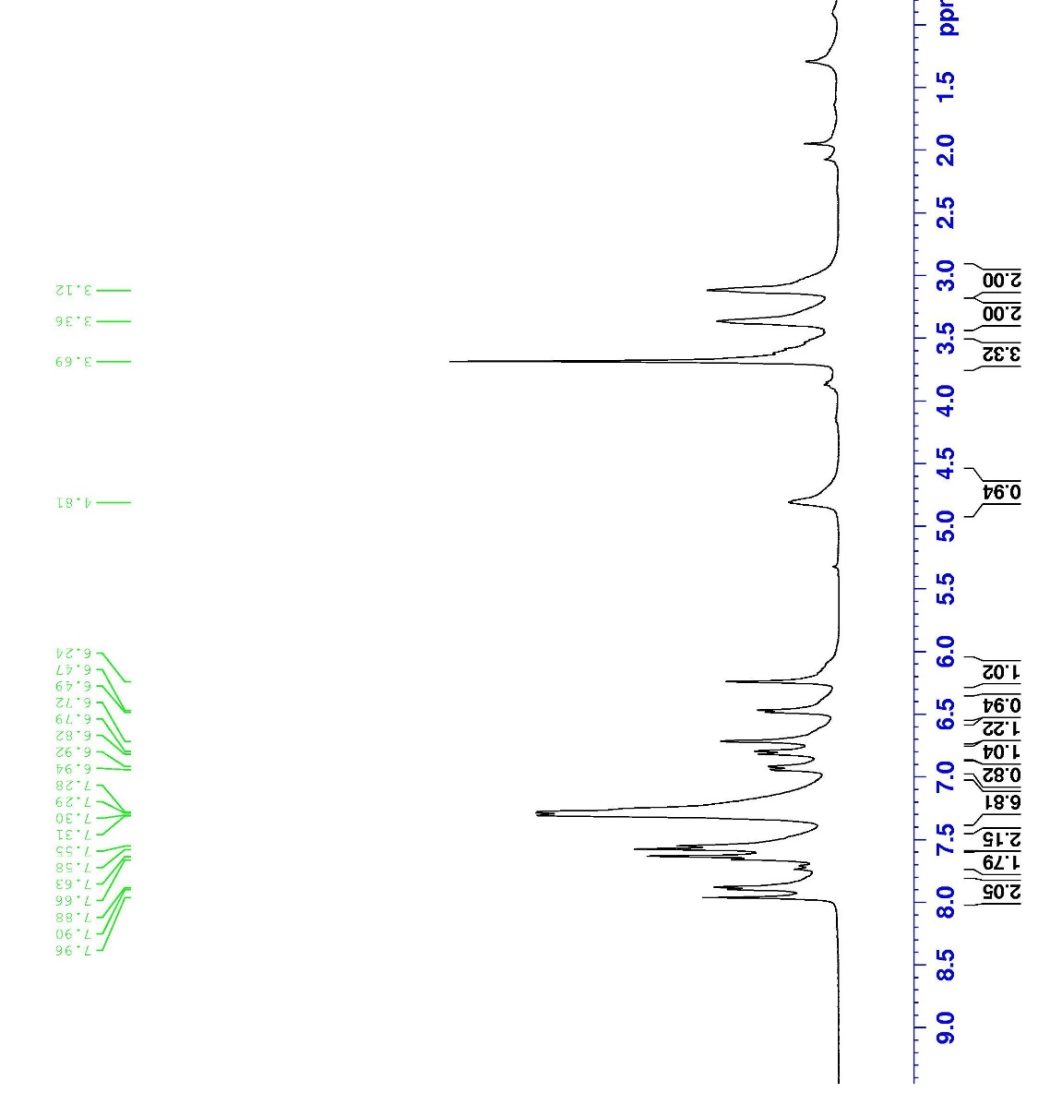


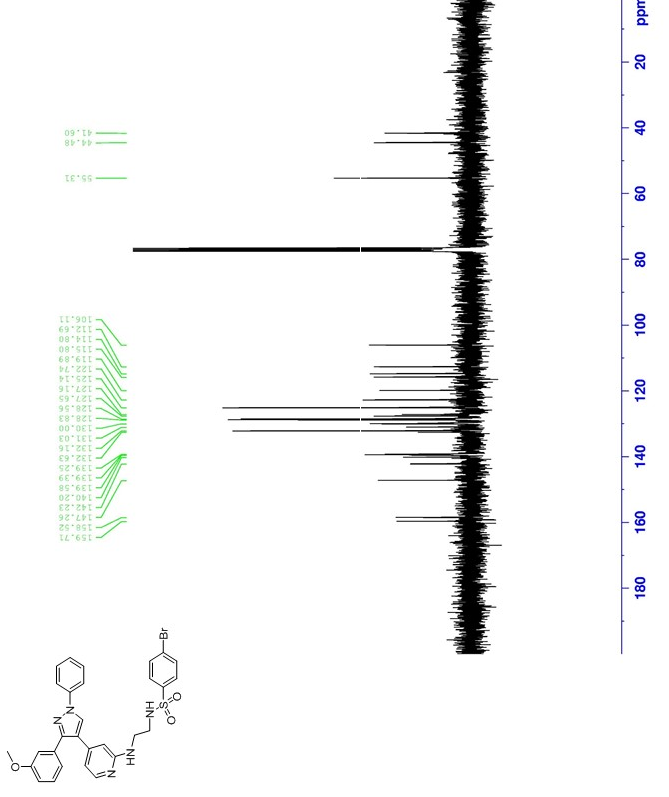


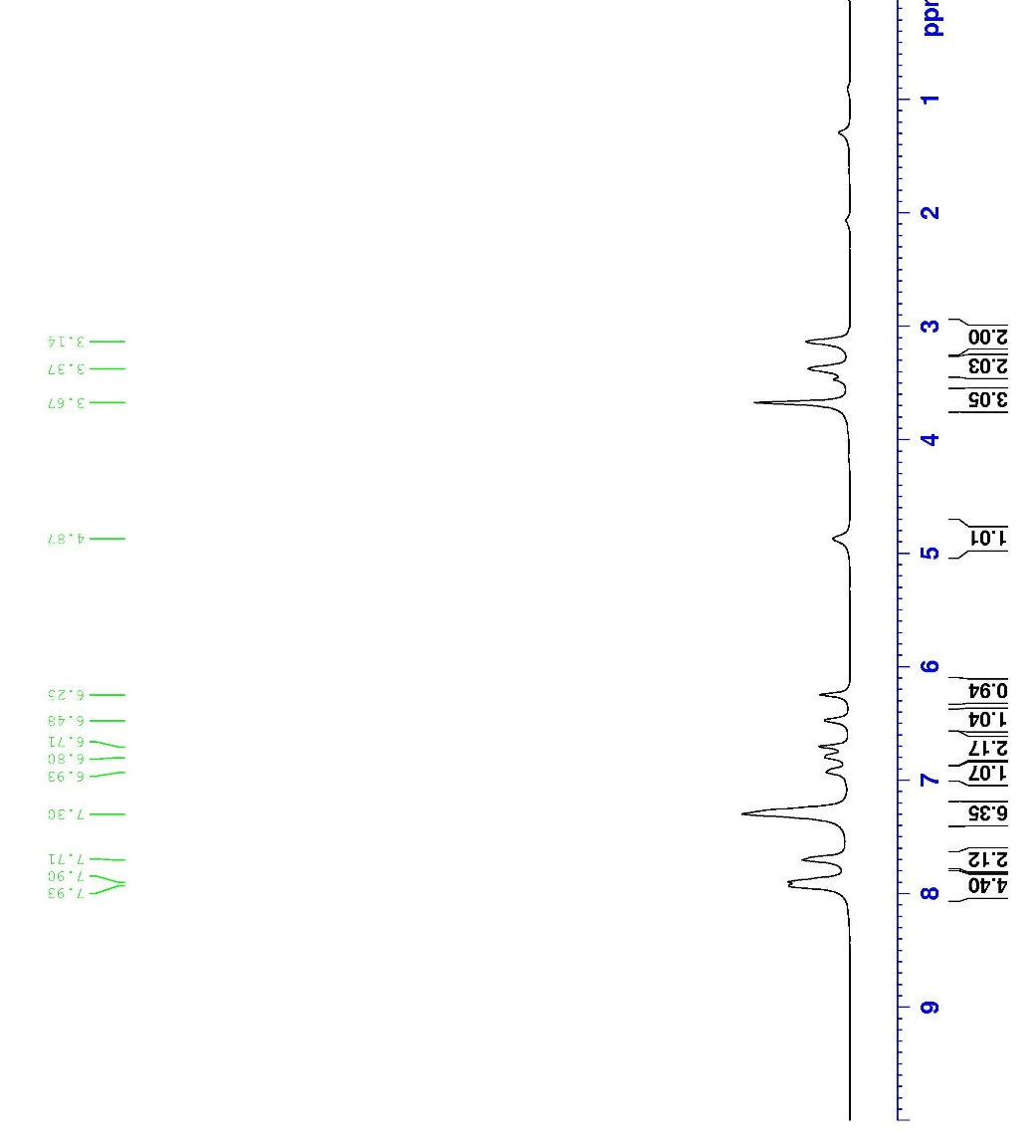


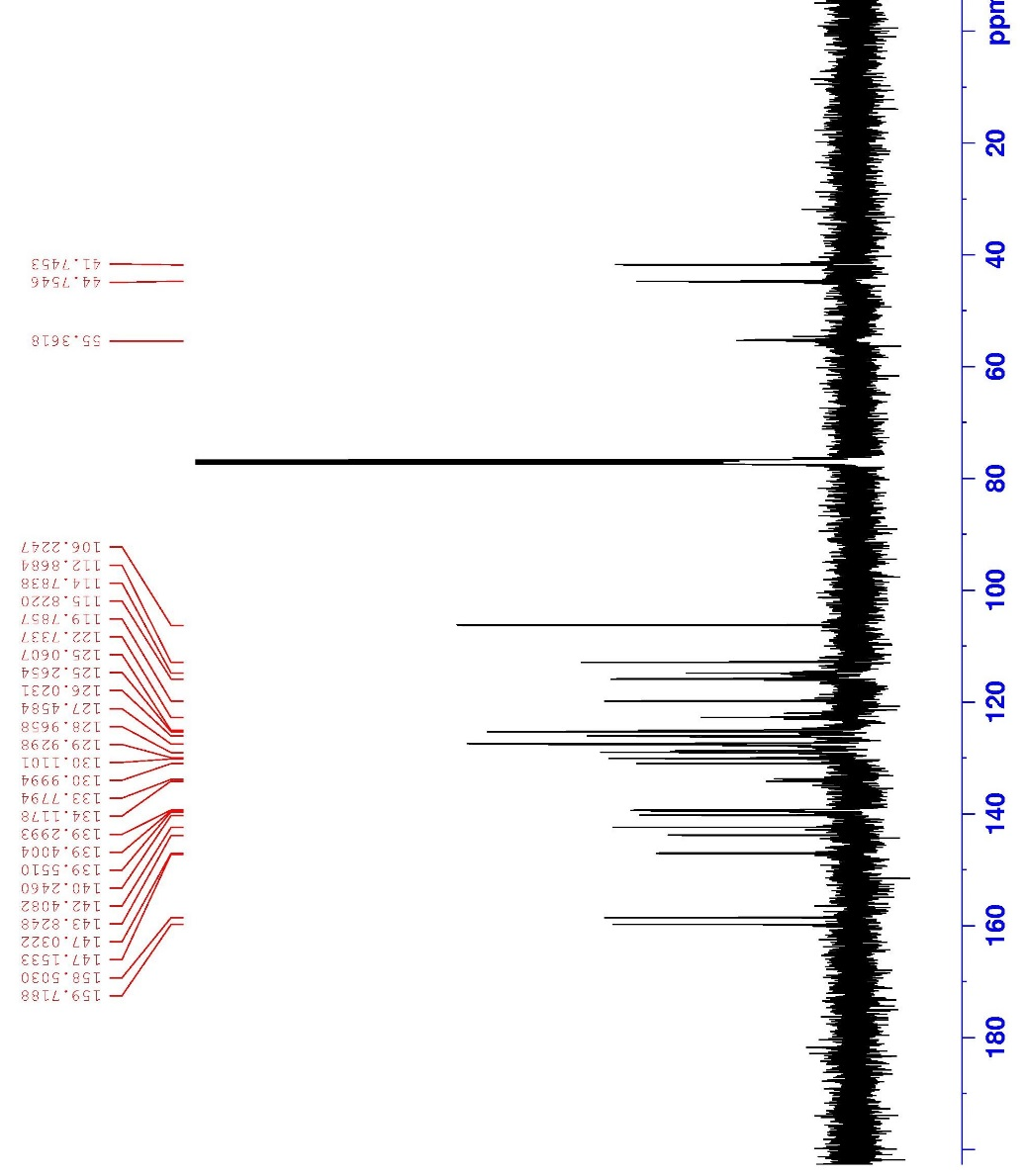


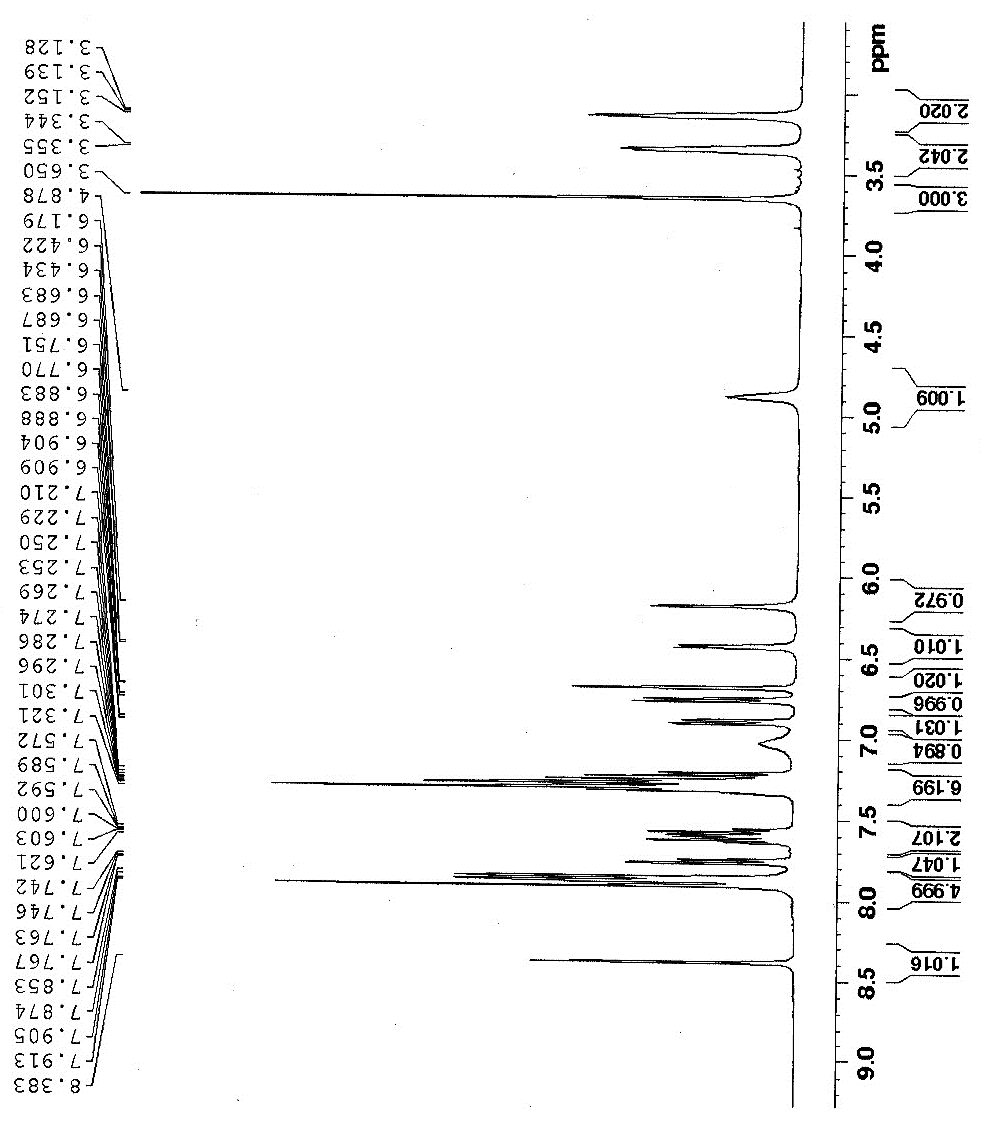


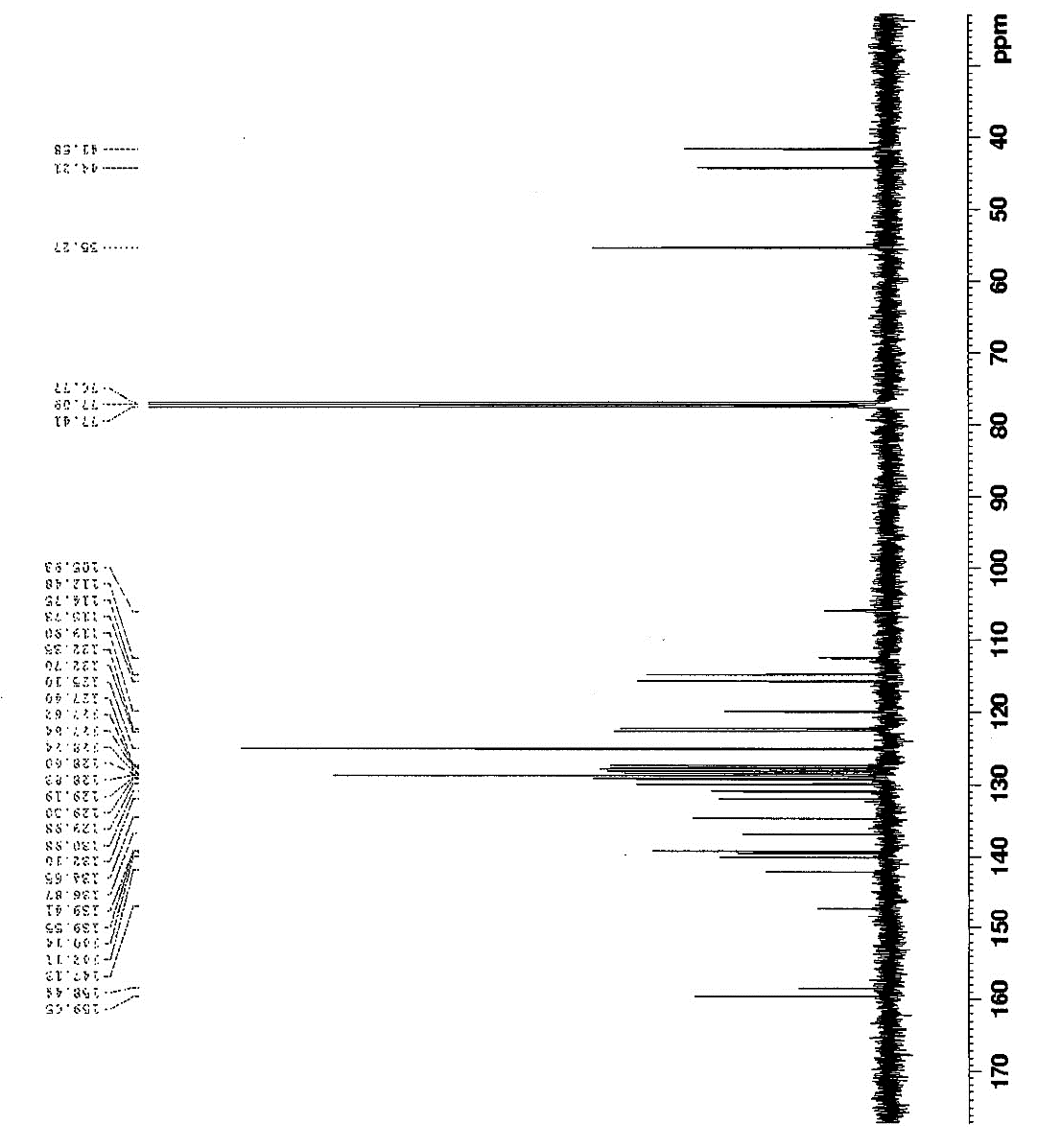


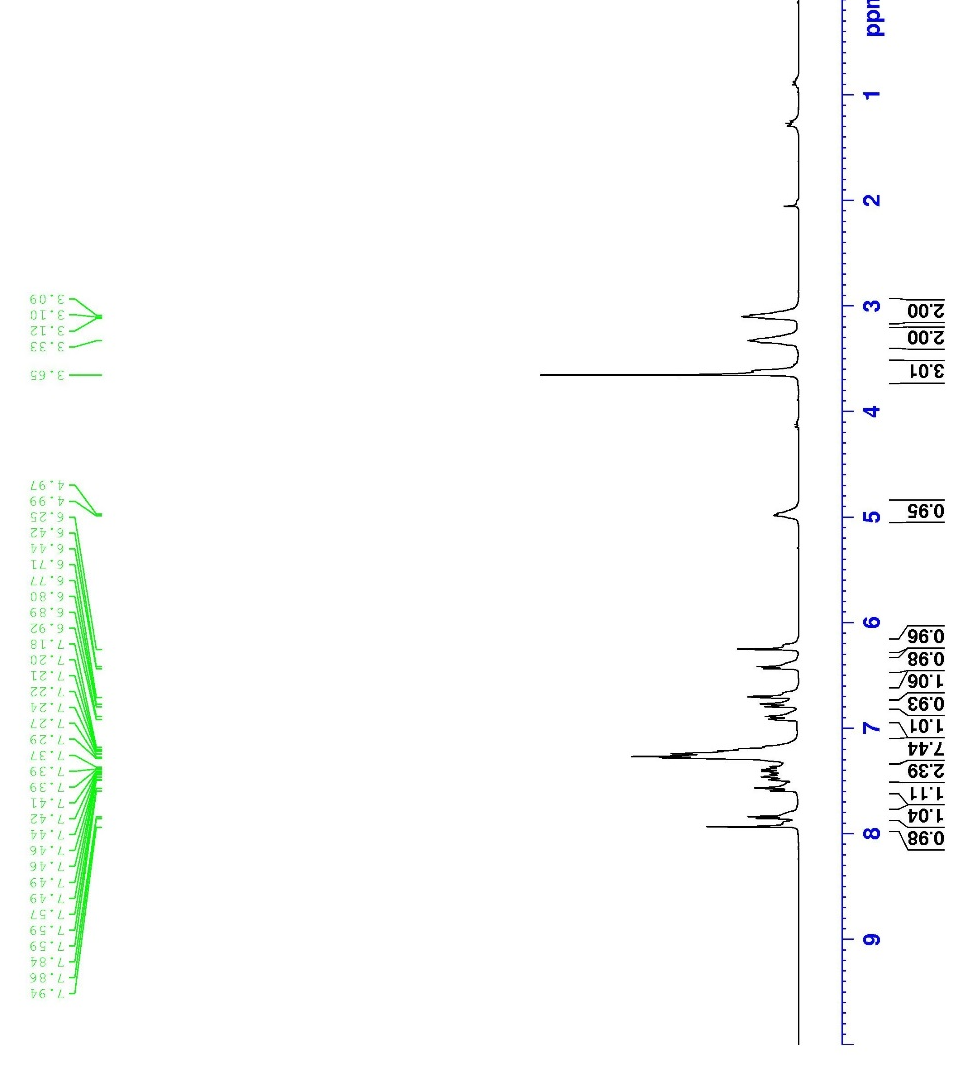


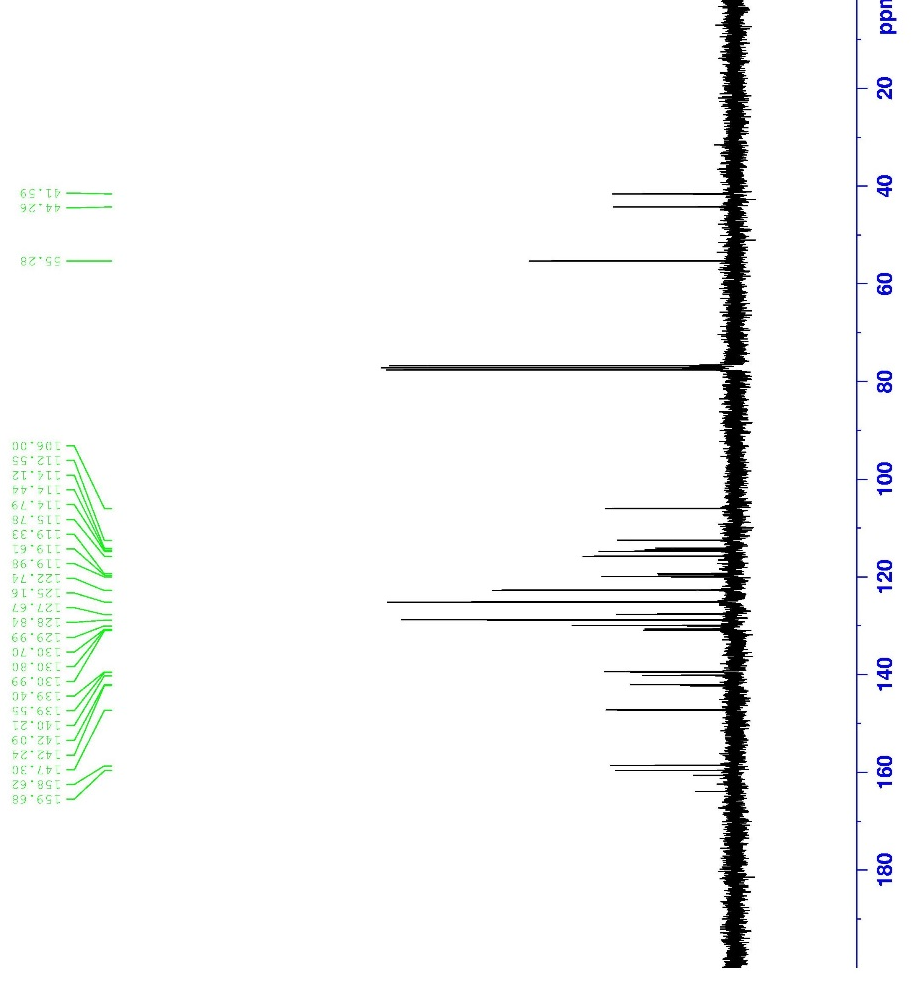


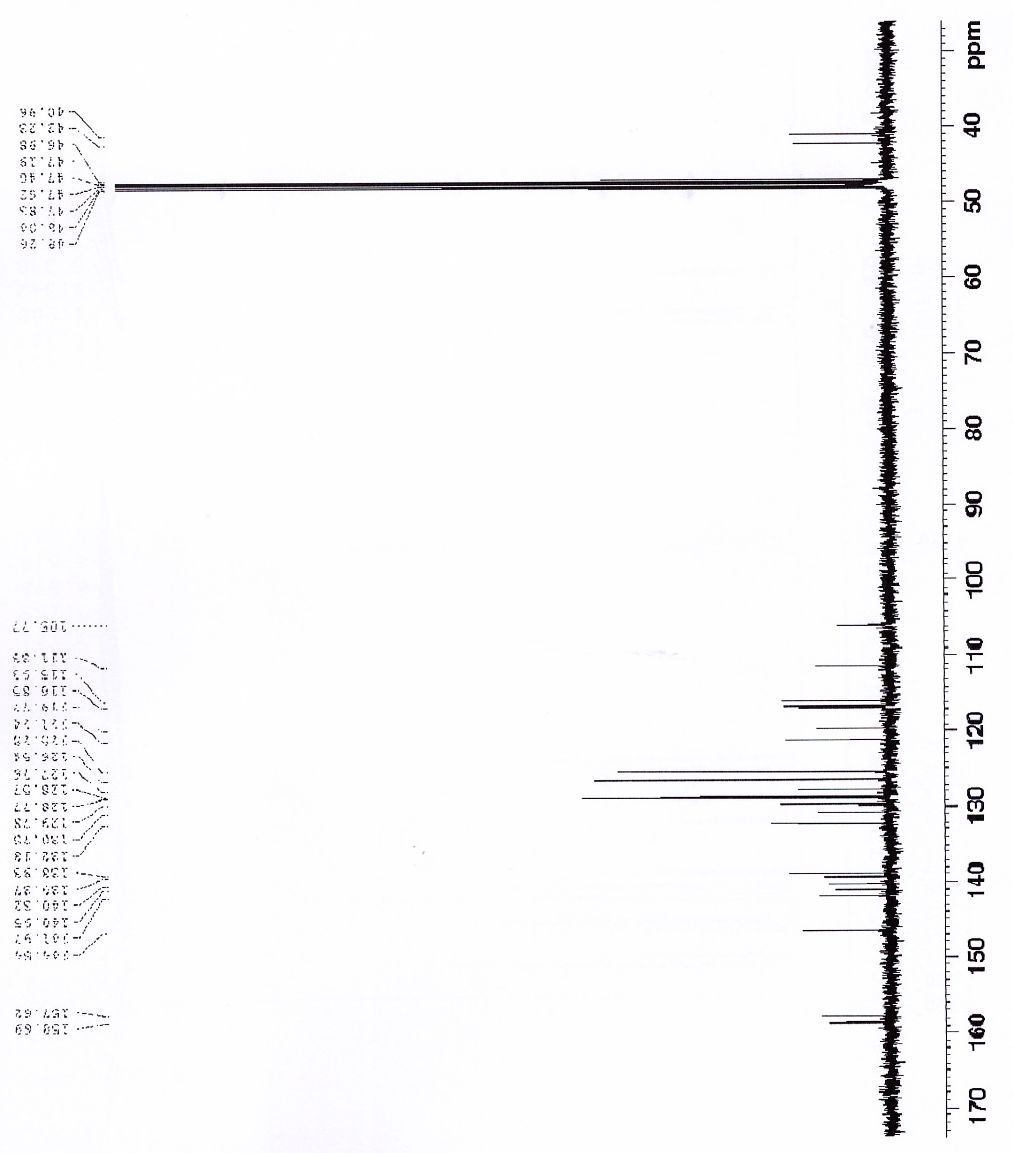


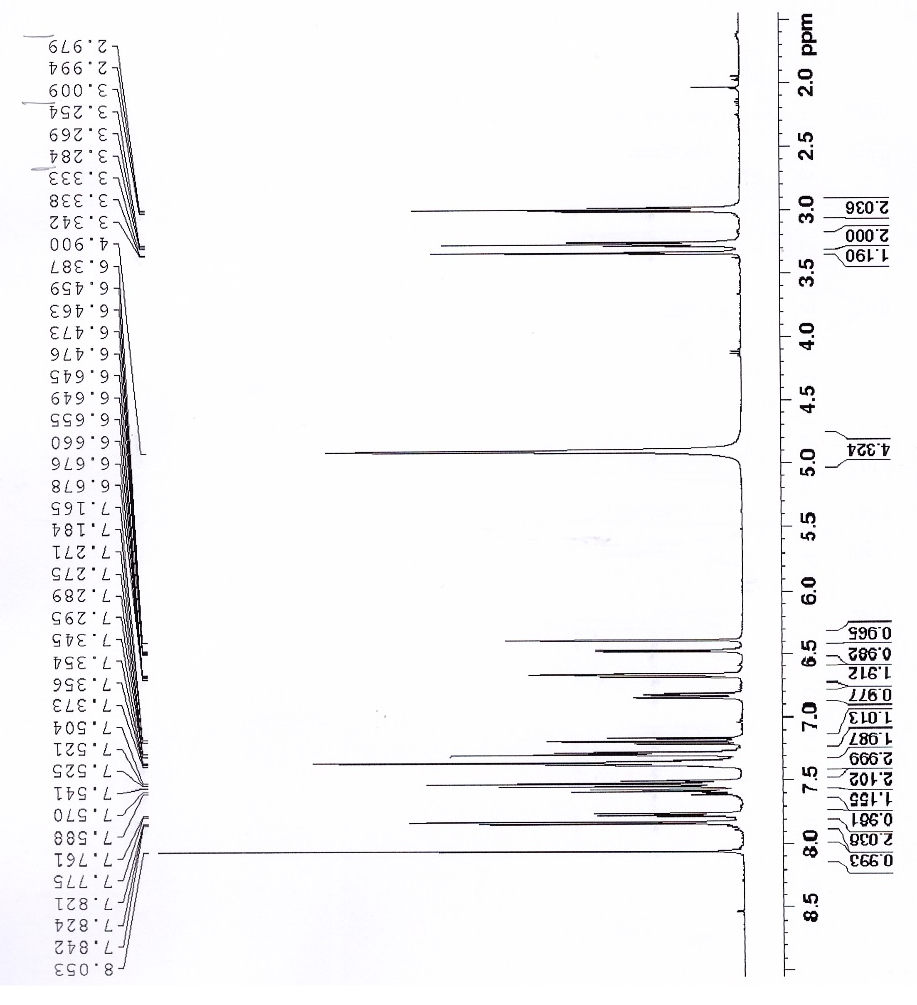


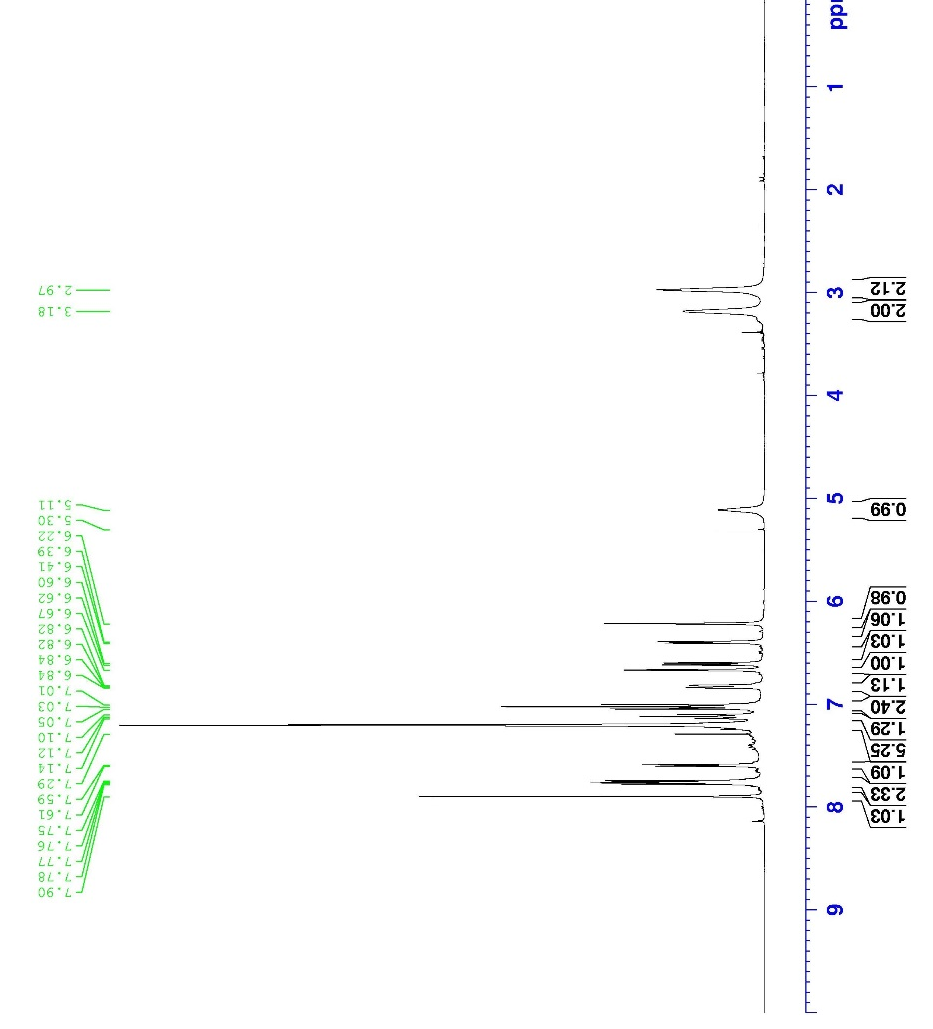


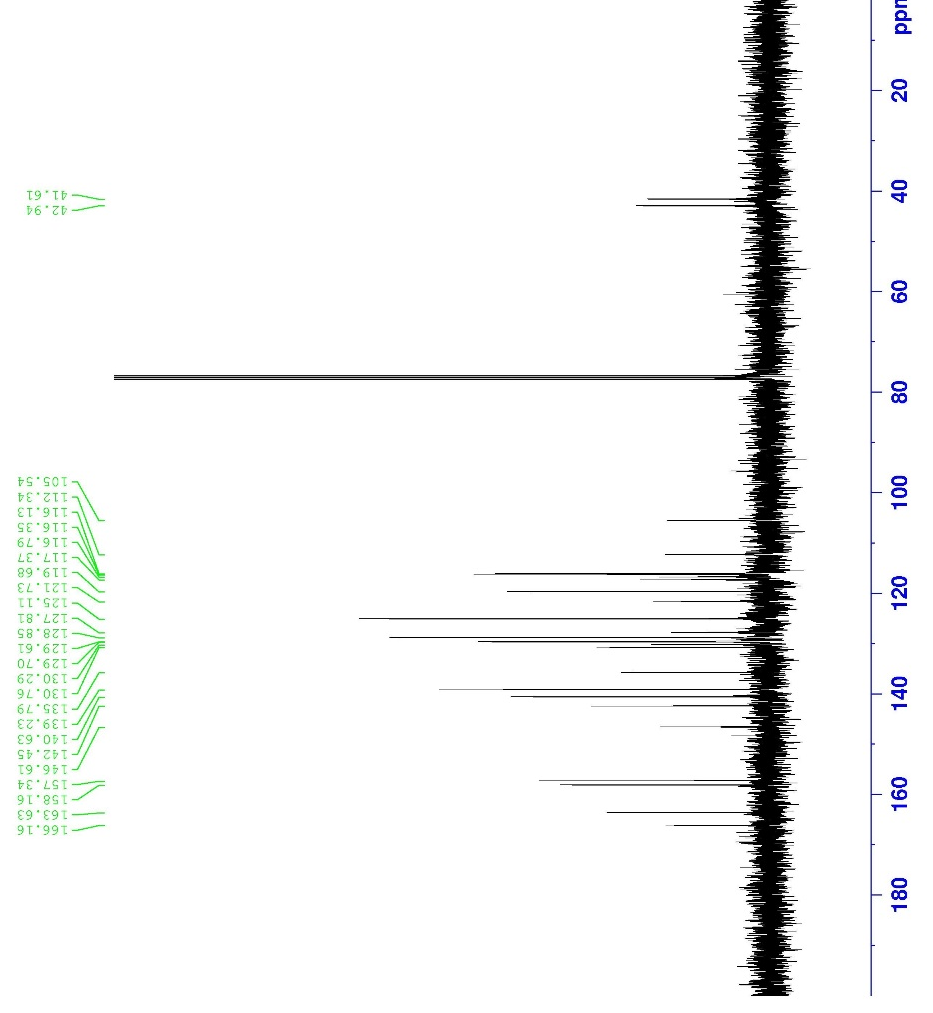


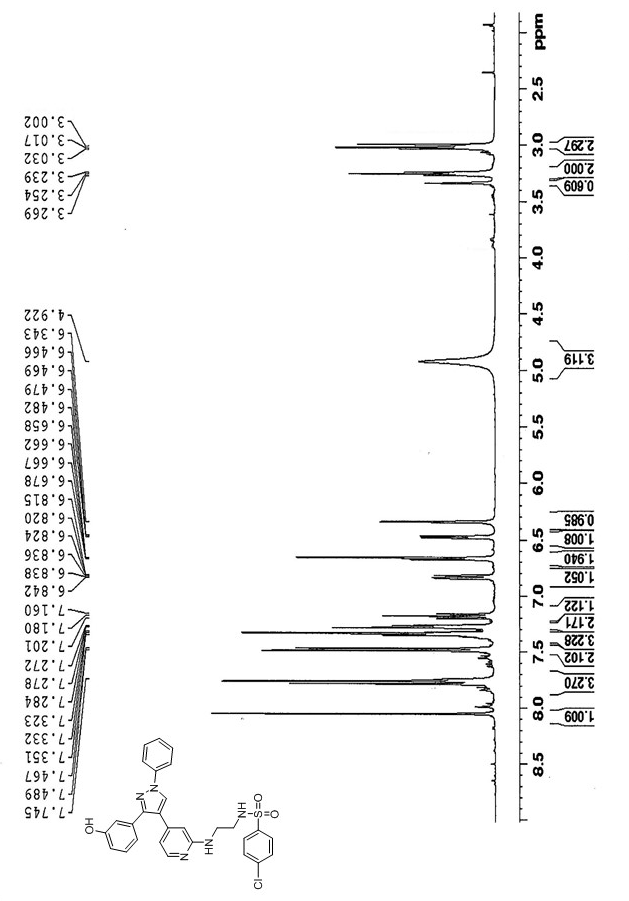


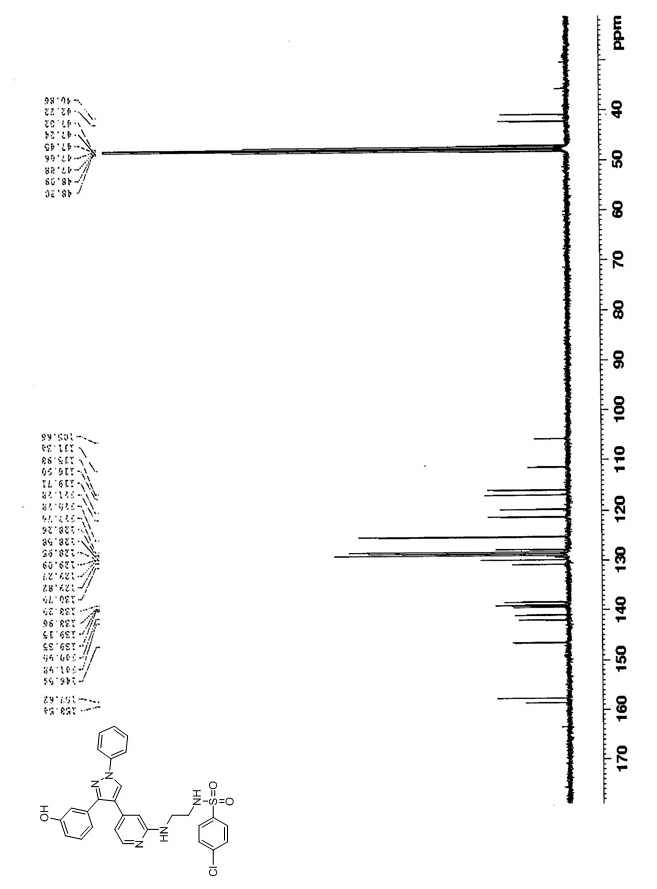


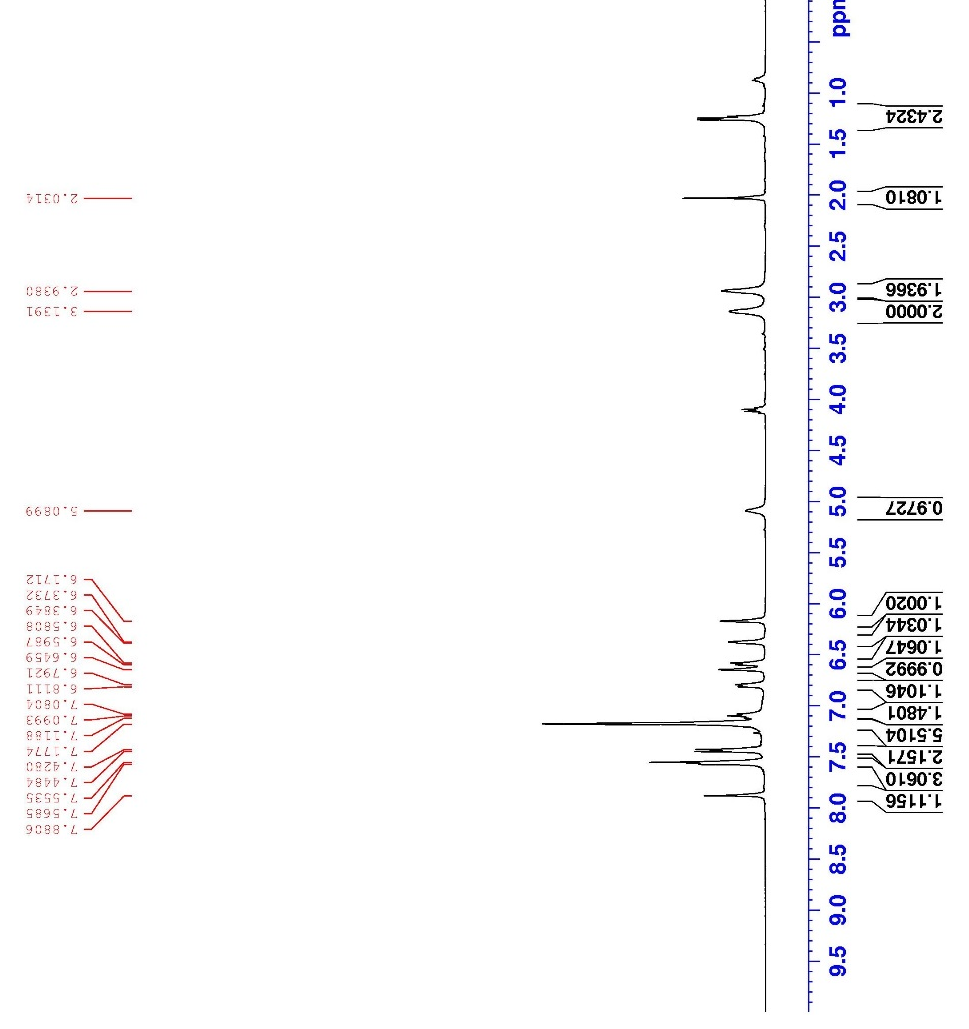


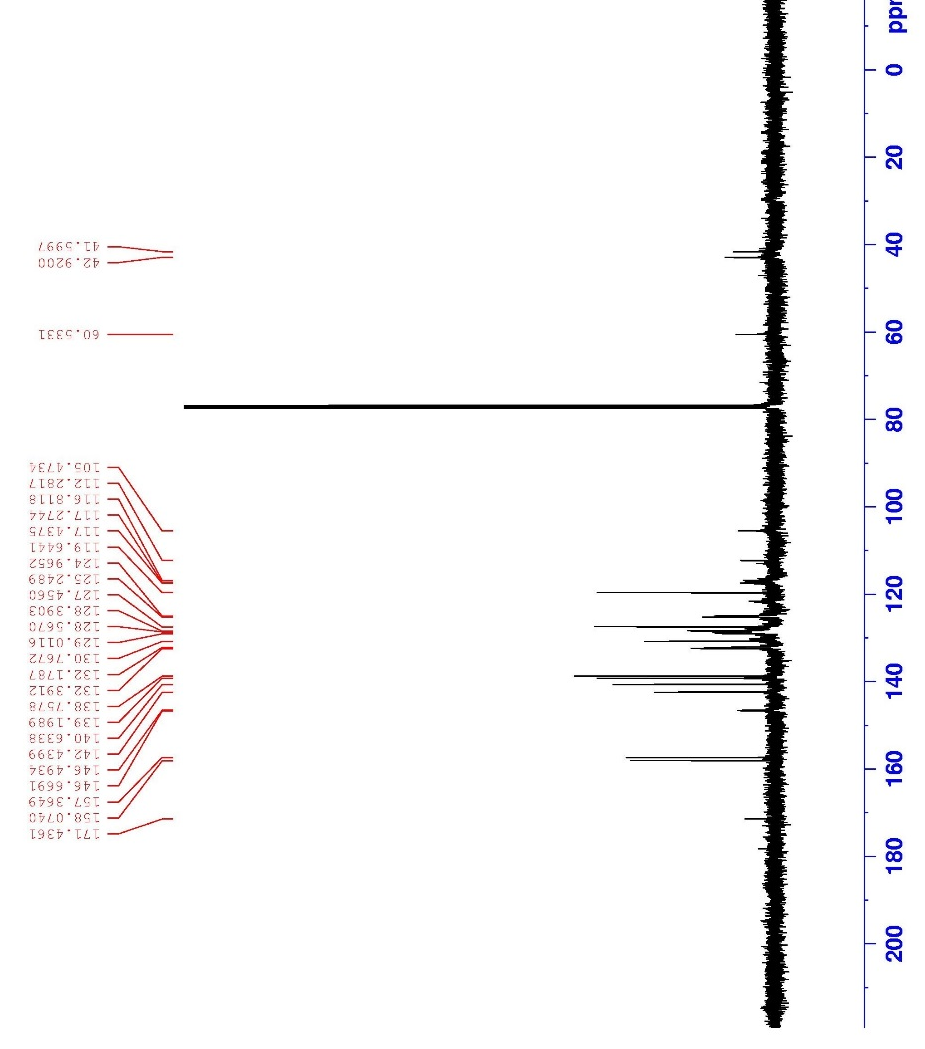


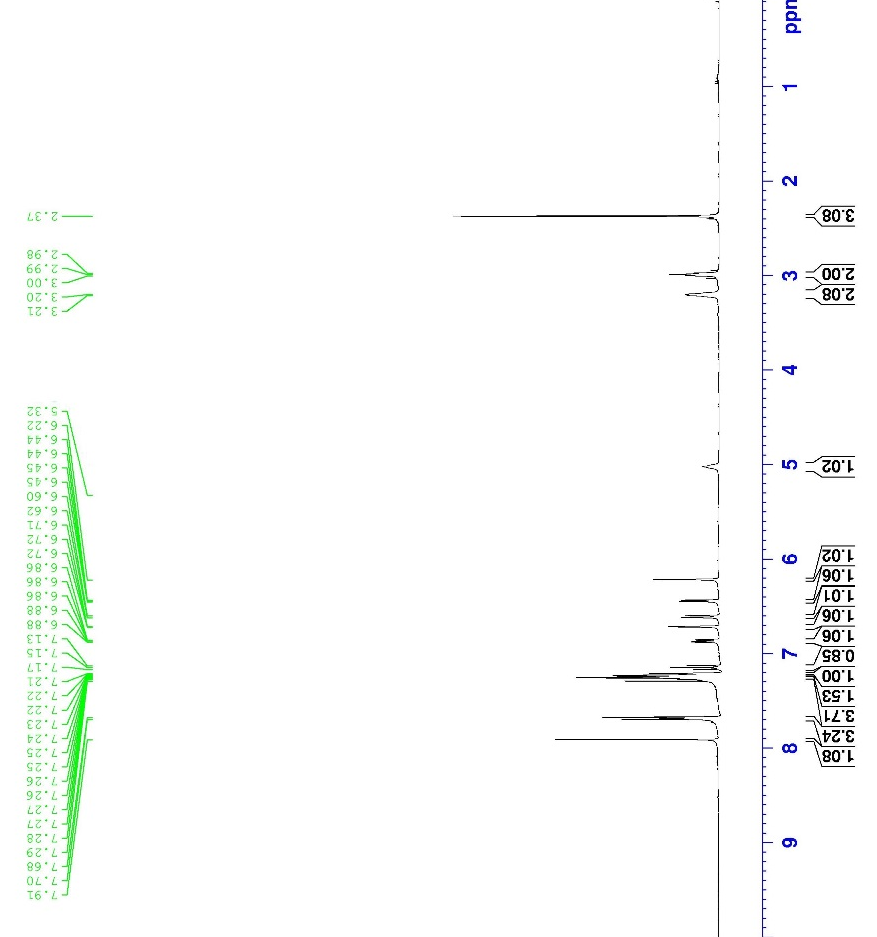


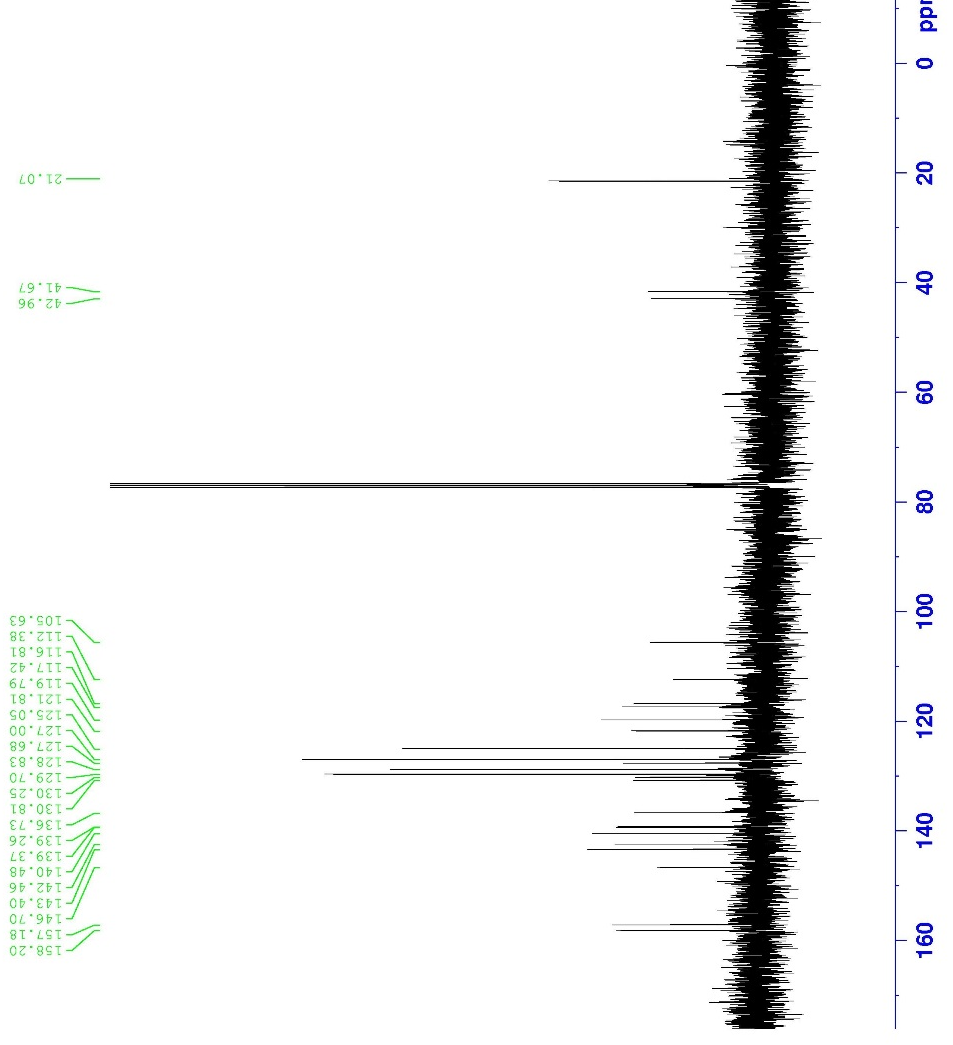


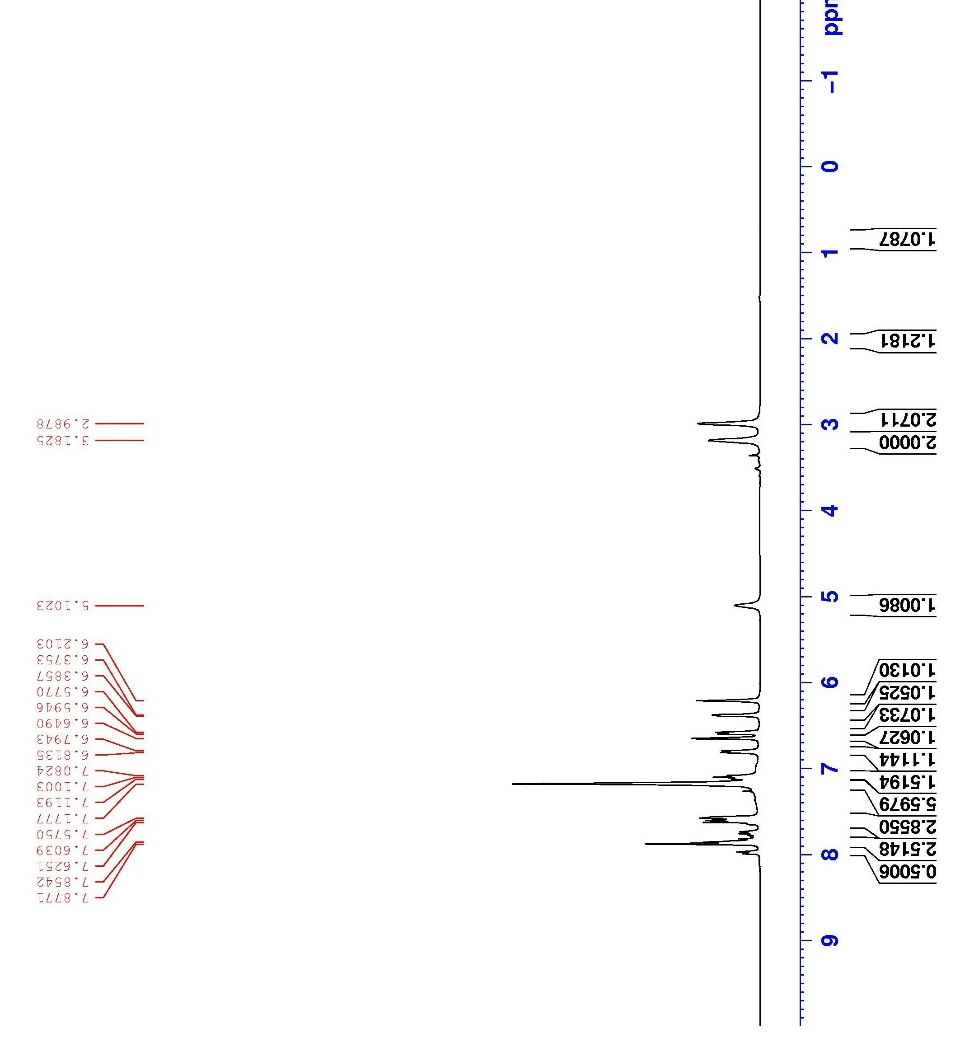


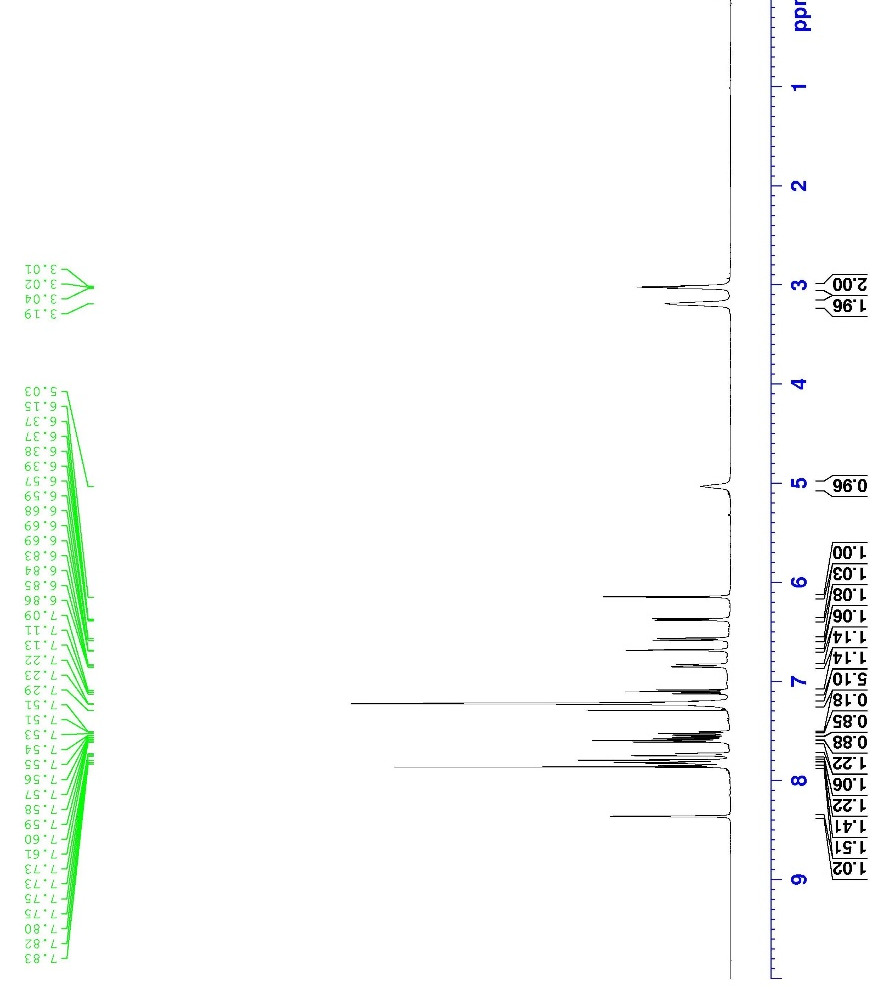


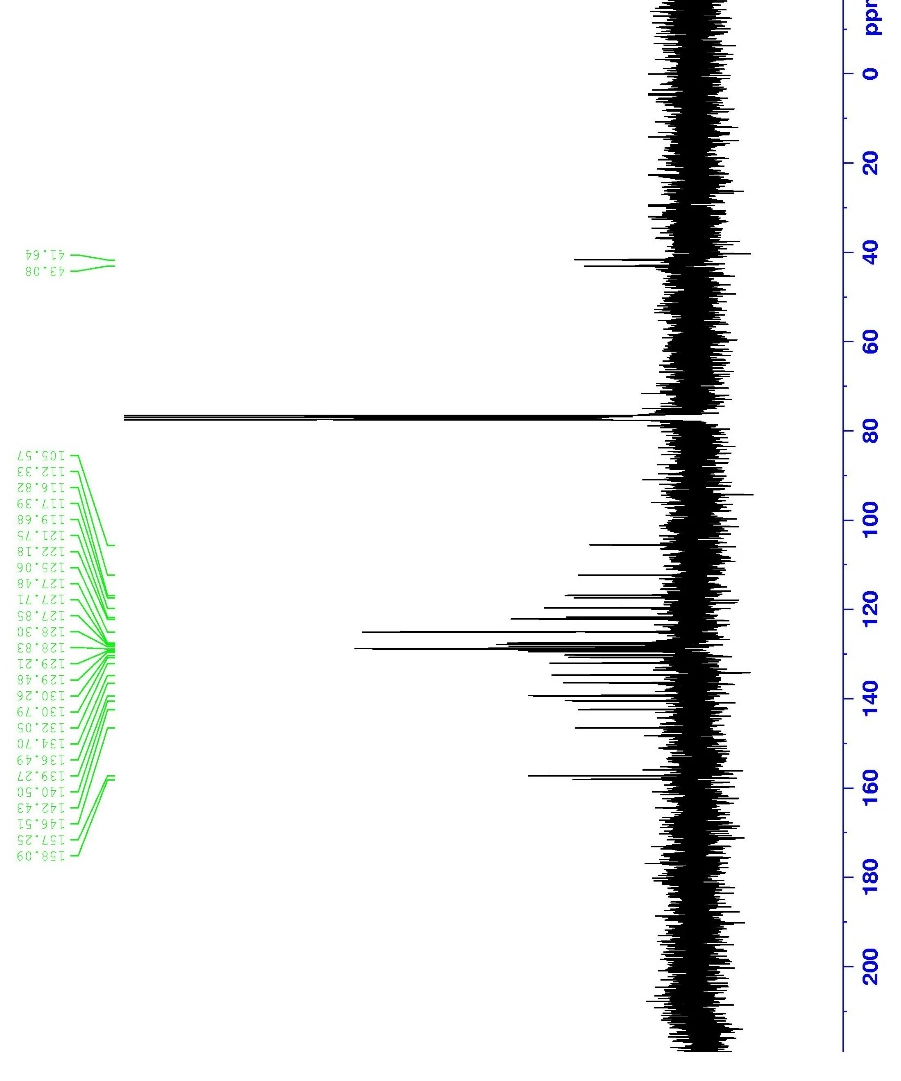


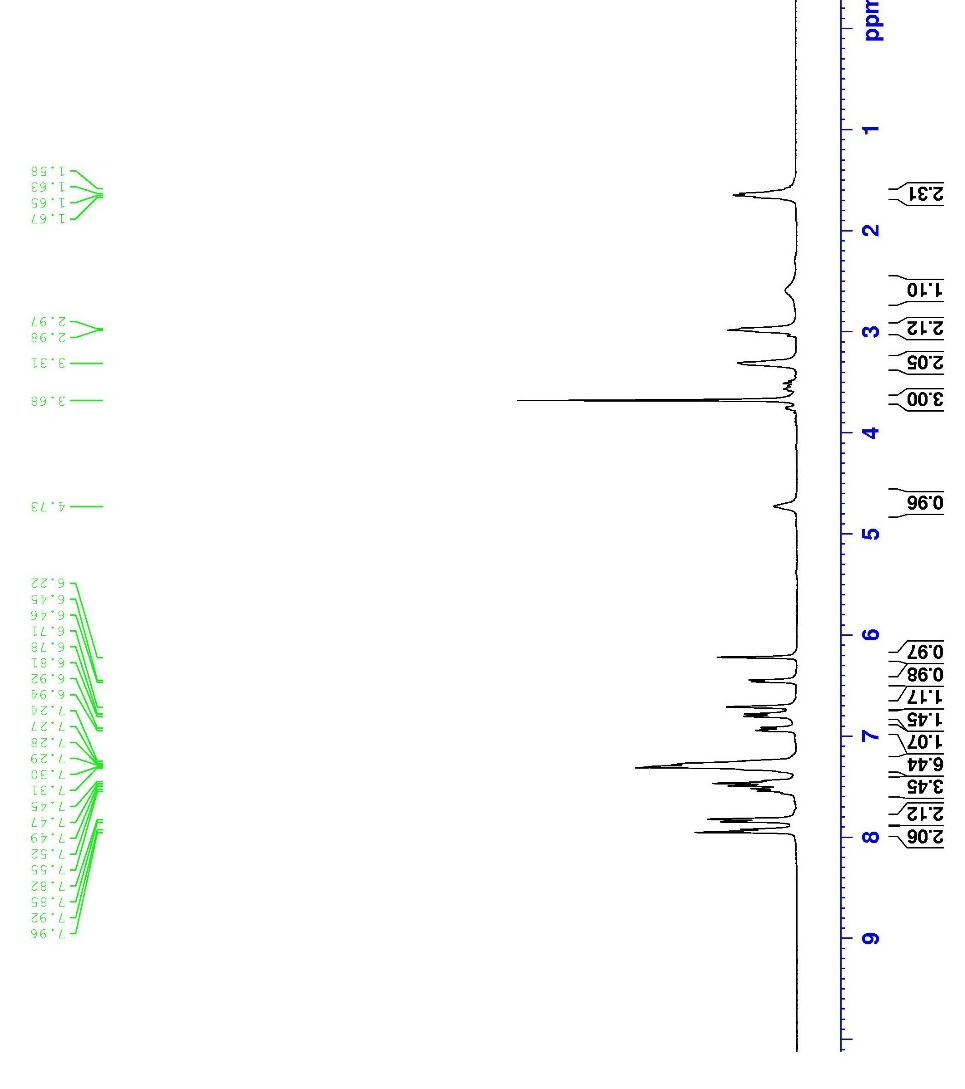


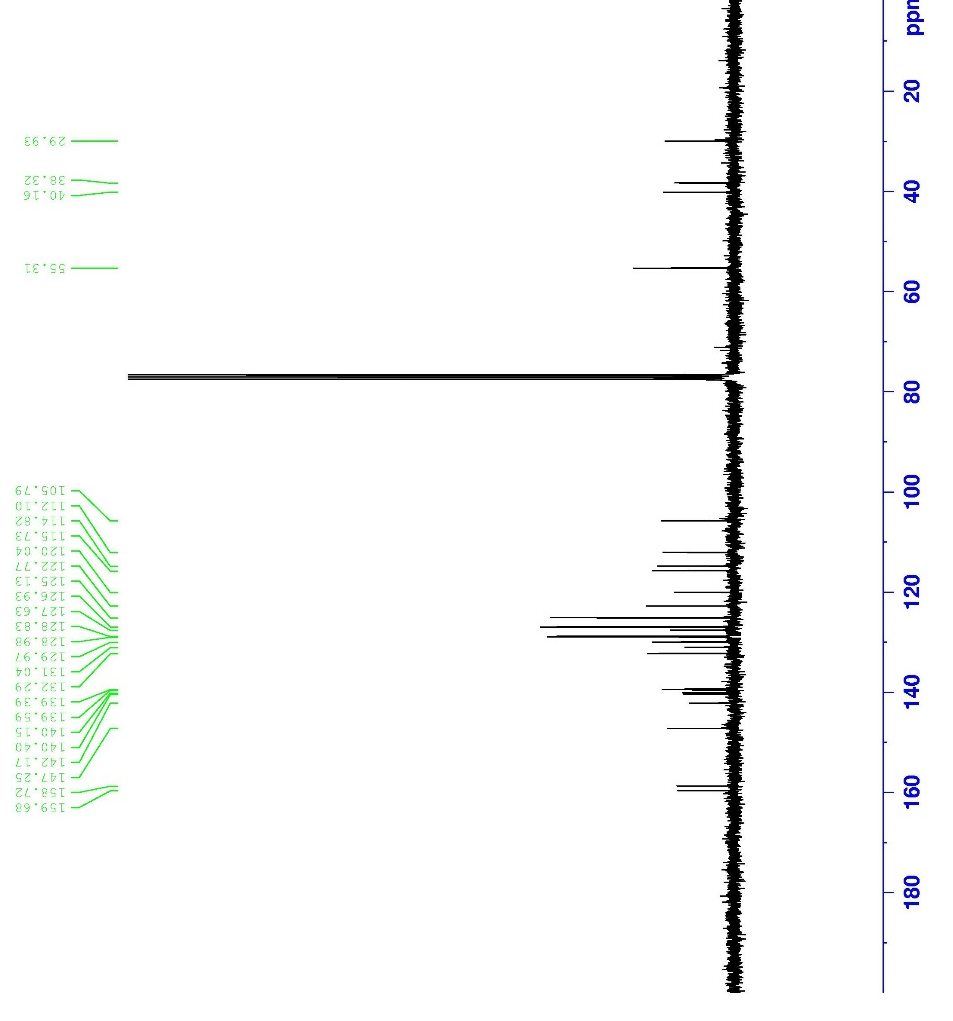


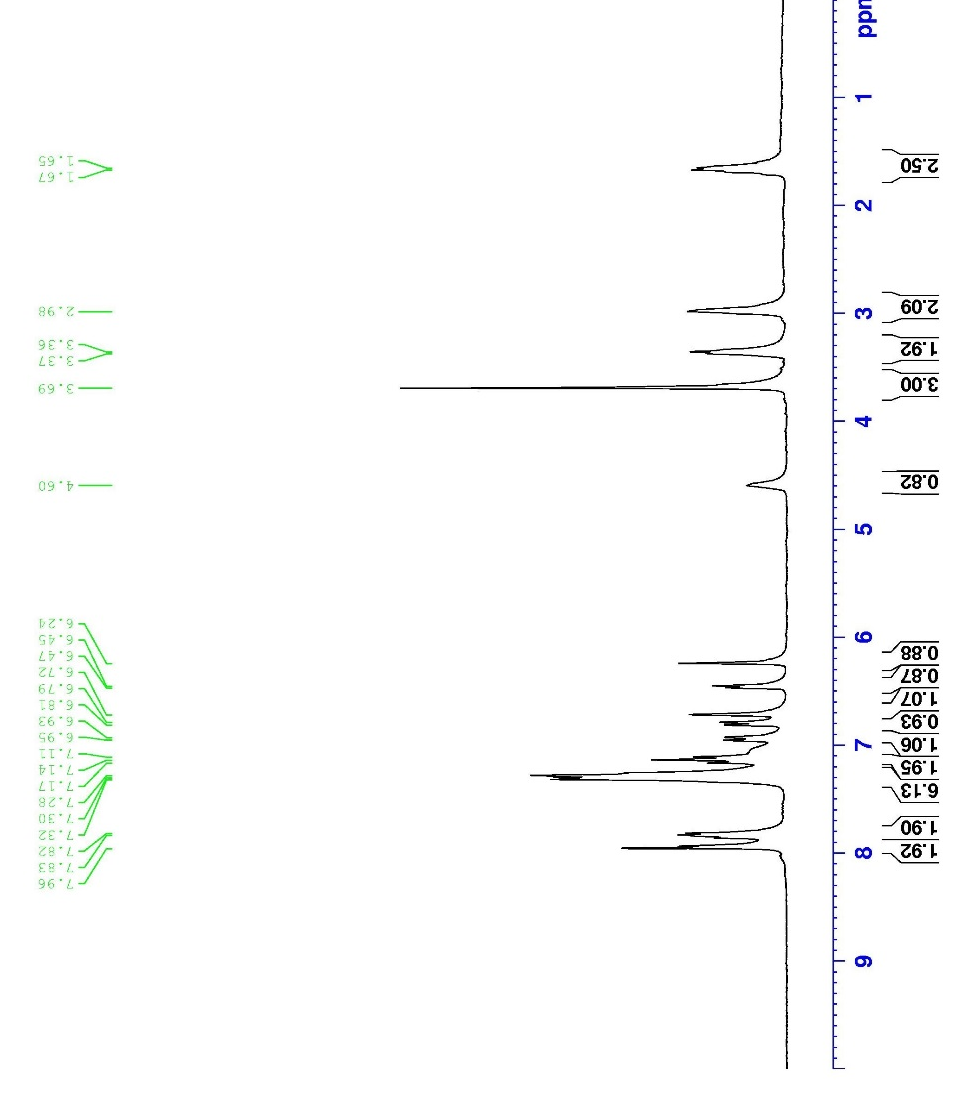


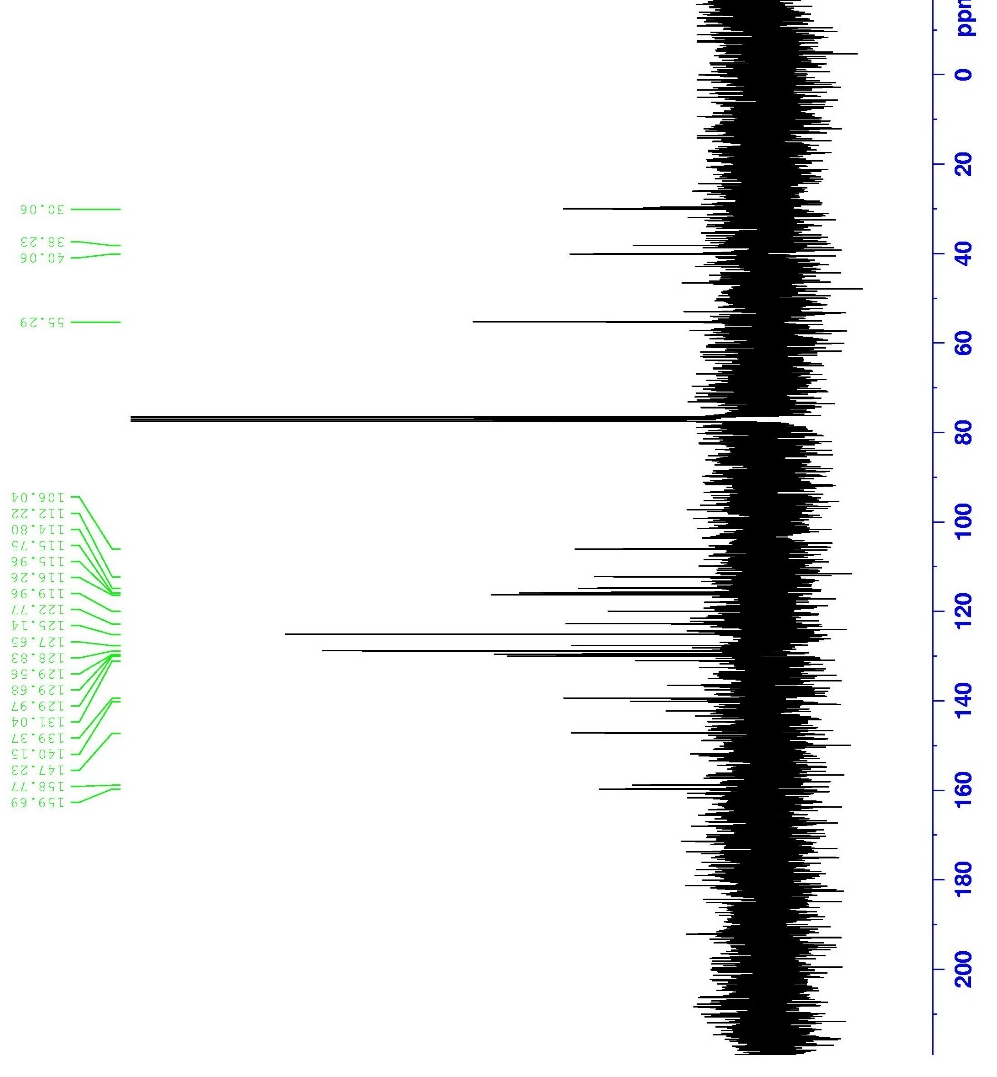


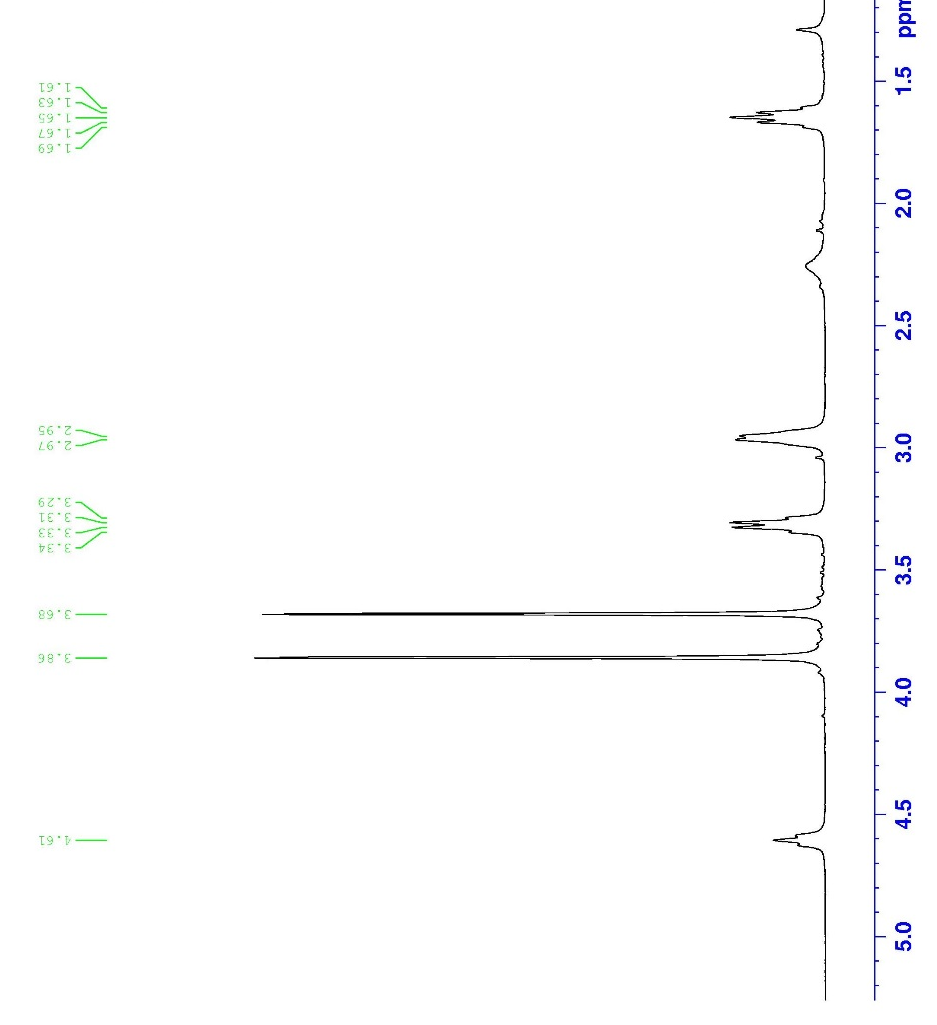


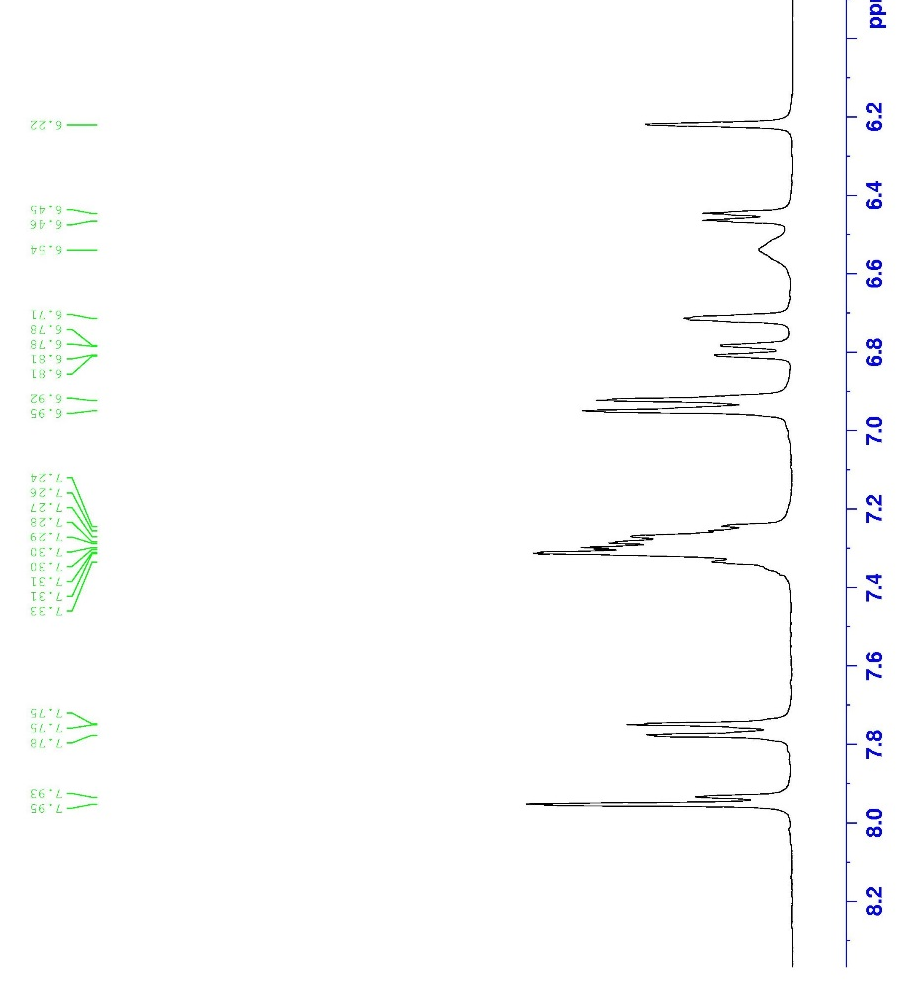


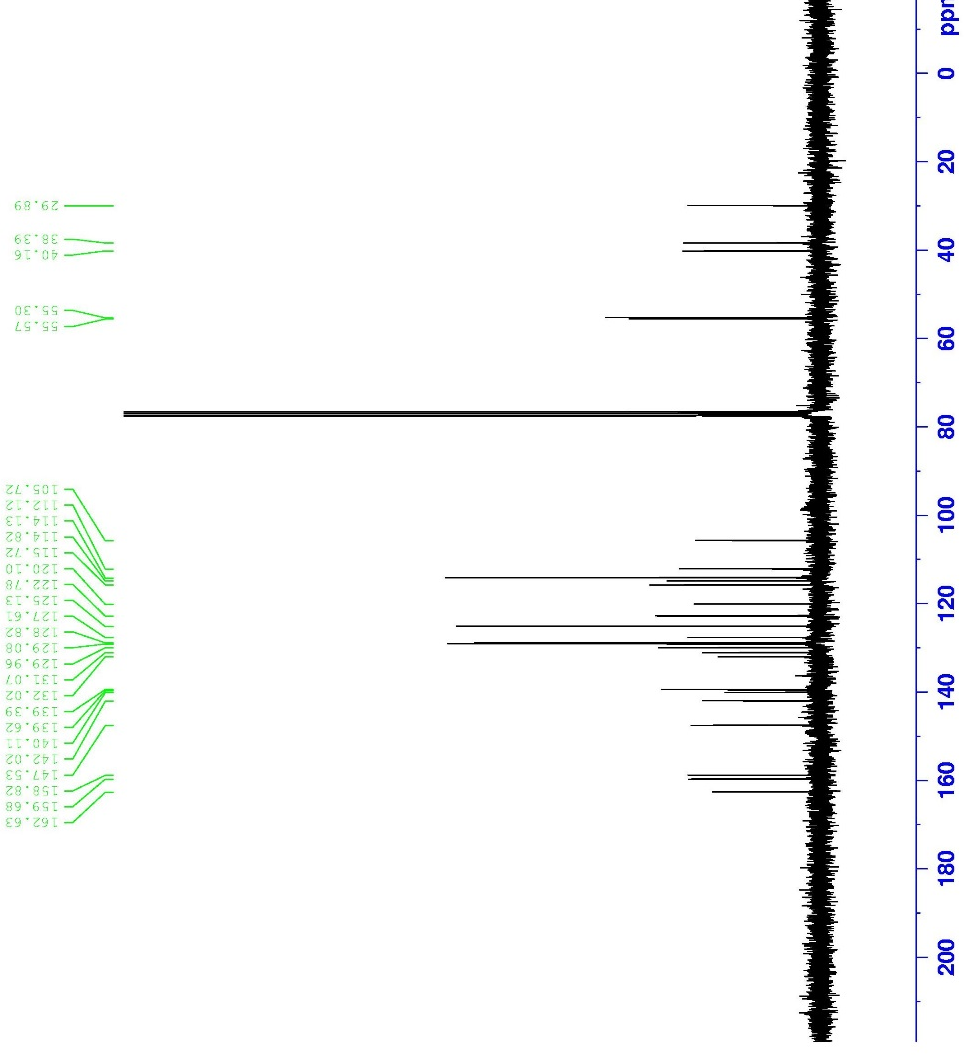


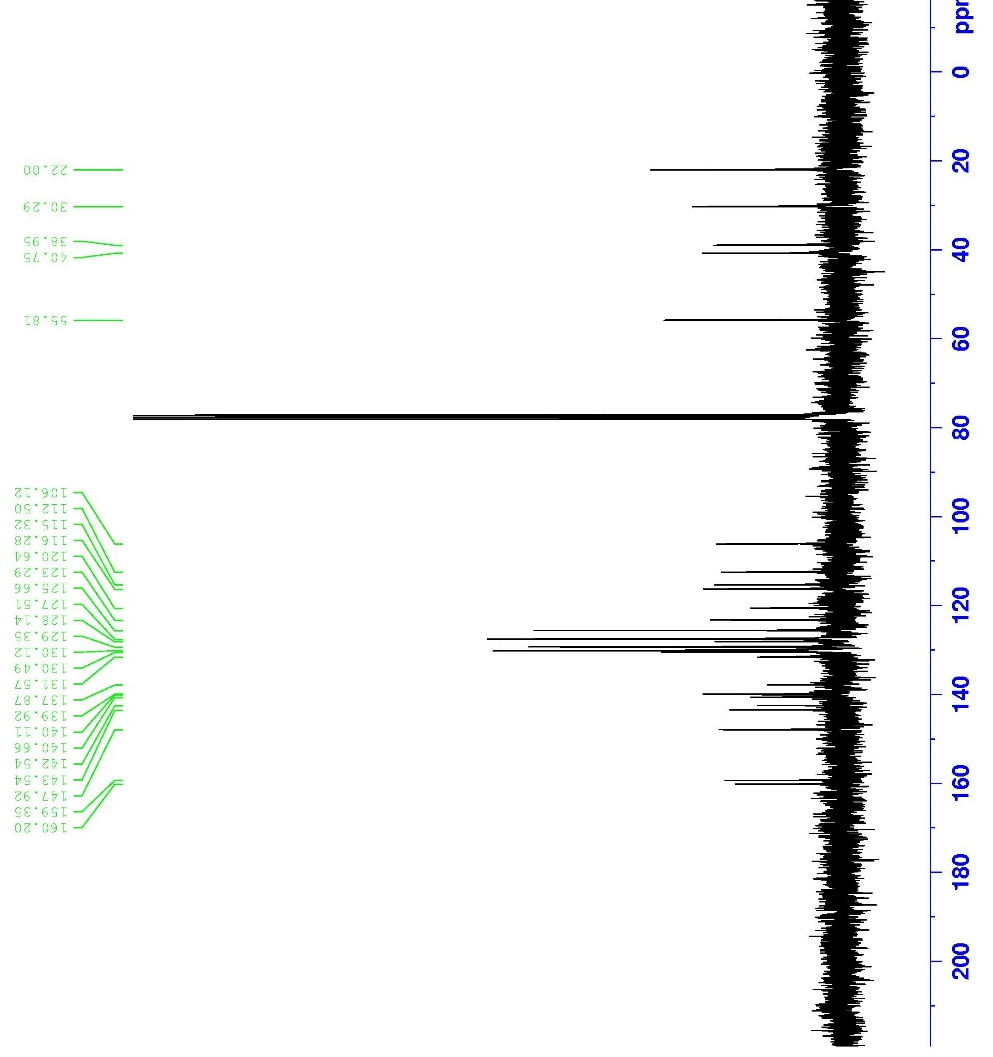


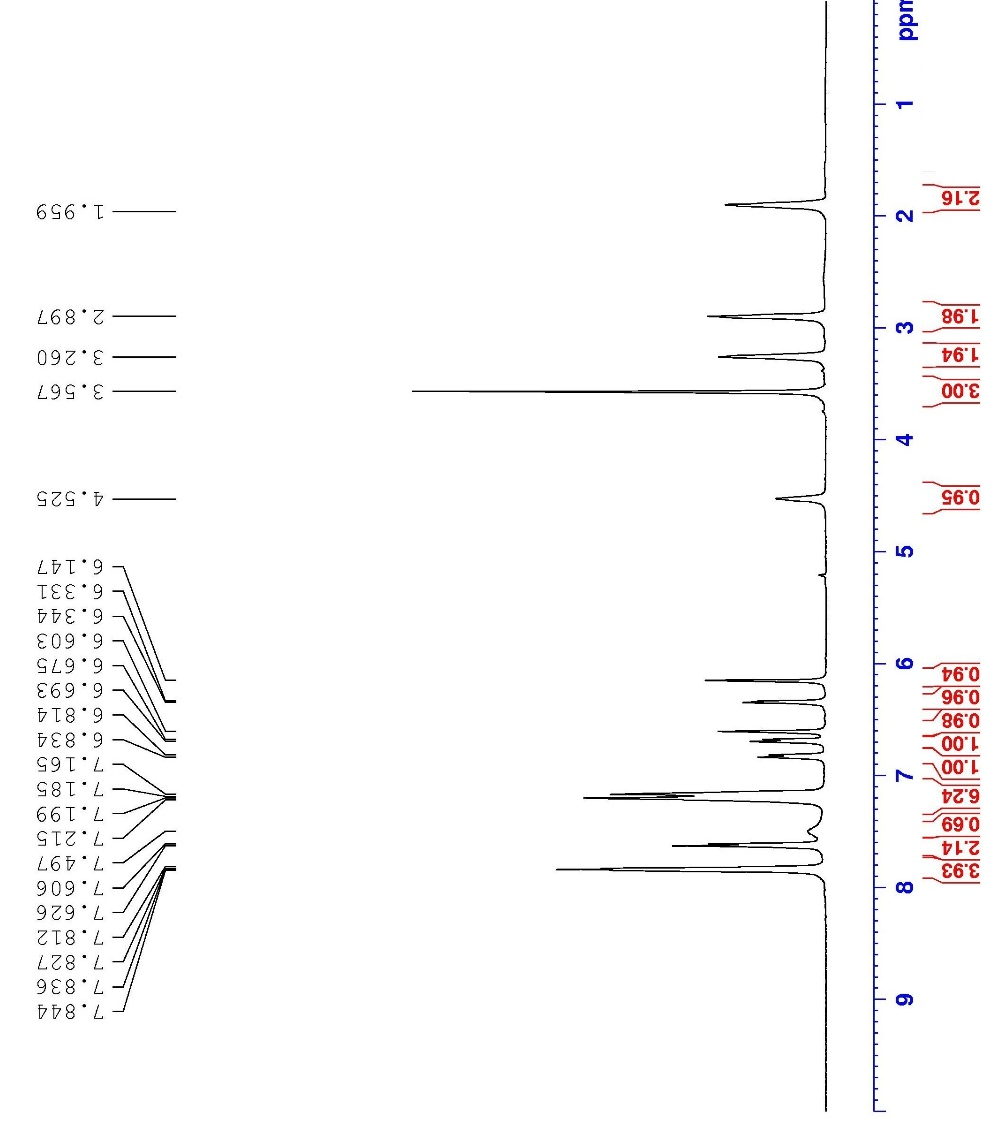


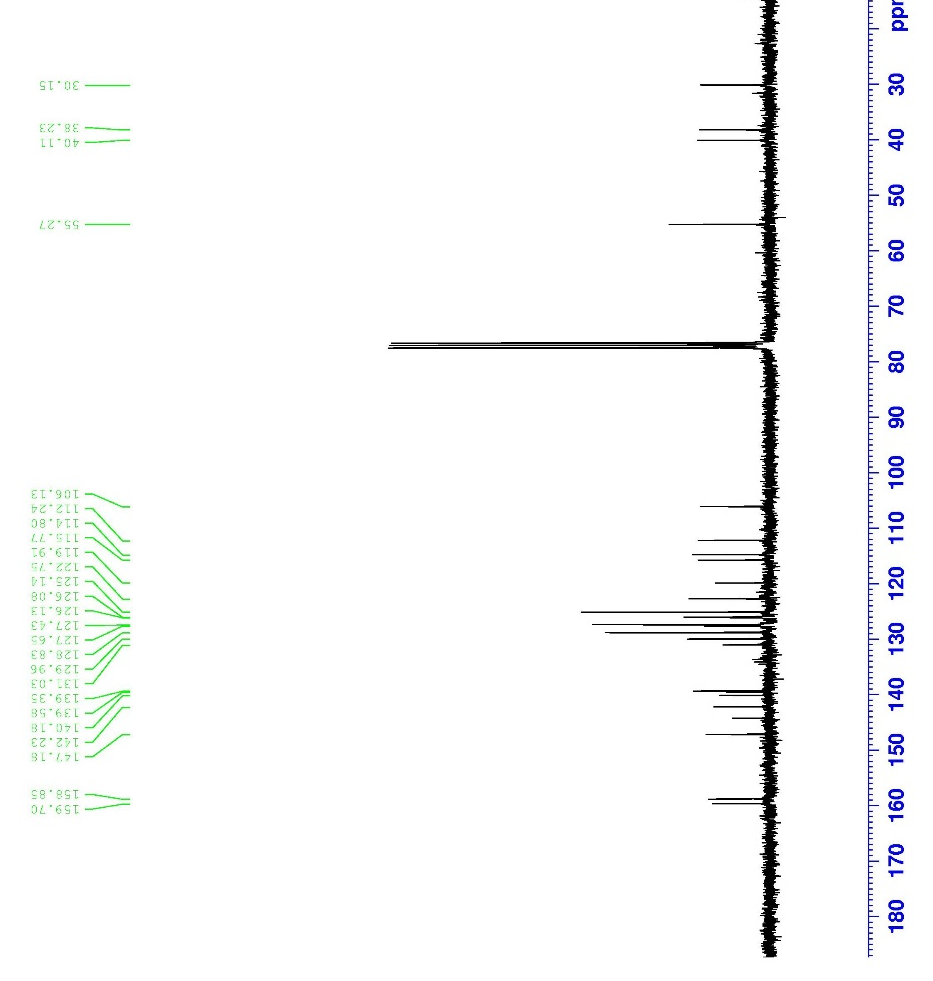


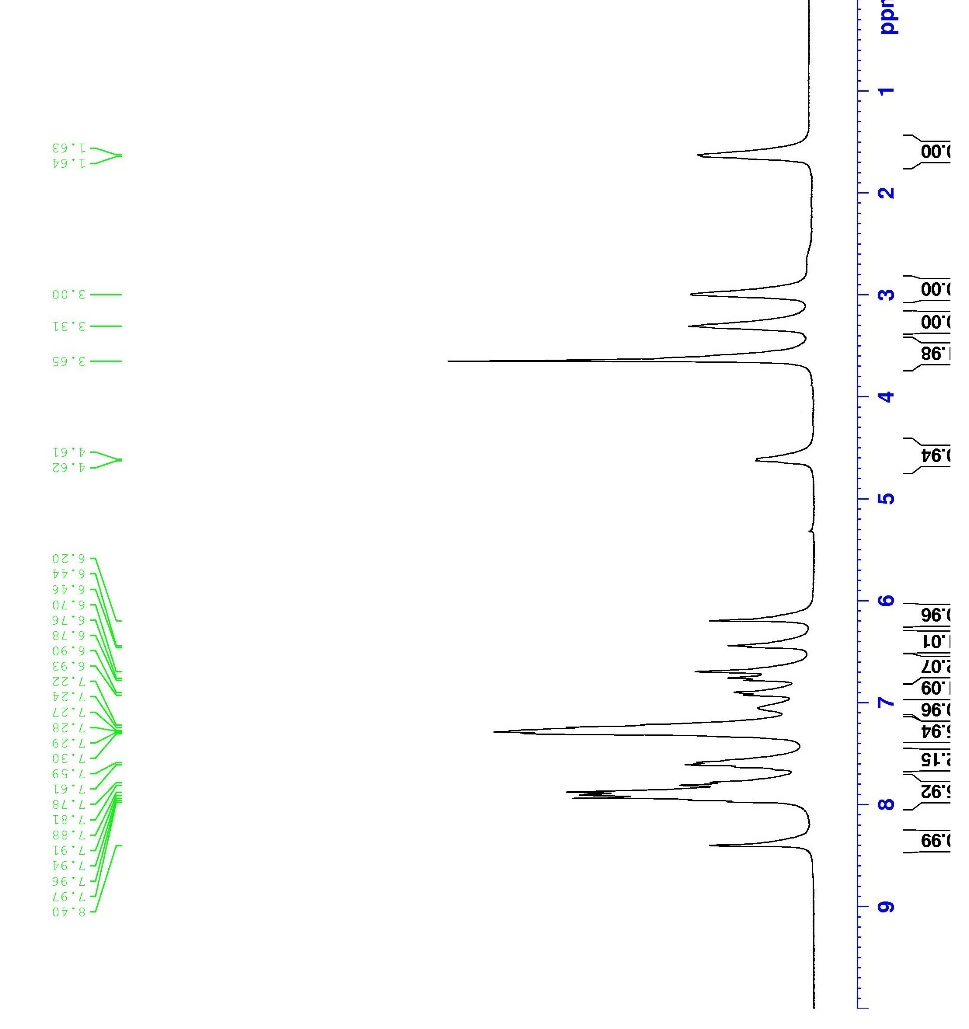


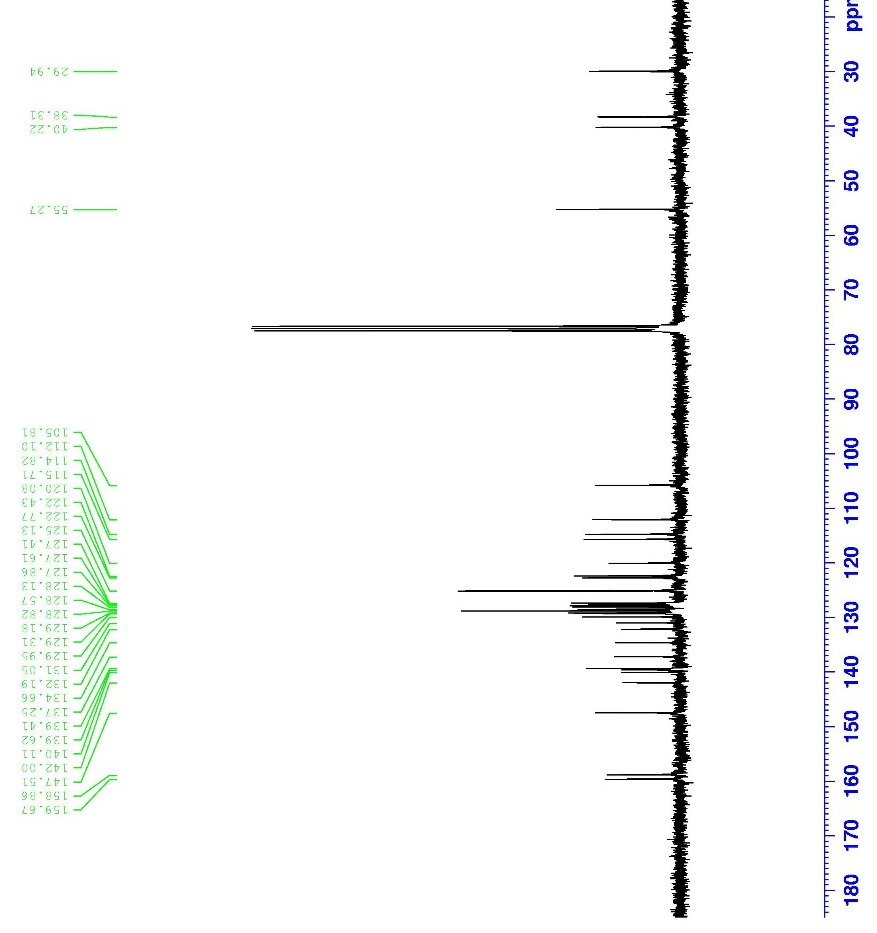

Supplement: IENZ_1530225_Supplementary Material [file IENZ_A_1530225_SM7868.doc]
